# *Fzd7* Restrains *Pink1*‐Dependent Mitophagy in Suture Stem Cells to Maintain Cranial Suture Patency

**DOI:** 10.1002/advs.77245

**Published:** 2026-08-19

**Authors:** Xinyan Chen, Zong Chen, Chenzhi Lai, Yingying Yue, Tian He, Hao Gong, Jing Shi, Xiaoshuang Guo, Yu Wang, Guodong Song, Xiaolei Jin

**Affiliations:** ^1^ Craniomaxillofacial Surgery Department Plastic Surgery Hospital Chinese Academy of Medical Sciences & Peking Union Medical College Beijing China; ^2^ Department of Plastic and Aesthetic Surgery Peking Union Medical College Hospital Chinese Academy of Medical Sciences and Peking Union Medical College Beijing China; ^3^ Center For Regenerative Medicine & Plastic Surgery Research Peking Union Medical College Hospital Beijing China; ^4^ Jishuitan Hospital Capital Medical University Beijing China

**Keywords:** craniosynostosis, mesenchymal stem cell, mitophagy, pink1

## Abstract

Craniosynostosis results from premature fusion of the cranial sutures, yet the contribution of suture mesenchymal stem cell (SuSC) dysfunction to this process remains incompletely understood. Here, we combined single‐cell RNA‐seq with 2‐µm Visium HD spatial transcriptomics to define stage‐specific changes in *Prrx1* ^+^ SuSCs in the *Fgfr2^C342Y/+^* mouse model. We observed early downregulation of *Frizzled‐7* (*Fzd7*), increased mitophagy‐associated signatures and readouts with elevated *Pink1* expression, and premature osteogenic activation within the SuSC niche. In vitro, loss of *Fzd7* increased mitophagy and osteogenic activity, whereas *Fzd7* overexpression attenuated these changes. In vivo, conditional deletion of *Fzd7* accelerated coronal suture fusion, while AAV‐mediated *Fzd7* overexpression reduced fusion. Conditional deletion of *Pink1* suppressed the effects of *Fzd7* loss, supporting *Pink1*‐dependent mitophagy as a required component of the *Fzd7*‐deficiency‐associated osteogenic and fusion phenotypes. These findings support a model in which an *Fzd7–Pink1* mitophagy axis contributes to the maintenance of suture patency in craniosynostosis and provide a mechanistic basis for future targeted studies.

## Graphical abstract

How suture stem cells fail to preserve cranial suture patency remains incompletely understood. Integrated single‐cell and high‐resolution spatial transcriptomic analyses identify reduced *Fzd7* expression in *Prrx1* ^+^ suture stem cells as an early feature of craniosynostosis. Functional perturbation shows that *Fzd7* restrains *Pink1*‐dependent mitophagy and premature osteogenic activation, thereby supporting cranial suture patency in the *Fgfr2^C342Y/+^* mouse model.

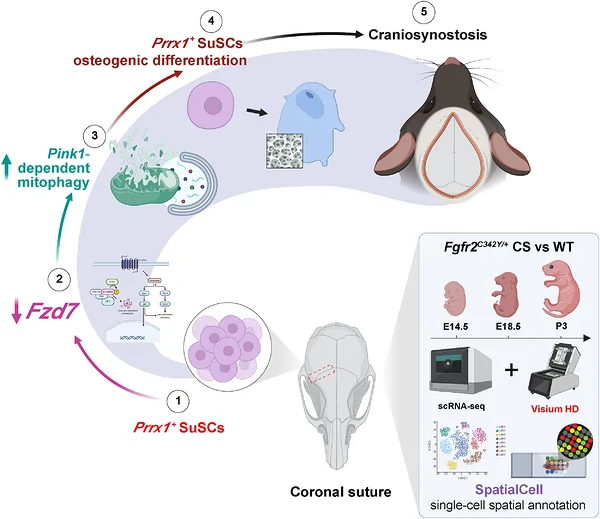

## 1 Introduction

Craniosynostosis is a craniofacial disorder caused by premature fusion of one or more cranial sutures in early life, occurring in approximately 1 in 1700–2500 live births and leading to restricted skull growth, elevated intracranial pressure, and risks of neurodevelopmental impairment [1, 2]. Surgical intervention remains the primary treatment, yet resynostosis and reoperation are common [3], underscoring the need for mechanism‐based therapies and motivating recent efforts to prevent premature suture fusion in genetic models of craniosynostosis [4]. Progress toward such strategies has been limited by an incomplete understanding of the cellular, molecular, and microenvironmental events that precipitate pathologic suture closure.

Suture mesenchymal stem cells (SuSCs)—resident stem/progenitor populations within the cranial suture midline that supply osteogenic precursors during normal growth—are critical for maintaining suture patency and osteogenic homeostasis [5, 6, 7, 8, 9]. Recent studies have identified *Prrx1* ^+^ SuSCs as a resident stem cell population of the cranial sutures and a required contributor to calvarial defect regeneration in mice [10]. However, the relationship between *Prrx1* ^+^ SuSC states and craniosynostosis has not been fully defined. Human *PRRX1* variants are associated with craniosynostosis, supporting a broader link between *PRRX1* biology and suture homeostasis [11]. The disease‐associated regulation of *Prrx1* ^+^ SuSC states within the craniosynostotic niche remains poorly understood.

Single‐cell and spatial transcriptomic approaches now enable cell‐type‐resolved, context‐aware profiling of skeletal tissues [12, 13, 14]. Yet these tools have rarely been applied to diseased sutures in a stage‐resolved manner with specific focus on SuSCs in their native niche. This gap limits mechanistic analysis of how SuSC fate changes during craniosynostosis.

In the present study, we leverage a gain‐of‐function *Fgfr2^C342Y/+^* mouse model of craniosynostosis—originally described by Eswarakumar et al. and widely used in the field [15, 16]—and use integrated single‐cell RNA‐seq and 2‐µm‐resolution Visium HD spatial transcriptomics as a discovery framework, together with lineage‐focused functional perturbation, to define how *Prrx1* ^+^ SuSC fate is altered across embryonic and postnatal development. Specifically, we generated a cross‐genotype, multi‐timepoint atlas of the coronal suture and used it to identify a candidate pathway linking reduced *Frizzled‐7* (*Fzd7*) signaling to *Pink1*‐dependent mitophagy and osteogenic activation. Guided by these insights, we perturb this axis in vitro and in vivo to determine its functional consequences for SuSC mitophagy and osteogenic output and to define a mechanistic checkpoint for suture patency. Our results may advance the understanding of *Prrx1* ^+^ SuSC contributions to suture homeostasis and inform future targeted therapeutic intervention.

## 2 Results

### 2.1 Loss of *Fzd7* in *Prrx1* ^+^ SuSCs Is Coupled With Aberrant Mitophagy and Premature Osteogenesis

**FIGURE 1:**
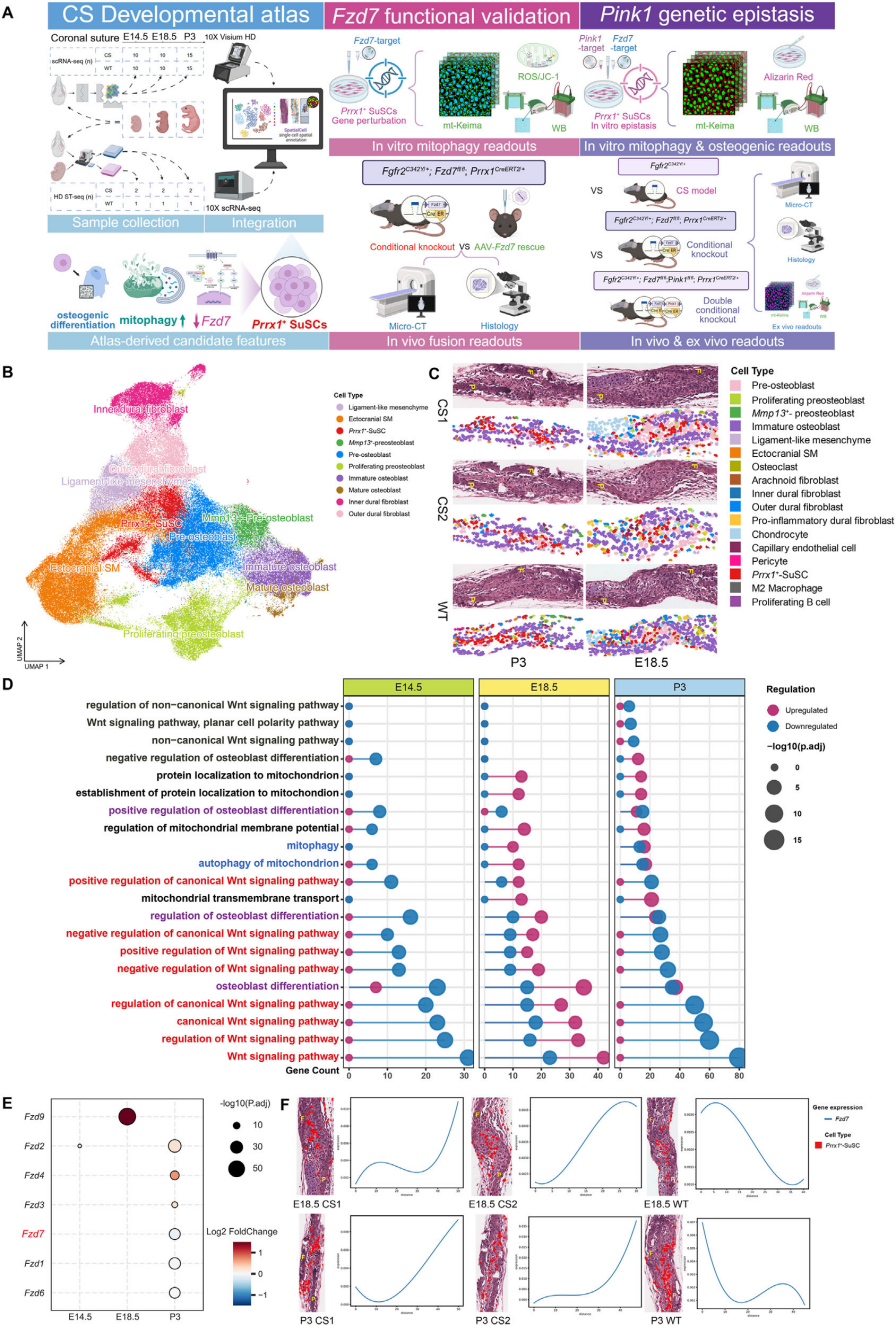
Loss of *Fzd7* in *Prrx1* ^+^ SuSCs is coupled with aberrant mitophagy and premature osteogenesis. (A) Schematic overview of the study design and principal genetic model relationships. WT and *Fgfr2^C342Y/+^* craniosynostosis (CS) samples were used to generate the developmental atlas, followed by *Fzd7* functional analysis and *Pink1* genetic epistasis testing. The schematic highlights the principal model relationships and comparison groups; complete cohorts, controls, and readouts are shown in the corresponding figures. Created in BioRender. Chen, X. (2026). (B) UMAP of integrated coronal suture scRNA‐seq data across developmental stages and genotypes, showing the major mesenchymal and niche‐associated cell populations. (C) Spatial mapping of cell types in the E18.5 and P3 samples onto hematoxylin and eosin (H&E). Spatial cell‐type maps were generated from 2‐µm Visium HD data using the SpatialCell workflow, which integrates histology‐guided nuclear segmentation, bin‐to‐cell aggregation, and reference‐based cell‐type annotation to generate reconstructed near‐single‐cell spatial units. F, frontal bone; P, parietal bone. (D) Lollipop plot illustrates Gene Ontology Biological Process (GO‐BP) enrichment for differentially expressed genes (DEGs) identified from scRNA‐seq data, comparing CS and WT samples across three developmental stages. Analyses were performed separately for upregulated and downregulated genes. (E) Dot plot illustrating the differential expression of *Frizzled* (*Fzd*) receptors in CS compared to WT samples at three developmental stages. Only significant changes (adjusted p‐value < 0.05) are shown. (F) Spatial tendency analysis for *Fzd7* expression relative to *Prrx1* ^+^‐SuSC region at E18.5 and P3. Each panel shows: left, spatial map highlighting *Prrx1* ^+^‐ SuSC region; right, polynomial regression of gene expression versus distance from SuSCs boundary. F, frontal bone; P, parietal bone.

In this study, we used the *Fgfr2^C342Y/+^* craniosynostosis model to capture early cellular and molecular changes in the coronal suture across embryonic and early postnatal development (Figure 1A). Craniosynostosis (CS) and wild‐type (WT) coronal sutures were profiled at E14.5, E18.5, and P3 by single‐cell RNA‐seq (scRNA‐seq) and 2‐µm‐resolution Visium HD spatial transcriptomics, generating a cross‐genotype, multi‐timepoint atlas with near‐single‐cell spatial annotation (Figures S1A–D and S2A, Figure 1C). We next used this atlas to examine spatiotemporal molecular changes associated with suture patency. Based on prior studies showing that suture stem cells (SuSCs) are required for maintaining suture patency and can contribute to pathological ossification [5, 6, 7, 8, 9], and reports identifying *Prrx1^+^* suture mesenchymal stem/progenitor cells as resident constituents of the cranial suture niche with regenerative capacity [5, 9], we focused our analyses on the *Prrx1^+^* SuSCs. Previously reported SuSC/MSC markers and isolation strategies define biologically important but partially overlapping stem/progenitor populations [10]. We therefore did not treat individual marker‐defined populations as mutually exclusive lineage classes. Instead, we used transcriptome‐wide clustering, differential marker‐gene profiles, and spatial localization to annotate suture mesenchymal states. In this framework, *Prrx1* ^+^ SuSCs denote a *Prrx1*‐enriched SuSC population resolved within the broader SuSC compartment and spatially localized to the suture by near‐single‐cell annotation of the Visium HD data, enabling stage‐ and genotype‐resolved analyses in their native niche context (Figure 1B, Figure S3A,B, see Method).

Gene ontology (GO) and pathway enrichment analyses of the *Prrx1^+^* SuSCs scRNA‐seq data highlighted a prominent dysregulation of Wnt signaling components across developmental stages (Figure 1D), a pathway previously implicated in cranial suture homeostasis [17]. Consistent with the suppression of canonical Wnt signaling, we observed the downregulation of multiple cognate receptors (Figure 1E). To prioritize receptor candidates with niche‐specific patterning, we next performed spatial tendency analysis [18] to assess spatial expression trends relative to the *Prrx1^+^* SuSC domain. Among the receptors examined, *Fzd7* displayed a genotype‐dependent reversal in spatial gradient: *Fzd7* expression increased with distance from the *Prrx1^+^* domain in CS, whereas the opposite trend was observed in WT (Figure 1F). We therefore focused subsequent analyses on *Fzd7*.

**FIGURE 2:**
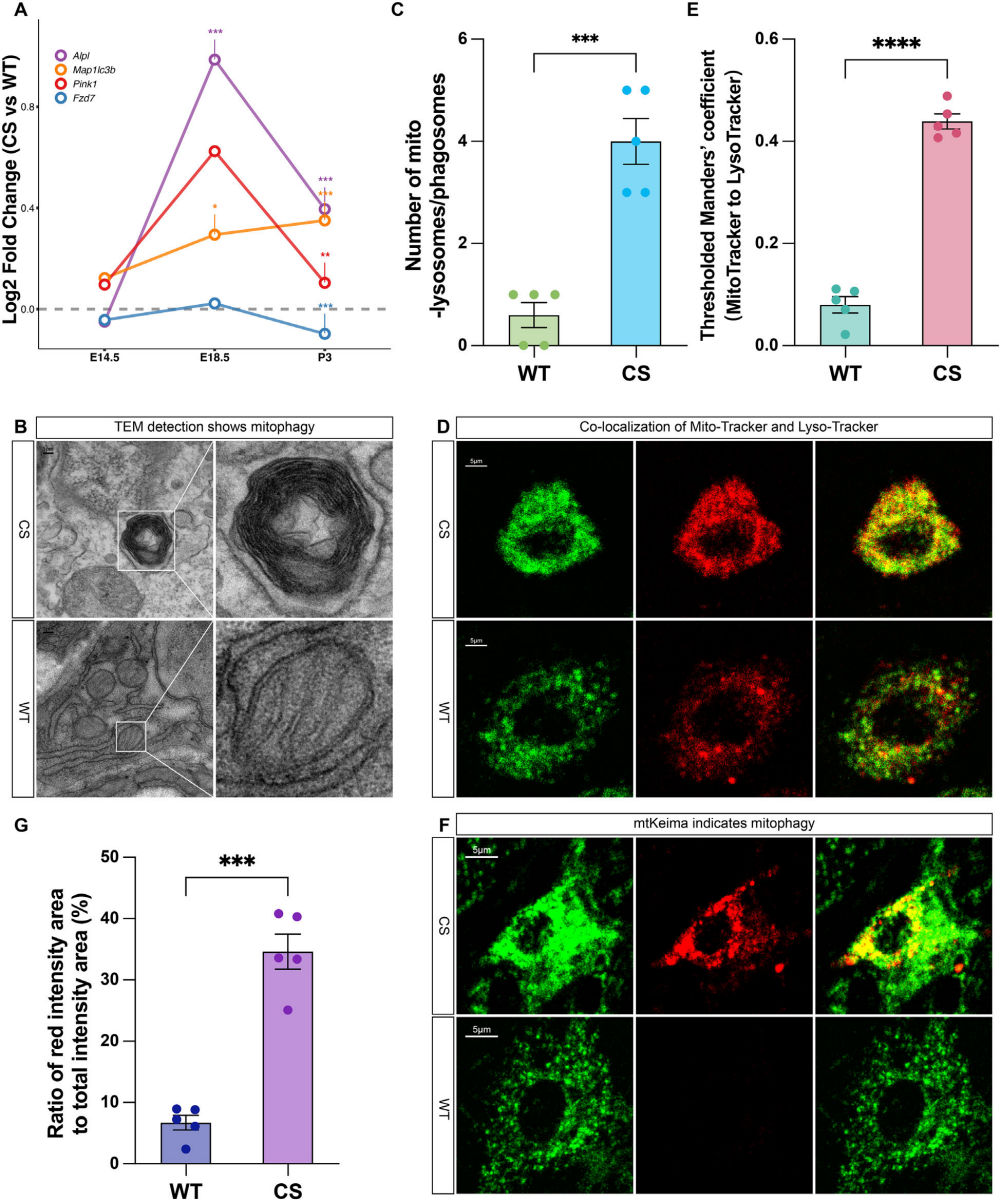
Loss of *Fzd7* in *Prrx1* ^+^ SuSCs is coupled with aberrant mitophagy and premature osteogenesis. (A) Expression trajectories of key genes in *Prrx1* ^+^ SuSCs across developmental stages. Line plot displaying average log2 fold change derived from scRNA‐seq DEGs. Asterisks denote statistical significance based on adjusted p‐values: *, *p* < 0.05; **, *p* < 0.01; ***, *p* < 0.001. CS, *Fgfr2^C342Y/+^* craniosynostosis model; WT, wild‐type. (B, C) (B) Representative TEM images of the P3 coronal suture midline showing mitochondria‐like structures enclosed within autophagosomes/autolysosomes in CS and WT mice. Scale bar, 1 µm. (C) Statistical analysis of the number of mitochondria(‐like) structures within autophagosomes/autolysosomes among the CS and WT groups. *n* = 5. (D, E) Mitochondria‐lysosome co‐localization in P3 *Prrx1* ^+^ SuSCs assessed by live‐cell staining with Mito‐Tracker (green) and Lyso‐Tracker (red). (D) Representative images of MitoTracker Green and LysoTracker Red staining from CS and WT mice. Merged images show mitochondria–lysosome colocalization. Scale bar, 5 µm. (E) Quantification of mitochondria–lysosome colocalization by thresholded Manders’ coefficient (Mito‐Tracker to Lyso‐Tracker) from CS and WT. *n* = 5. (F, G) Visualization of mtKeima in P3 *Prrx1^+^* SuSCs. (F) Representative images of mtKeima from CS and WT. Green indicates the neutral mitochondrial signal, and red indicates the lysosomal/acidic mtKeima signal. Scale bar, 5 µm. (G) Quantification of mtKeima mitophagy readout as the ratio of red intensity area to total mtKeima intensity area [red/(red + green)] from CS and WT mice. *n* = 5.

Temporal scRNA‐seq profiling across stages suggested that E18.5 represents an inflection point in CS *Prrx1^+^* SuSCs, with concurrent emergence of osteogenic‐ and mitophagy‐related signatures (Figures 1D and 2A, Data S1). At E18.5, osteogenic priming was supported by enrichment of osteoblast differentiation‐related terms and increased *Alpl* expression (Figures 1D and 2A). In parallel, mitophagy‐associated terms, including “mitophagy” and “autophagy of mitochondrion,” were enriched (Figure 1D), accompanied by upregulation of *Map1lc3b* and *Pink1*. By P3, this pattern persisted, with *Fzd7* downregulation and sustained elevation of *Pink1* (Figure 2A).

Since these mitophagy‐ and osteogenic‐priming signatures persisted through P3, we employed transmission electron microscopy (TEM) in situ to examine the suture mesenchyme of P3 mice, investigating whether these transcriptomic signatures correspond to ultrastructural alterations in organelles. In cells located within the CS suture mesenchyme midline, we observed an increased abundance of autophagosomes/autolysosomes containing mitochondrial material in CS compared with WT controls (Figure 2B,C). Because this TEM analysis was performed in the mid‐sutural mesenchyme, it provided region‐level ultrastructural evidence rather than direct *Prrx1* ^+^ SuSC‐specific evidence. This region contains *Prrx1* ^+^ SuSCs together with other mesenchymal populations. We therefore next examined FACS‐purified P3 *Prrx1* ^+^ SuSCs. Live‐cell staining with Mito‐Tracker and Lyso‐Tracker showed increased mitochondria‐lysosome colocalization in CS‐derived *Prrx1* ^+^ SuSCs relative to WT, quantified by thresholded Manders’ coefficients (Figure 2D,E).

To further distinguish between static accumulation and active mitophagic flux, we utilized the mtKeima biosensor [19] in FACS‐purified *Prrx1^+^* SuSCs isolated from P3 sutures. CS‐derived cells exhibited a higher fraction of mtKeima signal in the lysosomal (red) channel, quantified as the red‐to‐total (red + green) intensity area ratio, compared with WT controls, indicating increased delivery of mitochondria to lysosomes (Figure 2F,G). Collectively, these multi‐modal data provide evidence at complementary levels, from regional ultrastructural changes in the CS suture mesenchyme to increased mitophagy‐associated colocalization and flux readouts in purified CS *Prrx1* ^+^ SuSCs, in association with a *Fzd7*‐low, osteogenically primed state.

### 2.2 In Vitro *Fzd7* Modulation Alters PINK1–PARKIN–Linked Mitophagy Markers in *Prrx1* ^+^ SuSCs

**FIGURE 3:**
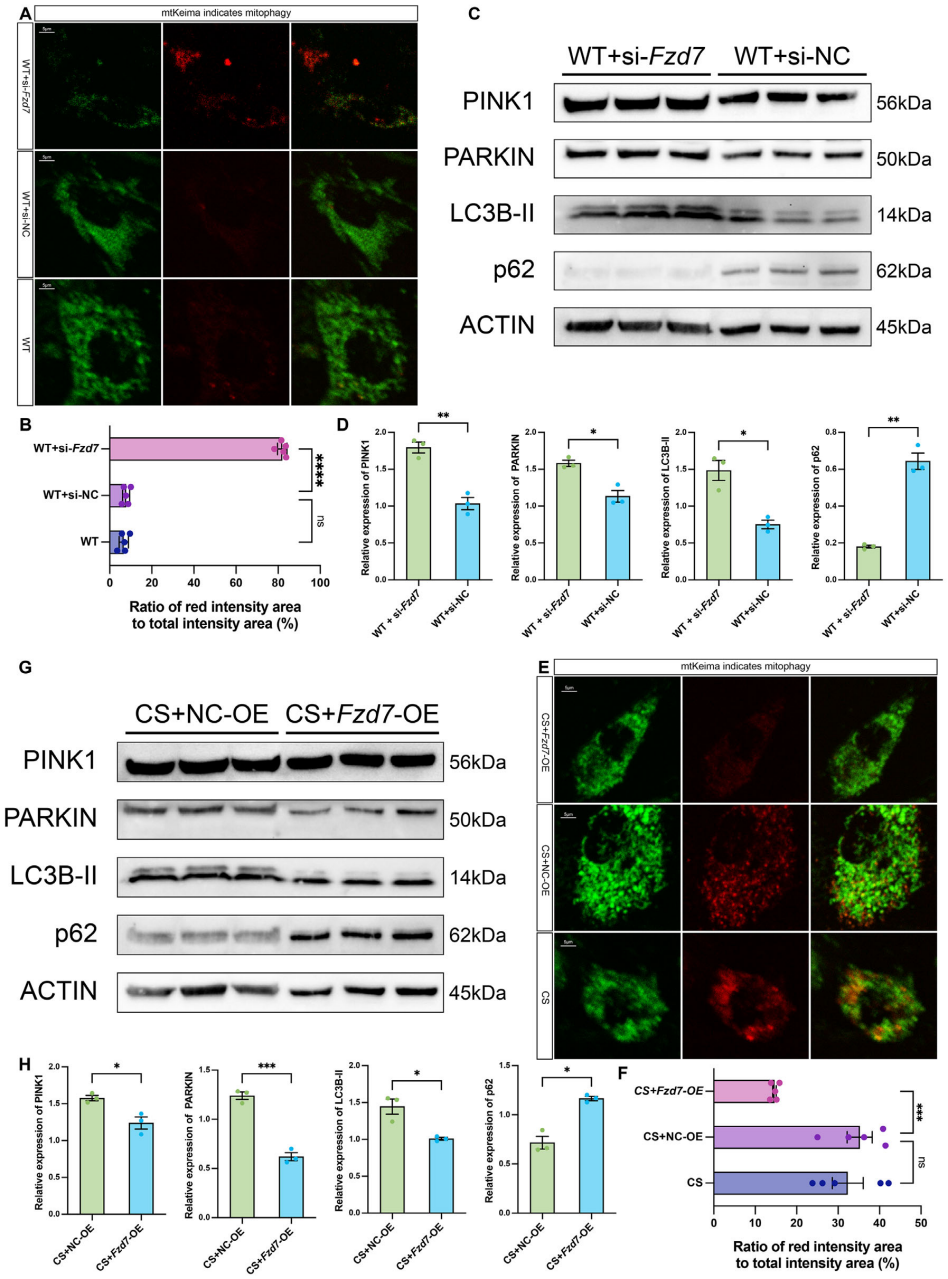
In vitro *Fzd7* modulation alters PINK1–PARKIN–linked mitophagy markers in *Prrx1* ^+^ SuSCs. (A, B) Visualization of mtKeima in E18.5 *Prrx1* ^+^ SuSCs with siRNA treatments. (A) Representative mtKeima images. Green indicates the neutral mitochondrial signal, and red indicates the lysosomal/acidic mtKeima signal. Scale bar, 5 µm. (B) Quantification of the mtKeima mitophagy readout as the ratio of red intensity area to total mtKeima intensity area [red/(red + green)]. *n* = 5. (C, D) Western blotting analyses of mitophagy related protein (C) and the quantification of protein expression (D) of WT+si‐*Fzd7* and WT + si‐NC of E18.5 *Prrx1* ^+^ SuSCs. *n* = 3. (E, F) Visualization of mtKeima in E18.5 *Prrx1* ^+^ SuSCs with *Fzd7* overexpression treatments. (E) Representative mtKeima images. Green indicates the neutral mitochondrial signal, and red indicates the lysosomal/acidic mtKeima signal. Scale bar, 5 µm. (F) Quantification of the mtKeima mitophagy readout as the ratio of red intensity area to total mtKeima intensity area [red/(red + green)]. *n* = 5. (G, H) Western blotting analyses of mitophagy related protein (G) and the quantification of protein expression (H) of CS + *Fzd7*‐OE and CS + NC‐OE of E18.5 *Prrx1* ^+^ SuSCs. *n* = 3.

Guided by the atlas‐defined *Fzd7*‐low state with increased mitophagy‐associated signatures and *Pink1* expression in CS *Prrx1* ^+^ SuSCs, we next asked whether manipulating *Fzd7* in *Prrx1* ^+^ SuSCs is sufficient to alter mitophagy activities in vitro. We generated *Fgfr2^+/+^*; *Prrx1^EGFP/+^* mice (hereafter WT; *Prrx1‐EGFP* reporter mice) and FACS‐isolated *Prrx1‐EGFP^+^* SuSCs at E18.5. Compared with non‐targeting controls, *Fzd7* silencing by siRNA markedly increased the lysosome‐shifted mtKeima signal, quantified as the ratio of red intensity area to total mtKeima intensity area (Figure 3A,B). This change was accompanied by increased abundance of PINK1, PARKIN, and LC3B‐II and reduced p62 (Figure 3C,D), consistent with elevated mitophagy‐associated protein programs upon *Fzd7* reduction. We next assessed whether the enhanced mitophagy‐associated readouts were accompanied by ROS accumulation or mitochondrial membrane potential loss. Intracellular ROS levels were measured using DCFH‐DA‐based fluorescence, and mitochondrial membrane potential was quantified using the JC‐1 aggregate/monomer fluorescence ratio. In the disease‐state comparison, CS‐derived *Prrx1* ^+^ SuSCs did not show a significant increase in DCF fluorescence compared with WT cells, whereas positive‐control treatment induced the expected increase in ROS signal in both WT and CS cells. Similarly, CS‐derived *Prrx1* ^+^ SuSCs did not show a significant reduction in the JC‐1 aggregate/monomer ratio compared with WT cells, whereas positive‐control treatment reduced this ratio as expected. In the knockdown setting, WT+si‐*Fzd7* cells did not show a significant increase in DCF fluorescence or a significant reduction in the JC‐1 aggregate/monomer ratio compared with WT+si‐NC cells (Figure S8A–D). These data indicate that the enhanced mitophagy‐associated readouts in CS‐derived and *Fzd7*‐deficient *Prrx1* ^+^ SuSCs were not accompanied by detectable ROS accumulation or mitochondrial depolarization under these assay conditions. Because FZD7 can participate in both β‐catenin‐dependent and β‐catenin‐independent Wnt signaling [20, 21, 22], we further examined Wnt pathway‐associated protein readouts after *Fzd7* silencing in WT *Prrx1* ^+^ SuSCs. *Fzd7* knockdown reduced canonical Wnt/β‐catenin‐associated readouts, including active β‐catenin, AXIN2, and Cyclin D1, and increased PCP/JNK‐associated readouts, including p‐JNK and VANGL1 (Figure S9A,B). These data indicate that *Fzd7* loss is accompanied by altered Wnt pathway‐associated protein states in *Prrx1* ^+^ SuSCs, but they do not by themselves define a branch‐specific signaling route from FZD7 to *Pink1*‐dependent mitophagy.

We next tested whether restoring *Fzd7* could normalize these mitophagy‐associated abnormalities observed in CS‐derived SuSCs. *Prrx1‐EGFP* ^+^ SuSCs were FACS‐sorted from E18.5 *Fgfr2^C342Y/+^*; *Prrx1^EGFP/+^* mice (CS; *Prrx1‐EGFP* reporter mice) and transduced with AAV‐*Fzd7* or control vector. Relative to CS vector controls, *Fzd7* overexpression decreased the lysosome‐shifted mtKeima signal (Figure 3E,F) and was associated with reduced PINK1, PARKIN, and LC3B‐II levels together with restored p62 (Figure 3G,H). Together, these in vitro perturbation experiments indicate that *Fzd7* regulates mitophagy‐associated readouts in *Prrx1* ^+^ SuSCs.

### 2.3 In Vivo *Fzd7* Perturbation Alters Coronal Suture Closure

**FIGURE 4:**
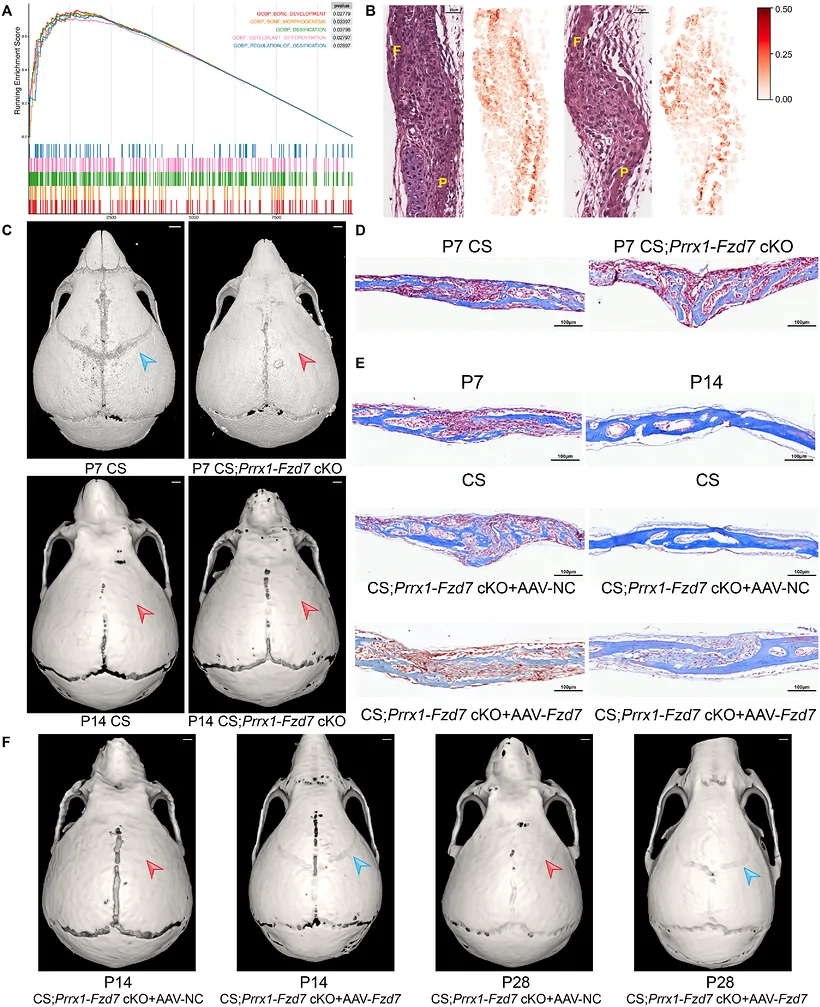
Altered *Fzd7* expression modulates coronal suture closure in vivo. (A). GSEA plots showing the enrichment of bone formation‐related terms among highly perturbed genes in *Fzd7* virtual knockout (KO). Enrichment indicates significant regulatory network changes. The colors represent different osteogenic pathways as indicated in the top‐right table. p‐value for each pathway is provided in the inset table. (B). Spatial visualization of the osteogenic pathway activity (GOBP_OSSIFICATION) on E18.5 CS coronal sutures following *Fzd7* virtual knockout. The metagene was constructed using the core enrichment subset identified from GSEA of *Fzd7* KO vs WT. F, frontal bone; P, parietal bone. Scale bar: 25 µm. (C). Micro‐CT images of skulls from CS and CS;*Prrx1‐Fzd7* cKO mice at P7 and P14. Red arrowheads indicate coronal suture fusion, whereas blue arrowheads indicate a patent coronal suture. *n* = 5. CS, *Fgfr2^C342Y/+^* craniosynostosis model. Scale bar: 1 mm. (D) Masson staining images of coronal sutures from CS and CS;*Prrx1*‐*Fzd7* cKO mice at P7. Blue indicates bone, red indicates unmineralized tissue including fibrosis and cells. Scale bar: 100 µm. *n* = 5. (E) Masson staining images of coronal sutures from CS, CS;*Prrx1*‐*Fzd7* cKO + AAV‐NC, and CS;*Prrx1*‐*Fzd7* cKO + AAV‐*Fzd7* mice at P7 and P14. Blue indicates bone, red indicates unmineralized tissue including fibrosis and cells. AAV‐*Fzd7*: AAV‐mediated *Fzd7* overexpression. Scale bar: 100 µm. *n *= 5. (F) Micro‐CT images of skulls from CS;*Prrx1*‐*Fzd7* cKO + AAV‐NC and CS;*Prrx1*‐*Fzd7* cKO+AAV‐*Fzd7* mice at P14 and P28. Red arrowheads indicate coronal suture fusion, whereas blue arrowheads indicate a patent coronal suture. *n* = 5. Scale bar: 1 mm.

To assess whether reduced *Fzd7* in *Prrx1* ^+^ SuSCs contributes to coronal suture fusion in the CS context, we first performed in silico perturbation analysis using scTenifoldKnk [23] on E18.5 WT *Prrx1* ^+^ SuSC networks. Gene set enrichment analysis (GSEA) [24] of the ranked perturbation list revealed significant enrichment of multiple bone formation‐related Gene Ontology Biological Process terms, including bone development, bone morphogenesis, ossification, and osteoblast differentiation (Figure 4A). To further localize these predicted changes in situ, we extracted the leading‐edge genes (the core enrichment subset) from these osteogenic pathways and constructed an osteogenic metagene using the Banksy algorithm [25]. By mapping this metagene's activity onto E18.5 CS coronal suture, elevated osteogenic pathway activity concentrated near the osteogenic fronts was observed (Figure 4B, Figure S5A), which prompted us to test this prediction in vivo.

We generated *Fgfr2^C342Y/+^*; *Fzd7^fl/fl^*; *Prrx1^CreERT2/+^* mice (hereafter CS; *Prrx1*‐*Fzd7* cKO) and littermate CS controls. High‐resolution micro‐CT analysis demonstrated that, at P7, CS; *Prrx1*‐*Fzd7* cKO mice exhibited apparent coronal suture fusion, whereas the coronal suture remained patent in CS controls (Figure 4C). By P14, both groups displayed suture fusion, indicating that loss of *Fzd7* accelerates the timing of coronal suture closure in the CS context (Figure 4C). Consistent with the micro‐CT findings, Masson staining at P7 revealed more continuous osteoid/bone bridging across the suture midline in CS; *Prrx1*‐*Fzd7* cKO mice compared with CS controls (Figure 4D).

We next asked whether restoring *Fzd7* locally could mitigate the accelerated closure phenotype. CS; *Prrx1*‐*Fzd7* cKO mice received perisutural AAV‐mediated *Fzd7* overexpression (AAV‐*Fzd7*) or control vector at P0 (within 12 h after birth). Western blot analysis of the injected coronal suture 48 h after P0 AAV delivery showed increased FZD7 protein abundance in AAV‐Fzd7–treated mice compared with AAV‐control mice, confirming local FZD7 upregulation during the early postnatal window (Figure S14). Histological assessment showed that AAV‐*Fzd7* treatment reduced osteoid/bone bridging and preserved the midline gap at P7 and P14 compared with AAV‐control animals (Figure 4E). Micro‐CT analysis further supported this rescue effect, with AAV‐*Fzd7*–treated mice showing sustained coronal suture patency at P14 and persisting through P28, whereas AAV‐control littermates exhibited fusion (Figure 4F).

Together, the accelerated fusion observed after *Fzd7* conditional deletion and its attenuation by local AAV‐*Fzd7* restoration clarify the reciprocal phenotypic relationship between *Fzd7* loss and restoration in the CS context. These in vivo comparisons support a functional role for *Fzd7* in restraining premature coronal suture closure.

### 2.4 *Pink1* Is Required for *Fzd7*‐Loss–Induced Mitophagy and Osteogenic Activation in *Prrx1^+^* SuSCs

Building on the directional changes observed with *Fzd7* knockdown and overexpression in vitro above, we asked whether these effects are *Pink1*‐dependent. We designed an in vitro epistasis framework in FACS‐sorted E18.5 WT *Prrx1*‐EGFP^+^ SuSCs, comparing *Fzd7* knockdown alone, *Pink1* knockdown alone, co‐silencing of both genes, and non‐targeting controls. To contextualize the magnitude of pathway engagement, we analyzed CS‐derived SuSCs in parallel in the immunoblot assays as a disease‐reference benchmark (CS and CS + si‐NC).

**FIGURE 5:**
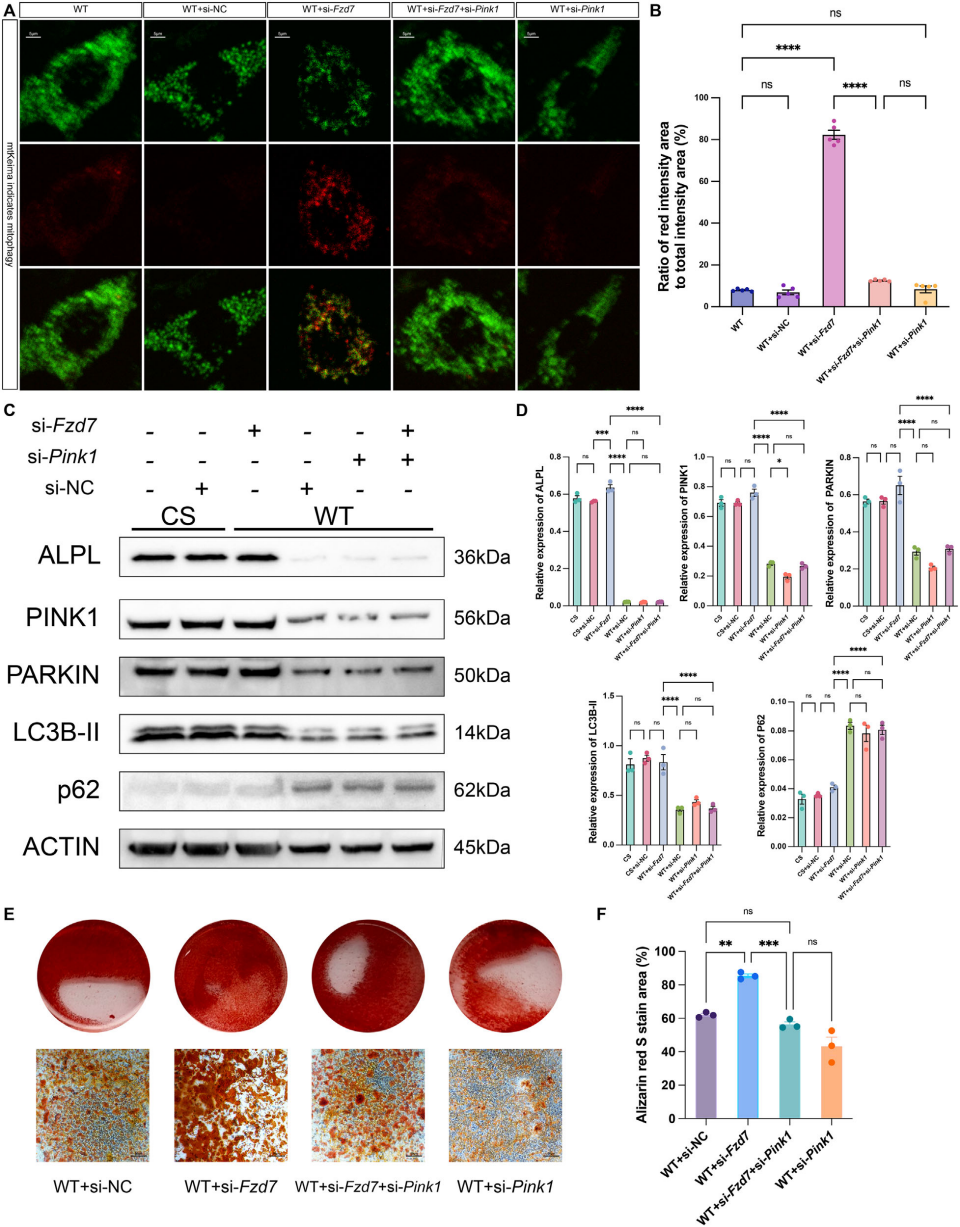
*Pink1* is required for *Fzd7*‐loss–induced mitophagy and osteogenic activation in *Prrx1* ^+^ SuSCs. (A, B) Visualization of mtKeima in E18.5 *Prrx1* ^+^ SuSCs with siRNA treatments. (A) Representative mtKeima images. Green indicates the neutral mitochondrial signal, and red indicates the lysosomal/acidic mtKeima signal. Scale bar, 5 µm. (B) Quantification of the mtKeima mitophagy readout as the ratio of red intensity area to total mtKeima intensity area [red/(red + green)]. *n* = 5. (C, D) Western blotting analyses of osteogenic and mitophagy‐related proteins (C) and the quantification of protein expression (D) of CS and WT with siRNA treatments. *n* = 3. (E, F) Alizarin Red S staining and quantification of calcium deposition in E18.5 coronal suture mesenchyme cultures from WT mice after 14 days of osteogenic induction and siRNA treatments. *n* = 3. Significance of all data above was determined by one‐way ANOVA, **p < 0.01, ***p < 0.001, and ****p < 0.0001.

In WT *Prrx1* ^+^ SuSCs, *Fzd7* silencing increased the lysosome‐shifted mtKeima signal, quantified as the ratio of red intensity area to total mtKeima intensity area (red+green), relative to WT and WT + si‐NC controls (Figure 5A,B). Notably, co‐silencing *Pink1* in the *Fzd7* knockdown background attenuated this mtKeima lysosomal shift compared with *Fzd7* knockdown alone, approaching levels observed in *Pink1* knockdown and control conditions. *Pink1* knockdown alone did not increase the mtKeima lysosomal signal relative to controls.

Consistent with these imaging readouts, immunoblotting showed that *Fzd7* knockdown in WT SuSCs induced cells toward a CS‐like molecular state characterized by coordinated activation of mitophagy‐ and osteogenesis‐linked markers. Specifically, relative to WT + si‐NC, WT + si‐*Fzd7* exhibited increased PINK1, with concordant increases in PARKIN and LC3B‐II together with reduced p62, consistent with engagement of a *Pink1*‐dependent mitophagy program. In the same samples, the osteogenic marker ALPL was also elevated, reaching levels comparable to CS benchmarks, indicating that *Fzd7* reduction is sufficient to induce both mitophagy‐associated and osteogenic protein changes in WT *Prrx1* ^+^ SuSCs (Figure 5C,D). In contrast, *Pink1* co‐silencing reduced the induction of these mitophagy‐associated proteins and attenuated ALPL elevation relative to *Fzd7* knockdown alone, with partial restoration of p62, supporting *Pink1* as a required component for full expression of the *Fzd7*‐loss–associated program.

We next tested whether these marker‐level changes translate into functional osteogenic output. Following osteogenic induction, *Fzd7* knockdown increased mineral deposition as assessed by Alizarin Red S staining and quantification, whereas *Pink1* co‐silencing suppressed this increase and restored mineralization toward control levels (Figure 5E,F). *Pink1* knockdown alone did not phenocopy the *Fzd7* knockdown phenotype in this assay. Together, these data show that *Fzd7* reduction drives WT *Prrx1* ^+^ SuSCs toward a CS‐like state characterized by increased *Pink1*‐dependent mitophagy, and that *Pink1* is required for the full mitophagy and osteogenic response to *Fzd7* loss in vitro.

### 2.5 *Pink1* Is Required for *Fzd7*‐Deficiency–Induced Craniosynostosis in Vivo

**FIGURE 6:**
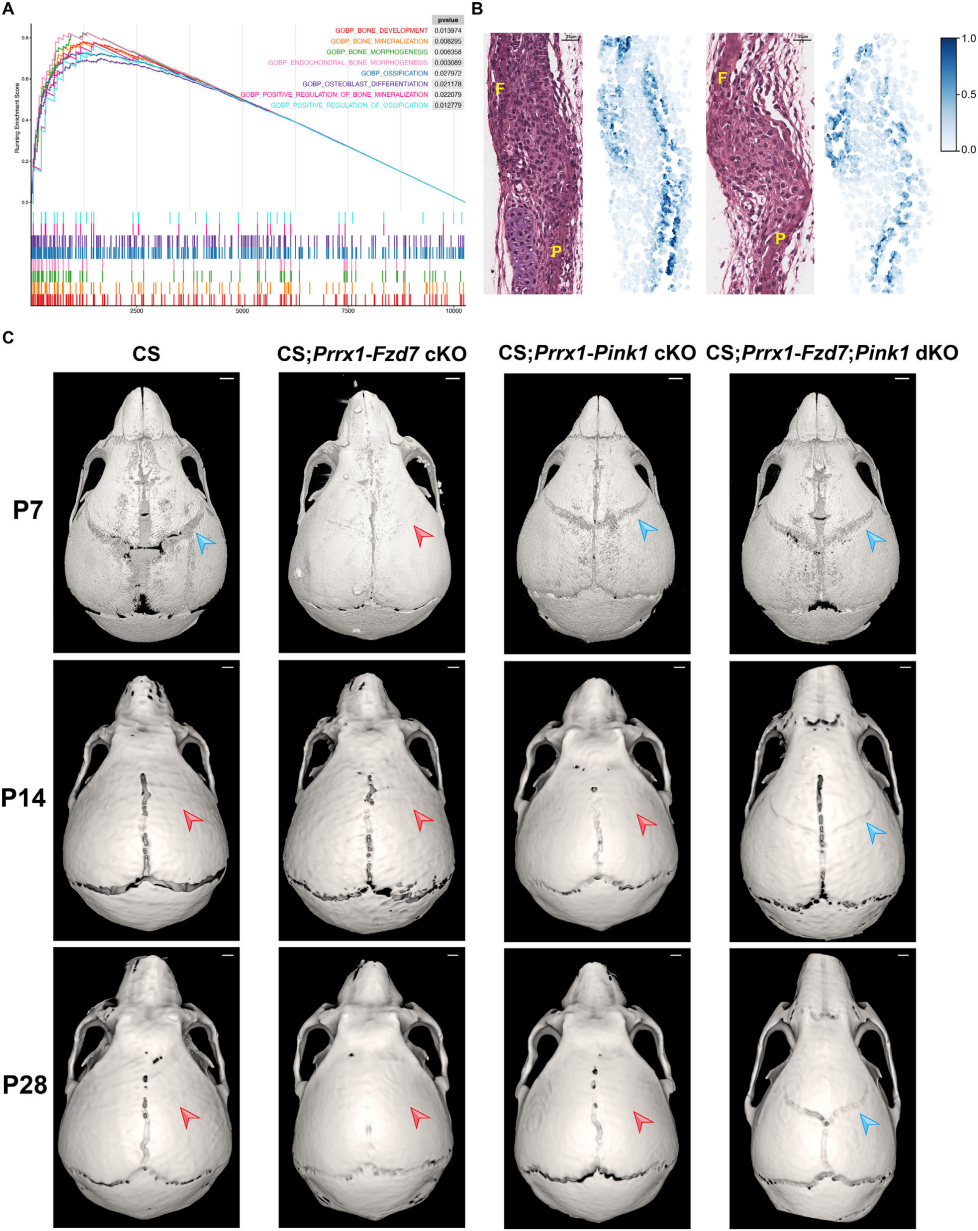
*Pink1* is required for *Fzd7*‐deficiency–associated coronal suture fusion in vivo. (A) GSEA plots showing the significant regulatory perturbation of Gene Ontology Biological Process (GOBP) terms related to bone formation in the ranked gene list of *Pink1* virtual knockout (KO). The ranking is based on the manifold alignment distance, representing the magnitude of regulatory shift caused by the KO. Higher enrichment scores indicate pathways that are most severely affected by *Pink1* depletion (not necessarily upregulation of expression). (B) Spatial visualization of the osteogenic pathway activity (GOBP_OSSIFICATION) on E18.5 CS coronal sutures following *Pink1* virtual knockout. The metagene was constructed using the core enrichment subset identified from GSEA of *Pink1* KO vs WT. F, frontal bone; P, parietal bone. Scale bar: 25 µm. (C) Micro‐CT images of skulls from CS control, single cKO (*Fzd7* or *Pink1*), and *Fzd7*;*Pink1* double KO (dKO) mice at P7, P14, and P28. Red arrowheads indicate coronal suture fusion, whereas blue arrowheads indicate a patent coronal suture. *n* = 5. CS, *Fgfr2^C342Y/+^* craniosynostosis model. Scale bar: 1 mm.

In silico perturbation of *Pink1* in E18.5 CS *Prrx1* ^+^ SuSC regulatory networks produced significant regulatory perturbation of multiple bone formation‐related GOBP terms, including bone morphogenesis, ossification, and osteoblast differentiation (Figure 6A). Although these enriched terms reflect regulatory shifts rather than directionality of gene expression changes, spatial projection of these ossification‐associated leading‐edge metagene further localized predicted pathway activity to regions adjacent to the osteogenic fronts in E18.5 CS coronal sutures (Figure 6B, Figure S5B), indicating a spatial association between *Pink1*‐linked network perturbation and coronal suture ossification activity in CS.

**FIGURE 7:**
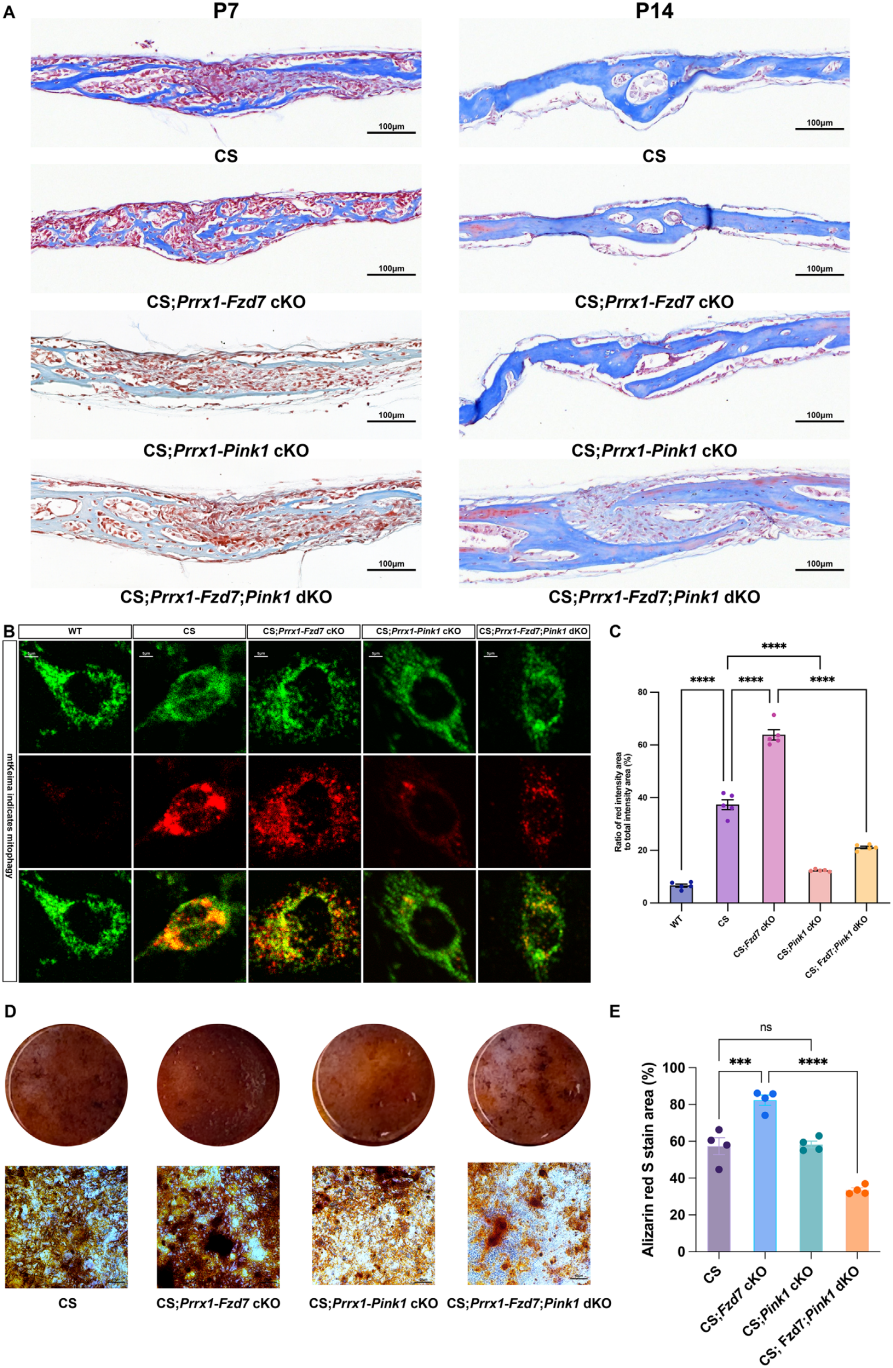
*Pink1* is required for *Fzd7*‐deficiency–induced craniosynostosis in vivo. (A) Masson staining images of coronal sutures from CS control, single cKO (*Fzd7* or *Pink1*), and *Fzd7*;*Pink1* double KO (dKO) mice at P7 and P14. Blue indicates bone, red indicates unmineralized tissue including fibrosis and cells. Scale bar: 100 µm. *n* = 5. (B, C) Visualization of mtKeima in P14 *Prrx1* ^+^ SuSCs. (B) Representative mtKeima images. Green indicates the neutral mitochondrial signal, and red indicates the lysosomal/acidic mtKeima signal. Scale bar, 5 µm. (C) Quantification of the mtKeima mitophagy readout as the ratio of red intensity area to total mtKeima intensity area [red/(red + green)]. *n* = 5. (D, E) Alizarin Red S staining and quantification of calcium deposition in P14 *Prrx1* ^+^ SuSCs from CS control, single cKO (*Fzd7* or *Pink1*), and *Fzd7*; *Pink1* double KO (dKO) mice after 14 days of osteogenic induction. *n* = 3. Significance of all data above was determined by one‐way ANOVA, **p < 0.01, ***p < 0.001, and ****p < 0.0001.

To determine whether *Pink1*‐dependent mitophagy is required for the effects of *Fzd7* loss in vivo, we generated double conditional knockout mice: *Fgfr2^C342Y/+^*; *Fzd7^fl/fl^*; *Pink1^fl/fl^*; *Prrx1^CreERT2/+^* (hereafter CS; *Prrx1*‐*Fzd7*;*Pink1* dKO), alongside single knockouts (CS; *Prrx1‐Fzd7* cKO and CS; *Prrx1‐Pink1* cKO) and CS littermate controls. Micro‐CT analysis showed clear differences in the timing of coronal suture fusion among the genotypes. At P7, coronal sutures were patent in CS controls but showed premature fusion in CS;*Prrx1*‐*Fzd7* cKO mice; this early fusion was absent in the CS; *Prrx1*‐*Fzd7*;*Pink1* dKO. By P14, fusion was evident in CS controls and in both single cKO groups, whereas CS; *Prrx1*‐*Fzd7*;*Pink1* dKO animals retained patency. This divergence persisted through P28, with the dKO remaining patent while the other CS groups were fused (Figure 6C). Masson staining at P7 and P14 provided independent histological support, showing reduced bone/osteoid continuity across the suture midline and better preservation of the midline gap in CS; *Prrx1*‐*Fzd7*;*Pink1* dKO sutures relative to other CS groups (Figure 7A).

We then asked whether the genetic interaction observed at the tissue level is reflected in the target lineage. For these downstream lineage‐based analyses, reporter‐bearing cohorts carried the *Prrx1^CreERT2^*; *R26^tdTomato^* configuration, enabling isolation of *Prrx1*‐lineage suture mesenchymal cells. In FACS‐isolated *Prrx1*‐lineage suture mesenchymal cells from P14 reporter‐bearing cohorts, mtKeima imaging showed elevated lysosomal mtKeima signal in CS compared with WT, with a further increase in CS;*Prrx1*‐*Fzd7* cKO cells (Figure 7B,C). *Pink1* deletion reduced the CS‐associated mtKeima lysosomal shift, and critically, *Pink1* deletion in the *Fzd7*‐deficient background lowered the mtKeima shift relative to CS;*Prrx1*‐*Fzd7* cKO, consistent with *Pink1* dependence of the enhanced mitophagy‐flux readout in this context. Functionally, after 14 days of osteogenic induction ex vivo, *Prrx1* ^+^ SuSCs from CS;*Prrx1*‐*Fzd7* cKO mice exhibited increased mineral deposition by Alizarin Red S staining, whereas the CS; *Prrx1‐Fzd7;Pink1* dKO suppressed this increase. *Pink1* deletion alone did not significantly change mineralization compared with CS controls under these assay conditions (Figure 7D,E).

Together, these in vivo and ex vivo comparisons clarify the phenotype hierarchy across the genetic models: *Fzd7* conditional deletion accelerates coronal suture fusion in the CS background, whereas combined *Fzd7*/*Pink1* deletion attenuates the *Fzd7*‐deficiency‐associated fusion, mitophagy‐flux, and osteogenic phenotypes. In contrast, *Pink1* deletion alone did not recapitulate the *Fzd7*‐loss phenotype in the assays examined. These genetic relationships support *Pink1* as a required component of the full phenotypic response associated with *Fzd7* loss.

## 3 Discussion

In this study, we combined single‐cell RNA sequencing, high‐resolution spatial transcriptomics, and lineage‐focused genetic perturbations to examine how SuSCs contribute to craniosynostosis in the *Fgfr2^C342Y/+^* model. The cellular architecture of the cranial suture niche has been difficult to resolve with conventional transcriptomic approaches because of their limited spatial resolution. By integrating scRNA‐seq with 2‐µm Visium HD spatial transcriptomics and morphology‐guided annotation, we generated a cross‐genotype, multi‐timepoint atlas of the coronal suture. Follow‐up perturbation experiments in vitro and in vivo showed that reduced *Fzd7* expression in *Prrx1* ^+^ SuSCs is accompanied by increased *Pink1*‐dependent mitophagy, enhanced osteogenic activity, and accelerated coronal suture fusion. In the *Fzd7* loss‐of‐function setting, additional immunoblot analyses showed reduced canonical Wnt/β‐catenin‐associated readouts and increased PCP/JNK‐associated readouts, providing pathway‐level context for the consequences of *Fzd7* reduction. The functional and epistasis experiments clarify a phenotype hierarchy across the mutant and restoration models: *Fzd7* loss accelerates coronal suture closure in the *Fgfr2^C342Y/+^* context, local *Fzd7* restoration attenuates this accelerated fusion phenotype, and *Pink1* deletion in the *Fzd7*‐deficient background mitigates the *Fzd7*‐deficiency‐associated fusion and osteogenic phenotypes. These observations support a model in which reduced *Fzd7* engages *Pink1*‐dependent mitophagy as a required component of the full osteogenic and suture‐fusion response in *Prrx1* ^+^ SuSCs.

Previous work has established that cranial sutures contain SuSC populations marked by *Gli1*, *Axin2*, *Ctsk*, and *Prrx1*, with *Prrx1* ^+^ cells representing a major skeletal stem/progenitor pool in both mouse and human sutures [7, 8, 10, 13, 26, 27, 28]. Single‐cell RNA‐seq studies have begun to catalog the heterogeneity of these SuSC and mesenchymal populations in physiologic sutures [12, 14, 17, 29, 30], but most spatial approaches have relied on lower‐resolution, spot‐based platforms that aggregate transcripts from multiple neighboring cell types and thus cannot cleanly separate midline SuSC programs from those of the osteogenic fronts. In parallel, genetic and functional studies have implicated dysregulated Wnt signaling in pathological suture closure and maintenance of suture patency [17, 31, 32, 33], yet these studies have not resolved how and where disease‐associated signaling and SuSC fate programs are engaged over developmental time within the SuSC niche. Recent work has shown that physiological calvarial mineralization and pathological suture fusion can involve interactions between distinct calvarial stem‐cell lineages, including *CTSK* ^+^ and *DDR2* ^+^ populations, rather than the activity of a single stem‐cell compartment alone [34]. Niche‐derived signals can also coordinate SuSC behavior across spatially separated tissue compartments; for example, injury‐induced Cxcl12 and Shh/Ihh signaling has been shown to regulate *Gli1* ^+^ SuSC expansion, directional migration, and osteoblastic differentiation during calvarial bone regeneration [35]. In pathological settings, altered progenitor‐cell states have likewise been implicated in suture fusion. Our recent work revealed that p38α MAPK‐induced senescence in cranial suture progenitor cells promotes osteogenic differentiation, progenitor‐cell exhaustion, and craniosynostosis‐associated phenotypes [36]. These studies indicate that cranial suture closure could be viewed as an outcome of altered stem/progenitor composition, niche signaling, and lineage output, rather than as a consequence of a single marker‐defined cell population. The original *Fgfr2^C342Y/+^* model showed that coronal suture abnormalities are already detectable during embryogenesis, with increased frontal‐parietal bone overlap at E16.5 and coronal suture obliteration by P29 [16]. In our previous study, we further resolved this intervening window, showing that by P7 the coronal suture already exhibits mesenchymal depletion, structural disorganization, and trabecular bridging, followed by complete closure at P14–P28 [36]. We therefore focused the discovery atlas on E14.5, E18.5, and P3 to capture early disease‐associated SuSC states. Later postnatal stages were used to assess tissue‐level fusion phenotypes, where the interpretation of single‐cell and spatial profiles would be more strongly influenced by fusion‐associated remodeling and changes in tissue composition. By integrating scRNA‐seq with 2‐µm‐resolution Visium HD spatial transcriptomics across E14.5, E18.5, and P3 and using near‐single‐cell spatial annotation to localize *Prrx1* ^+^ SuSCs in *Fgfr2^C342Y/+^* and WT sutures, our analysis identifies a niche‐restricted state in the craniosynostotic sutures characterized by reduced *Fzd7* expression, increased *Pink1*‐dependent mitophagy signatures and flux readouts, and early osteogenic priming.

Mitophagy and Wnt signaling have both been implicated in skeletal biology, but their intersection in suture stem cells, particularly in craniosynostosis, remains poorly defined. Canonical Wnt/β‐catenin signaling is a crucial regulator of mesenchymal stem cell osteogenesis and cranial suture fate, and altered Wnt activity has been linked to craniosynostosis and disordered suture maintenance in both mouse models and human studies [5, 17, 37, 38]. Frizzled receptors, including *Fzd7*, mediate Wnt ligand responses and can help maintain stem or progenitor cell states in other tissues; thus, changes in *Fzd7* expression have the potential to reshape progenitor behavior [20, 39, 40]. In the *Fgfr2^C342Y/+^* model, constitutive FGFR2 activation may also contribute to the WNT‐suppressed state observed in CS *Prrx1* ^+^ SuSCs. Previous studies have shown that FGF and WNT signaling are closely coordinated during skeletal progenitor fate regulation and cranial suture development [32, 41]. Recently, FGFR2 signaling has been shown to regulate local WNT activity during skull development through induction of the WNT inhibitor Wif1, supporting the concept that altered FGFR2 activity can reshape the WNT signaling environment in cranial tissues [41]. In this context, the reduced expression of Frizzled receptors in CS *Prrx1* ^+^ SuSCs may reflect one aspect of altered FGF‐WNT pathway coordination downstream of mutant FGFR2. Among the WNT receptors examined, *Fzd7* was prioritized because it showed both reduced expression in CS *Prrx1* ^+^ SuSCs and a genotype‐dependent reversal in its spatial expression trend relative to the *Prrx1* ^+^ SuSC domain. Together with prior evidence that WNT signaling supports cranial suture skeletal progenitor maintenance and that FZD7 can serve as a functionally relevant WNT receptor in stem/progenitor cell states [17, 42], these findings place *Fzd7* downregulation within the broader *Fgfr2*‐driven signaling context. Consistent with this interpretation, *Fzd7* silencing in WT *Prrx1* ^+^ SuSCs reduced active β‐catenin, AXIN2, and Cyclin D1, while increasing p‐JNK and VANGL1. These results suggest that *Fzd7* loss is accompanied by a shift in Wnt pathway‐associated protein readouts, with reduced canonical Wnt/β‐catenin‐associated outputs and increased PCP/JNK‐associated readouts. However, the direct molecular intermediates linking mutant FGFR2 activity to reduced *Fzd7* expression remain to be defined. Moreover, these immunoblot‐based readouts do not establish whether the canonical or PCP/JNK branch directly mediates the *Pink1*‐associated mitophagy phenotype. Branch‐specific Wnt perturbation and rescue experiments will be needed to define the upstream signaling intermediates connecting FZD7 to *Pink1*‐dependent mitophagy.

Mitophagy is widely viewed as a mitochondrial quality‐control mechanism that removes damaged organelles and supports bone homeostasis, and *Pink1*‐dependent mitophagy has been shown to influence osteoblast differentiation and osteoporosis‐related bone loss [43, 44]. However, reviews of bone metabolism increasingly emphasize that abnormal levels of autophagy and mitophagy—either insufficient or excessive—disrupt the balance between bone formation and resorption and contribute to bone metabolic disorders [45, 46]. Mitochondrial quality‐control systems, including mitochondrial dynamics, mitophagy, mitochondrial biogenesis, and redox regulation, are increasingly recognized as regulators of bone‐related cell differentiation and bone homeostasis [47]. Within this broader framework, *Pink1*‐dependent mitophagy is generally viewed as a homeostatic process that removes dysfunctional mitochondria and helps maintain cellular fitness. Importantly, recent lineage‐based evidence indicates that *Pink1* has functions in mesenchymal progenitor osteogenesis beyond being a general stress‐response marker: conditional deletion of *Pink1* in Prx1‐lineage mesenchymal cells reduced trabecular and cortical bone mass and suppressed MSC osteogenic differentiation, at least in part through altered Apoh transcription [48]. Together with previous work linking *Pink1*‐dependent mitophagy to MSC osteogenesis and new bone formation, these studies support the concept that *Pink1*‐related mitochondrial quality‐control pathways can influence skeletal progenitor fate. In this setting, our data link reduced *Fzd7* signaling in *Prrx1* ^+^ SuSCs to altered mitophagy. In the present study, reduced *Fzd7* in *Prrx1^+^* SuSCs is associated with increased *Pink1*‐dependent mitophagy and enhanced osteogenic activity, and *Pink1* loss in vitro and in vivo attenuates these *Fzd7*‐deficiency–associated phenotypes. These findings suggest that, rather than mitophagy simply restoring mitochondrial fitness, *Fzd7* downregulation in SuSCs is linked to a *Pink1*‐dependent effector component that contributes to osteogenic activation. These findings extend previous Wnt‐centered models of cranial suture biology by linking reduced *Fzd7* expression in SuSCs to mitophagy‐associated changes and increased osteogenic output in this disease model. We also acknowledge that increased mitophagy should not be interpreted as intrinsically pathogenic, as mitophagy is commonly involved in mitochondrial quality control. To address whether the enhanced mitophagy‐associated readouts reflected mitochondrial stress, we added ROS and JC‐1 assays. Under the conditions tested, CS‐derived *Prrx1* ^+^ SuSCs and WT *Prrx1* ^+^ SuSCs after *Fzd7* knockdown did not show significant ROS accumulation or mitochondrial membrane potential loss, whereas the corresponding positive‐control treatments produced the expected assay responses. These results argue against ROS elevation or mitochondrial depolarization as the primary drivers for the increased mitophagy‐associated readouts. However, we cannot exclude the possibility of pathway‐specific mitochondrial functional alterations, as comprehensive analyses of respiratory capacity, substrate utilization, and time‐resolved mitochondrial dynamics were not performed in this study. We therefore interpret *Pink1*‐dependent mitophagy as a required functional component of the *Fzd7*‐loss–associated osteogenic program, rather than as evidence that increased mitophagy is intrinsically pathogenic or necessarily the primary initiating event.

However, our data identify *Pink1* as an important downstream requirement for the *Fzd7*‐deficiency phenotype in SuSCs rather than establishing a fully linear pathway from *Fzd7* to *Pink1*. Although the added Wnt pathway‐associated protein analyses show that *Fzd7* silencing reduces canonical Wnt/β‐catenin‐associated readouts and increases PCP/JNK‐associated readouts, these data should be interpreted as pathway‐level context rather than as proof of a branch‐specific Wnt‐to‐*Pink1* signaling cascade. The in vitro and in vivo epistasis experiments indicate that *Pink1* is required for the full expression of the mitophagy and osteogenic phenotypes we observe when *Fzd7* is reduced, but they do not exclude additional *Pink1*‐independent routes through which *Fzd7* loss and *FGFR2* gain‐of‐function signaling may perturb mitochondrial homeostasis and SuSC fate. Consistent with this, *Pink1* deletion alone in the CS background did not fully normalize SuSC mitophagy and osteogenic readouts toward WT levels in our in vivo and ex vivo assays, indicating that *Pink1*‐dependent mitophagy is an important but not exclusive contributor to the disease‐associated state. We also have not yet identified the molecular intermediates linking altered Wnt receptor status to changes in *Pink1* expression or activity, and it remains possible that *Fzd7* influences SuSC behavior through both mitochondrial and non‐mitochondrial mechanisms. In addition, the genetic tools used here label *Prrx1*‐expressing suture skeletal stem and progenitor cells, which are widely used to define SuSCs in the calvarial niche [6, 10], but they do not resolve potential heterogeneity within the *Prrx1^+^* compartment. Thus, our in vivo conclusions apply to the *Prrx1^+^* SuSC pool as a whole and may encompass effects on closely related progenitor states within this lineage. The AAV‐*Fzd7* rescue approach provides local but not SuSC‐specific modulation. Although western blotting confirmed FZD7 protein upregulation in the injected coronal suture region 48 h after P0 AAV‐*Fzd7* delivery, AAV9‐mediated perisutural delivery is not cell‐type specific and may transduce multiple sutural niche populations. Accordingly, the rescue phenotype should be interpreted as local restoration of FZD7 within the suture rather than SuSC‐specific rescue. The observed improvement may involve *Prrx1* ^+^ SuSCs together with neighboring osteoblasts, fibroblasts, and other suture‐associated cells. The agreement between tissue‐level rescue and partial normalization measured in FACS‐isolated *Prrx1* ^+^ SuSCs supports, but does not prove, a SuSC‐intrinsic component of the rescue effect. Finally, the causal tests presented here are restricted to an *Fgfr2^C342Y/+^* model, a defined set of developmental timepoints, and the coronal suture, and additional work will be needed to determine how broadly the *Fzd7*–*Pink1*–mitophagy axis operates across other sutures, genetic backgrounds, and human disease contexts. Overall, our findings indicate that *Pink1*‐dependent mitophagy is required for the full *Fzd7*‐loss phenotype in *Prrx1* ^+^ SuSCs in the *Fgfr2^C342Y/+^* model. Additional work will be needed to define the upstream intermediates, the relevant cell states within the *Prrx1* lineage, and the generality of this mechanism across other suture contexts.

## 4 Materials and Methods

### 4.1 Animals

All animal experiments were conducted in accordance with institutional and national guidelines for the care and use of laboratory animals and were approved by the Ethics Committee of the Chinese Academy of Medical Sciences and Peking Union Medical College (Approval No. 2023‐A‐79; Date of approval: 28 August 2023). Title of the approved project: Endoplasmic reticulum mediated apoptosis of cranial suture mesenchymal stem cells and inflammation in the molecular mechanisms underlying craniosynostosis.

Mice were maintained on a C57BL/6J background in a specific pathogen–free facility under a 12 h light/12 h dark cycle at 20°C–22°C and 30%–70% relative humidity, with ad libitum access to standard chow and water. Both male and female animals were used for experiments. Littermates were used as controls whenever possible. Animals were monitored daily for general health and body condition; no unexpected morbidity or mortality attributable to experimental procedures was observed.

The *Fgfr2^C342Y/+^* model was generated by CRISPR/Cas9‐mediated introduction of a Cys361Tyr point mutation into the mouse *Fgfr2*‐215 transcript (ENSMUST00000122054.8), corresponding to the human Cys342Tyr variant [15, 16], by Collective Pharmachem Biotechnology Co., Ltd. (Nanjing, China). The Cys361Tyr designation reflects the amino acid position in the mouse *Fgfr2*‐215 reference transcript, whereas *Fgfr2^C342Y/+^* is used throughout the manuscript to denote the orthologous mutation corresponding to the human *FGFR2* C342Y variant associated with Crouzon syndrome. Prior work validated the phenotype of this CRISPR‐based line through micro‐CT, skeletal staining, and histology [15]. Founder animals carrying the desired mutation were identified by Sanger sequencing of PCR amplicons from genomic DNA and backcrossed to C57BL/6J to establish a stable colony. For timed pregnancies, *Fgfr2^C342Y/+^* males were mated with C57BL/6J females overnight; the morning a vaginal plug was detected was designated embryonic day 0.5 (E0.5). Embryos were harvested at E14.5 and E18.5, and postnatal pups were collected at P3, P7, P14, and P28 as indicated in the figures.

Embryos were collected at E14.5 and E18.5 following CO_2_ euthanasia of pregnant dams (20%–30% chamber volume/min), immediately followed by laparotomy and rapid embryo decapitation. Postnatal pups were euthanized by CO_2_ inhalation (20%–30% chamber volume/min) until loss of righting reflex and cessation of respiration, followed by cervical dislocation as a secondary physical method. No anesthesia was administered to animals in this study. All embryonic and postnatal tissue collections, as well as subsequent imaging procedures, were carried out only after euthanasia.

#### 4.1.1 Breeding Strategy

##### 4.1.1.1 Mouse Lines and Allele Nomenclature

The mouse lines used for breeding included the compound *Fzd7^fl/+^*; *Prrx1^CreERT2/+^* line, *Fzd7^fl/+^*, *Prrx1^EGFP/+^*, *R26^tdTomato/+^*, *Prrx1^CreERT2/+^*, and *Pink1^fl/+^* mice, which were obtained from the Shanghai Model Organisms Center (Shanghai, China). The *Prrx1*‐2A‐CreERT2 allele is a knock‐in allele generated at the *Prrx1* termination codon, whereas the *Prrx1*‐EGFP reporter allele was generated by targeted insertion of EGFP at the *Prrx1* start‐codon locus. The *Rosa26* reporter line carries a CAG‐LSL‐tdTomato‐WPRE‐polyA cassette targeted to the *Rosa26* locus, and the *Pink1* conditional allele contains loxP sites flanking exons 2–3. Because *Fzd7* and *Prrx1* are both located on mouse chromosome 1, these two alleles cannot be readily combined through conventional breeding of independently inherited lines. Therefore, a *Prrx1*‐2A‐CreERT2 knock‐in strategy was implemented directly on an *Fzd7*‐floxed background through targeted genome engineering, rather than relying on meiotic recombination between separate *Fzd7*‐floxed and *Prrx1*‐CreERT2 alleles. This generated the linked *Fzd7^fl/+^*; *Prrx1^CreERT2/+^* allele configuration used for subsequent breeding. The resulting line was further crossed to obtain *Fzd7^fl/fl^*; *Prrx1^CreERT2/+^* mice, enabling tamoxifen‐inducible deletion of *Fzd7* specifically in *Prrx1*‐lineage cells (Figure S4B). The allele generation strategy is illustrated in Figure S6A.

Abbreviated genotype labels used throughout the manuscript are summarized in Table S2.

##### 4.1.1.2 Basic CS and Reporter Cohorts

To generate the basic craniosynostosis cohort, male *Fgfr2^C342Y/+^* mice were crossed with female wild‐type C57BL/6J mice to obtain *Fgfr2^C342Y/+^* and wild‐type littermates. To generate the core craniosynostosis reporter cohort used for reporter‐based isolation of *Prrx1* ^+^ suture mesenchymal cells, male *Fgfr2^C342Y/+^* mice were crossed with female *Prrx1^EGFP/+^* mice to obtain *Fgfr2^C342Y/+^*; *Prrx1^EGFP/+^* offspring (Figure S4A).

##### 4.1.1.3 Intermediate Breeder Cohorts

To improve target‐genotype yield for subsequent breeding, intermediate breeder cohorts were expanded as outlined in Figure S4B. These included maintenance or generation of *Fzd7^fl/fl^*; *Prrx1^CreERT2/+^*, *Pink1^fl/fl^*; *Prrx1^CreERT2/+^*, *Pink1^fl/fl^*; *R26^tdTomato/+^*, and related breeder lines used for conditional knockout and reporter crosses.

##### 4.1.1.4 *Fzd7* Conditional Knockout Cohorts

For inducible deletion of *Fzd7* in the *Prrx1* lineage, male *Fgfr2^C342Y/+^* mice were first crossed with female *Fzd7^fl/fl^*; *Prrx1^CreERT2/+^* mice to generate *Fgfr2^C342Y/+^*; *Fzd7^fl/+^*; *Prrx1^CreERT2/+^* offspring. These mice were then crossed again with *Fzd7^fl/fl^*; *Prrx1^CreERT2/+^* breeders to obtain *Fgfr2^C342Y/+^*; *Fzd7^fl/fl^*; *Prrx1^CreERT2/+^* experimental mice and related control cohorts (Figure S4C). For reporter‐bearing *Fzd7* conditional knockout cohorts, *Fzd7^fl/fl^*; *Prrx1^CreERT2/+^* mice were crossed with *R26^tdTomato/+^* mice to generate *Fzd7^fl/fl^*; *Prrx1^CreERT2/+^*; *R26^tdTomato/+^* breeders, which were then crossed with *Fgfr2^C342Y/+^*; *Fzd7^fl/fl^*; *Prrx1^CreERT2/+^* mice to obtain *Fgfr2^C342Y/+^*; *Fzd7^fl/fl^*; *Prrx1^CreERT2/+^*; *R26^tdTomato/+^* offspring.

##### 4.1.1.5 *Pink1* Conditional Knockout Cohorts

For inducible deletion of *Pink1* in the same lineage, male *Fgfr2^C342Y/+^* mice were crossed with female *Pink1^fl/fl^*; *Prrx1^CreERT2/+^* mice to generate *Fgfr2^C342Y/+^*; *Pink1^fl/+^*; *Prrx1^CreERT2/+^* offspring, which were then crossed with *Pink1^fl/fl^* breeders to obtain *Fgfr2^C342Y/+^*; *Pink1^fl/fl^*; *Prrx1^CreERT2/+^* mice (Figure S4D). For reporter‐bearing *Pink1* conditional knockout cohorts, *Pink1^fl/fl^* mice were crossed with *R26^tdTomato/+^* mice to generate *Pink1^fl/fl^*; *R26^tdTomato/+^* breeders, which were then crossed with *Fgfr2^C342Y/+^*; *Pink1^fl/fl^*; *Prrx1^CreERT2/+^* mice to obtain *Fgfr2^C342Y/+^*; *Pink1^fl/fl^*; *Prrx1^CreERT2/+^*; *R26^tdTomato/+^* offspring.

##### 4.1.1.6 *Fzd7*/*Pink1* Double Conditional Knockout Cohorts

For generation of *Fzd7*/*Pink1* double conditional knockout cohorts, *Pink1^fl/fl^* mice were crossed with *Fzd7^fl/fl^*; *Prrx1^CreERT2/+^* mice to generate compound intermediate breeders, which were further intercrossed as shown in Figure S4D to obtain *Fzd7^fl/fl^*; *Pink1^fl/fl^*; *Prrx1^CreERT2/+^* breeders and, subsequently, *Fgfr2^C342Y/+^*; *Fzd7^fl/fl^*; *Pink1^fl/fl^*; *Prrx1^CreERT2/+^* experimental mice. Reporter‐bearing double conditional knockout cohorts were generated by introducing *R26^tdTomato/+^* through the same compound breeding scheme to obtain *Fgfr2^C342Y/+^*; *Fzd7^fl/fl^*; *Pink1^fl/fl^*; *Prrx1^CreERT2/+^*; *R26^tdTomato/+^* mice.

##### 4.1.1.7 Breeding‐Schematic Notes, Tamoxifen Induction, and Genotyping

Where indicated in the breeding schematic, breeder sex was specified for selected crosses; some intermediate colony‐expansion or self‐crossing steps used to improve target‐genotype yield are not shown. Pregnant dams carrying *Prrx1* ^CreERT2^‐positive embryos were administered tamoxifen (T5648, Sigma–Aldrich, St. Louis, MO) in corn oil at 15 mg/mL by intraperitoneal injection once daily for 3 consecutive days starting at E14.5. Genotyping was performed using genomic DNA extracted from tail biopsies or embryonic tissue. Primer sequences used for genotyping are provided in Table S3.

### 4.2 AAV Injection

AAV‐mediated *Fzd7* overexpression was performed using an AAV9 vector carrying mouse *Fzd7* under the control of the CAG promoter (pAAV‐CAG‐Fzd7‐3xFLAG‐tWPA; OBiO Technology, Shanghai, China). The corresponding empty AAV9 vector, pAAV‐CAG‐MCS‐tWPA (backbone GL3037; OBiO Technology), was used as a control. Newborn mice were injected perisuturally at P0 (within 12 h after birth) in the coronal suture region. A total volume of 5 µL virus suspension was delivered per animal at a titer of 5 × 10^1^ ^2^ vector genomes (vg)/mL. No general anesthesia was used for this neonatal injection procedure. Injections were performed rapidly under manual restraint, and pups were returned to warming and then to the dam immediately after the procedure, with post‐procedure monitoring for recovery and general condition. The procedure was performed in accordance with the approved animal protocol (Approval No. 2023‐A‐79). Mice were collected at the indicated time points for histological and micro‐CT analyses.

### 4.3 Coronal Suture Dissociations

Coronal sutures were collected from *Fgfr2^C342Y/+^* and wild‐type littermates at embryonic days E14.5, E18.5 and postnatal days P3, P7, P14 and P28. For embryonic stages, skull caps were removed in ice‐cold phosphate‐buffered saline (PBS) under a stereomicroscope. The frontal and parietal bones were gently separated along the suture line, and the coronal suture region was microdissected. For postnatal stages, skulls were cleaned of soft tissue, and coronal sutures were isolated by careful removal of overlying skin and periosteum followed by separation of the frontal and parietal bones. The dissected coronal suture‐region tissue included the suture mesenchyme, immediately adjacent frontal and parietal bone fronts, and associated dura mater. Overlying skin and unrelated external soft tissues were removed during dissection.

For experiments requiring single‐cell suspensions, coronal sutures from three animals of the same genotype and age were pooled per biological replicate to ensure sufficient cell numbers.

For scRNA‐seq, the frontal and parietal bones were gently separated to expose the coronal suture, and tissue from five sutures was pooled to generate each library, with male and female samples processed together.

For HD ST‐seq, coronal sutures were fixed in 4% paraformaldehyde in PBS at room temperature for 12–24 h, followed by dehydration in a graded ethanol series (70%, 95%, 100%; 30 min each) and two clearing steps in xylene (30 min each). Tissues were then infiltrated with molten paraffin at 58°C under vacuum in two 30‐min cycles, transferred into molds containing fresh paraffin, and cooled on a chilled surface to harden. The resulting FFPE blocks were kept at room temperature until sectioning. For exact localization of the coronal suture at E14.5, sections were cut transversely parallel to a plane defined by the pupil and outer‐ear midpoint, ∼1 mm superior to eye level.

### 4.4 scRNA‐Seq Library Preparation

Single‐cell RNA‐seq libraries were prepared from two biological replicates per genotype (CS and WT) at E14.5 and E18.5, and three biological replicates per genotype at P3. Each biological replicate consisted of five pooled cranial suture tissues. Single‐cell suspensions were loaded together with gel beads carrying cell barcodes and unique molecular identifiers (UMIs) at a limiting dilution, such that individual cells were captured in separate Gel Bead‐in‐Emulsion (GEM) droplets. After exposure to lysis buffer, polyadenylated mRNA hybridized to the oligonucleotides on the gel beads. The beads were then collected into a single tube for reverse transcription, during which each mRNA molecule was converted to cDNA and labeled at its 5′ end (corresponding to the original 3′ end of the mRNA) with a cell‐specific barcode and UMI. The bead‐associated cDNA was subsequently subjected to second‐strand synthesis, adaptor ligation, and amplification following the manufacturer's protocol (10x Genomics, CG000206 Rev D). Libraries were constructed to enrich for 3′ transcript ends carrying barcodes and UMIs. Library integrity was evaluated on an Agilent 2100 Bioanalyzer using High Sensitivity DNA chips, and concentrations were determined with the Qubit High Sensitivity DNA assay (Thermo Fisher Scientific). Sequencing was performed on an Illumina NovaSeq 6000 platform using 2 × 150 bp paired‐end reads. Across all 14 libraries, the mean sequencing depth was 28255 ± 6964 reads per cell (17569–41318).

### 4.5 Spatial Transcriptomics Library Preparation (FFPE‐HD)

Spatial transcriptomic profiling was carried out on coronal sutures from *Fgfr2^C342Y/+^* and wild‐type C57BL/6 mice at E14.5, E18.5, and P3, with two CS and one WT replicate per stage. Formalin‐fixed paraffin‐embedded (FFPE) sections that met RNA quality criteria (DV200> 30%) were processed using the 10x Genomics Visium HD Spatial Gene Expression platform for FFPE tissue. For each developmental time point, 5 µm coronal suture sections from two CS and one WT sample were placed on the same Visium HD slide (10x Genomics) in a 2CS+1WT arrangement, baked at 42°C for 3 h, and then left to dry overnight in a desiccator at room temperature.

Slides were deparaffinized by incubation at 60°C for 2 h, followed by xylene treatment and rehydration through a graded ethanol series (70%, 95%, 100%). Hematoxylin and eosin (H&E) staining was performed using Mayer's hematoxylin (Millipore Sigma), Bluing Reagent (Dako, Agilent), and alcoholic eosin (Millipore Sigma). Stained slides were scanned to record tissue morphology. RNA crosslinks were then reversed by incubating the sections in 0.1 N HCl and subsequently in TE buffer (pH 9.0).

Next, sections were hybridized with the Mouse Whole Transcriptome Probe Panel (10x Genomics). Each probe pair consisted of a 5′ oligonucleotide carrying the Small RNA Read 2S sequence and a 3′ oligonucleotide with a poly‐A tail. After hybridization to target RNAs, probe pairs were ligated to form single‐stranded ligation products. Samples were treated with RNase and permeabilized to release these ligation products, which were then captured by poly(dT) probes immobilized on the Visium slide. These capture probes contain sequences for Illumina Read 1, spatial barcodes, and unique molecular identifiers (UMIs). Captured ligation products were extended to generate spatially barcoded molecules, released from the slide, and subjected to sample indexing PCR and final library construction. The resulting Visium HD libraries, flanked by P5 and P7 adapters, were sequenced on an Illumina NovaSeq 6000 system using paired‐end mode, with Read 1 comprising a 16 bp spatial barcode and 12 bp UMI, and Read 2S reading the ligated probe inserts, at a target depth of approximately 50 000 reads per spot.

### 4.6 Single‐Cell Sequencing Data Processing

Single‐cell RNA sequencing data were aligned to a customized mouse reference genome (GRCm38 for E14.5 and E18.5, GRCm39 for P3) using CellRanger. The raw gene expression matrix contained 157 954 cells and 27 468 genes. Data normalization was conducted using SCTransform [49], which simultaneously performs normalization, variance stabilization, and identifies 3000 highly variable genes per sample. Batch effect correction across samples was achieved using Harmony integration [50]. 30 principal components were used for subsequent dimensional reduction and clustering analysis. Cell cycle phase assignment was performed using established S‐phase and G2/M‐phase gene signatures. Doublet detection was implemented using scDblFinder to identify potential cell doublets based on transcriptomic profiles. Dimensionality reduction for visualization employed both t‐distributed stochastic neighbor embedding (t‐SNE) and Uniform Manifold Approximation and Projection (UMAP) based on the Harmony‐corrected embeddings. Unsupervised clustering was performed using the Louvain algorithm with a resolution of 0.8, resulting in 35 distinct clusters. Differential gene expression analysis was conducted using the Wilcoxon rank‐sum test through FindAllMarkers function [51] on the SCT‐normalized data after PrepSCTFindMarkers preprocessing. Significantly enriched genes were defined as those with adjusted p‐value < 0.05, log fold change > 0.25, and expression in at least 10% of cells within the cluster. Marker genes were ranked by pct_diff (the absolute difference between pct.1 and pct.2) in descending order, followed by avg_log2FC. The cluster identified as erythroid progenitor cells—characterized by high expression of *Car2*, *Slc25a21*, *Ermap*, *AI662270*, and *Mt2*—was excluded (Figure S1A–D, Data S2).

Based on prior work [6, 12], we re‐clustered suture mesenchymal cells, focusing on *Col1a1^+^* populations that also expressed at least one osteogenic marker (*Sp7*, *Runx2*, or *Alpl*). Suture mesenchymal cells followed the same pipeline used for normalization, variable feature selection, batch correction, dimensionality reduction, and unsupervised clustering, with the clustering resolution set to 0.5. Using established marker genes [8], we then classified the resulting osteogenic and mesenchymal clusters as 10 subpopulations corresponding to 9 major cell types (Figure 1B, Figure S3A,B): SuSCs, characterized by expression of canonical mesenchymal stem cell markers (*Nt5e^+^*, *Thy1^+^*, *Eng^+^*) and lack of lineage‐specific differentiation genes (*Sp7* or *Ifitm5*). After excluding known cell types from previous in situ hybridization studies, *Prrx1^+^* SuSCs were defined as cells within the SuSC compartment that expressed *Prrx1* (*Tnn* ^+^, *Bcl11b* ^+^, *Sgcd* ^+^, *Rbms3* ^+^, *Rora* ^+^); Proliferating preosteoblast (*Top2a* ^+^, *Birc5* ^+^, *Hmmr* ^+^, *Tpx2* ^+^, *Ube2c* ^+^); *Mmp13* ^+^‐preosteoblast (*Mmp13* ^+^, *Cfh* ^+^, *Serpine2* ^+^, *Cp* ^+^, *Fap* ^+^); Pre‐osteoblast (*Alpl* ^+^, *Npnt* ^+^, *Acta2* ^+^, *Pdzrn4* ^+^, *Prrx2* ^+^); Immature osteoblast (*Ibsp* ^+^, *Cd200* ^+^, *Tcf7* ^+^, *Col22a1* ^+^, *Sgms2* ^+^); Mature osteoblast (*Spp1* ^+^, *Lyz2* ^+^, *S100a9* ^+^, *Lgals3* ^+^, *Tyrobp* ^+^); Dura fibroblast including Outer dura fibroblast (*Pdgfrl* ^+^, *Fmod* ^+^, *Matn4* ^+^, *Col14a1* ^+^, *Tenm3* ^+^) and Inner dura fibroblast (*Crabp2* ^+^, *Nppc* ^+^, *Slc5a6* ^+^, *Ntrk3* ^+^, *Slc16a9* ^+^); Ectocranial suture mesenchyme (*Clec3b* ^+^, *Dpp4* ^+^, *Ly6a* ^+^, *Pi16* ^+^, *Dpt* ^+^); Ligament‐like mesenchyme (*Tnmd* ^+^, *Erbb4* ^+^, *Alcam* ^+^, *Pde3a* ^+^, *Sfrp2* ^+^).

### 4.7 HD Spatial Sequencing Data Analysis

After sequencing, reads were aligned to the Visium Mouse Transcriptome Probe Set (v2.0), and expression matrices were generated with the Space Ranger pipeline. Bins with fewer than 10 total counts were excluded, as were genes detected in fewer than 2 cells. Regions of interest (ROIs) were defined in Loupe Browser (v8.1.2) and used for downstream spatial analysis. Gene expression within each ROI was normalized by total‐count scaling followed by log transformation.

### 4.8 Annotation of Visium HD Spatial Transcriptomic Data

Visium HD provides 2‐µm‐resolution spatial transcriptomic profiles at subcellular scale. To enable near‐single‐cell annotation, we reconstructed approximate cell‐scale transcriptional units from bin‐level measurements [52]. To achieve this, H&E‐stained Visium HD sections were first subjected to nuclear segmentation using a StarDist‐based approach [53] implemented in QuPath (v0.5.1) [54], allowing image‐derived nuclear territories to be identified on the matched histology image. The resulting segmentation masks were then mapped back to the Visium HD coordinate system, and neighboring 2‐µm bins were aggregated into cell‐scale territories following the general logic of Bin2cell (v0.3.3), which reconstructs approximate single‐cell transcriptional units from high‐resolution Visium HD data [55, 56]. This step reduces the ambiguity inherent in bin‐level measurements and enables transcript counts to be summarized at the level of segmented cellular regions while preserving spatial position.

To assign biological identities to the reconstructed spatial units, we next applied the TopACT framework [57], which was developed for cell‐type classification in subcellular‐resolution spatial transcriptomic data. TopACT uses a reference‐based machine‐learning strategy to transfer cell‐type labels from matched single‐cell RNA‐seq data to high‐resolution spatial transcriptomic profiles, thereby enabling classification of reconstructed spatial units in tissue context. In this study, stage‐matched scRNA‐seq datasets were used as references for Visium HD annotation at E14.5, E18.5, and P3.

We developed SpatialCell (GitHub: https://github.com/Xinyan‐C/SpatialCell) as an open‐source pipeline to integrate these steps into a unified workflow for Visium HD analysis, including histology‐guided segmentation, bin‐to‐cell aggregation, reference‐based cell‐type annotation, and downstream spatial visualization. In the present study, SpatialCell was used to generate near‐single‐cell spatial maps of the coronal suture and to localize *Prrx1* ^+^ SuSCs and neighboring niche populations across developmental stages and genotypes.

The validity of this annotation strategy was evaluated by orthogonal comparison between SpatialCell‐derived cell‐type maps and independent *Prrx1* immunofluorescence staining on matched coronal suture sections, which showed concordant localization of *Prrx1* ^+^ cells with the annotated *Prrx1* ^+^ SuSC domain. In addition, the resulting spatial maps were consistent with known tissue architecture, established marker‐gene distributions, and published in situ and single‐cell descriptions of murine coronal suture organization [12, 30] (Figure S2B–D).

### 4.9 Gene Ontology Analysis

Gene ontology (GO) analysis between CS and WT was performed separately at E14.5, E18.5, and P3. For each time point, cells were grouped by genotype, normalized using Seurat (v5.2.0), and analyzed with the FindMarkers function using the MAST test. Genes with adjusted *p* values < 0.05 were considered significant, and genes with average log2 fold change (avg_log2FC) > 0.25 or < ‐0.25 were defined as significantly upregulated or downregulated, respectively. Upregulated and downregulated gene sets were then analyzed separately for Gene Ontology Biological Process (BP) enrichment using the enrichGO function in clusterProfiler with org.Mm.eg.db as the annotation database and gene symbols as input. Multiple‐testing correction was performed using the Benjamini–Hochberg method. GO terms with adjusted *p* values < 0.05, q values < 0.05, and more than five overlapping genes were considered significantly enriched.

### 4.10 Spatial Metagene Analysis

Metagene scores were computed with the Banksy [25] framework by summing functionally related genes into composite signatures and z‐score normalizing for cross‐sample comparison. For pathway‐based analyses, gene sets were first derived from enriched GSEA terms by extracting the core_enrichment genes from related GO Biological Process pathways at each time point. These gene sets were then projected onto Visium HD spatial data, Metagene scores were visualized on the spatial coordinate system of the corresponding sample and ROI to assess regional enrichment patterns within the coronal suture.

### 4.11 scTenifoldKnk‐Based in Silico Perturbation Analysis

In silico gene perturbation analysis was performed using scTenifoldKnk [23] on the E18.5 *Prrx1* ^+^ SuSC subset extracted from the integrated scRNA‐seq Seurat object. For *Fzd7* virtual knockout analysis, *Prrx1* ^+^ SuSCs from the E18.5 WT samples were selected. For *Pink1* virtual knockout analysis, *Prrx1* ^+^ SuSCs from the E18.5 craniosynostosis samples were selected. Raw count matrices were retrieved from the RNA assay of the Seurat object. Genes detected in at least 10 cells and in at least 1% of cells were retained for downstream analysis. Virtual knockout analysis was then performed using scTenifoldKnk with the following parameters: qc = FALSE, nc_nNet = 10, nc_nCells = 500, nc_scaleScores = TRUE, and nc_nComp = 3. The resulting diffRegulation output was used for downstream ranking and enrichment analyses.

### 4.12 Gene Set Enrichment Analysis and Leading‐Edge Gene Extraction

For downstream gene set enrichment analysis (GSEA) [24], genes from the scTenifoldKnk diffRegulation output were ranked according to the distance statistic in descending order. Enrichment analysis was performed using Gene Ontology Biological Process (GOBP) gene sets, with emphasis on pathways related to bone development, ossification, and osteoblast differentiation. For each enriched pathway, the leading‐edge subset was extracted to define the core responsive genes driving the enrichment signal. Leading‐edge genes from osteogenic or ossification‐related pathways were then used for metagene construction and spatial projection analysis.

### 4.13 Spatial Tendency Analysis

Spatial tendency analysis was performed using SOAPy [18] to identify genes showing distance‐dependent expression patterns relative to *Prrx1* ^+^ SuSC regions. *Prrx1* ^+^ SuSC clusters were converted into binary masks and processed by morphological dilation and erosion with a kernel size of 35 pixels. For statistical testing, proximal and distal zones were defined by a 50‐pixel search radius and a 10‐pixel exclusion zone; Wilcoxon rank‐sum tests were applied to compare expression between these zones. Spearman correlation coefficients were computed across five concentric distance intervals up to 100 pixels to assess monotonic trends. Polynomial regression models were fitted on external neighborhoods (radius = 100 pixels, sampling fraction = 5%) to detect non‐linear patterns. Genes were clustered into ten modules based on their regression trajectories, using a minimum expression‐range threshold of 0.03 and FDR‐corrected *p* < 0.05.

### 4.14 Transmission Electron Microscopy (TEM)

Coronal suture tissues from P3 mice were microdissected and immediately fixed in 2.5% glutaraldehyde in buffer (pH 7.4). Samples were post‐fixed in 1% osmium tetroxide, dehydrated through a graded ethanol series, and embedded in epoxy resin. Ultrathin sections (∼70 nm) were prepared, mounted on copper grids, and stained with uranyl acetate and lead citrate. Images were acquired using a transmission electron microscope at the indicated magnifications. For quantitative analysis, images were obtained from the coronal suture midline region. Structures were scored as mitochondria(‐like) profiles enclosed within autophagosomes/autolysosomes on the basis of morphology. Images were coded before quantification, and scoring was performed by an investigator blinded to genotype/group assignment. The number of such structures was quantified for each biological replicate, and statistical analyses were performed using *n* = 5 mice per group.

### 4.15 Micro‐CT Scanning, Reconstruction, and Visualization

Cranial imaging was performed at the indicated ages to assess coronal suture patency and fusion. For P7 samples, skulls were scanned using a Skyscan 1276 micro‐CT instrument (Bruker microCT, Kontich, Belgium) with the following settings: source voltage, 55 kV; source current, 200 µA; 0.25‐mm Al filter; pixel size, 6 µm; and rotation step, 0.3°. Images were reconstructed using NRecon software (Bruker microCT, Kontich, Belgium). NRecon‐reconstructed P7 datasets were rendered and exported in CTvox (v3.3.1).

For P14 and P28 samples, cranial imaging was performed using a Quantum FX micro‐CT imaging system (PerkinElmer, USA) at a voxel size of 50 µm. 3D reconstruction and visualization were performed in 3D Slicer (v5.10.0). Representative 3D views were exported directly from 3D Slicer using a scripted screenshot workflow to ensure consistent visualization settings, including display range/threshold and camera/view orientation, across groups. Coronal suture patency or fusion was assessed morphologically from reconstructed micro‐CT images using matched viewing orientation and display settings across groups.

Sutures were classified as patent when a visible midline gap remained between the frontal and parietal bones, and as fused when the gap was no longer discernible and continuous osseous bridging was observed across the suture. These assessments were based on grossly apparent morphology in the reconstructed images.

### 4.16 Isolation and Culture of Suture Mesenchymal Cells

Following the protocol of Maruyama et al. [58, 59], coronal suture tissue was microdissected from mouse skulls and immediately incubated in DMEM (ThermoFisher, 31600) containing 0.2% collagenase, 10 mM HEPES (Solarbio, H8090), and penicillin–streptomycin (ThermoFisher, 15640055) at 37°C with gentle agitation (200 × *g*) for 60 min. The digest was filtered through a 40 µm nylon strainer and centrifuged (400 × *g*, 4°C, 7 min). Cell pellets were then resuspended in DMEM supplemented with 15% FBS and incubated for downstream assays. Cells were maintained at 37°C in a humidified atmosphere containing 5% CO_2_. The culture medium was changed every 2–3 days. For each biological replicate, suture mesenchymal cells were isolated from pooled coronal sutures of three littermates of the same genotype to obtain sufficient cell numbers for downstream osteogenic induction. Each pooled sample was treated as one biological replicate.

### 4.17 Flow Cytometric Analysis and Sorting of Reporter‐Labeled *Prrx1*‐Lineage SuSCs

Flow cytometric analysis was performed using a BD FACSCelesta (BD Biosciences), and fluorescence‐activated cell sorting was carried out on a BD FACSAria III (BD Biosciences). Coronal suture tissues were dissociated into single‐cell suspensions as described above and washed 1–2 times with pre‐cooled staining buffer consisting of phosphate‐buffered saline (PBS) supplemented with 2% fetal bovine serum (FBS) and 1 mM EDTA. Cells were resuspended in the same buffer and stained with DAPI (Thermo Fisher Scientific; 0.1 µg/100 µl) for 5 min in the dark immediately before analysis or sorting to exclude non‐viable cells.

For flow cytometric gating, debris was first excluded on the basis of forward‐ and side‐scatter parameters, followed by singlet discrimination using FSC‐A versus FSC‐H gating. Reporter‐positive *Prrx1*‐lineage suture mesenchymal cells were then defined on the basis of fluorescent reporter signal in dissociated tissues from *Prrx1^EGFP^* or *Prrx1^CreERT2^*; *R26^tdTomato^* reporter mice (Figure S6A,B). Specifically, the singlet population was gated on BB515 fluorescence to isolate *Prrx1*‐EGFP^+^ cells or on PE‐CF594 fluorescence to isolate *Prrx1*‐tdTomato^+^ cells for downstream analyses. Flow cytometry data were analyzed using FlowJo software (v10.8.1).

### 4.18 siRNAs Transfection

siRNAs targeting *Fzd7* and *Pink1*, together with a non‐targeting negative control siRNA, were designed and synthesized by Shanghai GenePharma Co., Ltd. Sequences are provided in Table S4. Transfection was performed using Lipofectamine 3000 (Thermo Fisher Scientific, L3000008) at an siRNA:Lipofectamine 3000 ratio of 25:1 (pmol:µL) according to the manufacturer's instructions. For co‐silencing experiments, *Fzd7* and *Pink1* siRNAs were co‐transfected under the same conditions.

### 4.19 In Vitro *Fzd7* Overexpression

Cells were transduced with an AAV9 vector carrying mouse *Fzd7* under the control of the CAG promoter (pAAV‐CAG‐Fzd7‐3 × FLAG‐tWPA; OBiO Technology, Shanghai, China) or the corresponding empty control vector at a multiplicity of infection (MOI) of 50 for 8 h. After transduction, cells were maintained under standard culture conditions and subjected to downstream analyses at the indicated time points.

### 4.20 Osteogenic Differentiation and Alizarin Red S Staining

For osteogenic differentiation assessment, suture mesenchymal cells were cultured until 70% confluence, then switched to OriCell Mouse Bone Marrow Mesenchymal Stem Cell Osteogenic Differentiation Medium (MUXMX‐90021) with media changes every 3 days. After 14 days of induction, calcium mineralization was evaluated by Alizarin Red S staining, where cells were fixed with 4% paraformaldehyde, stained with 2% Alizarin Red S solution (pH 4.2) for 20 min, and mineralized nodules were visualized under light microscopy. Statistical analyses of Alizarin Red S staining quantification were performed using one‐way ANOVA followed by Šídák's multiple comparisons test. Variance homogeneity was verified (Brown‐Forsythe test, ns). Data represent three or four independent biological replicates, each biological replicate consisted of suture mesenchymal cells pooled from the sutures of three E18.5 donor animals.

### 4.21 Mito‐Keima Mitophagy Assay

SuSCs were infected with a mitochondria‐targeted Keima adenovirus (COX8–mt‐mKeima; GeneChem, Shanghai, China; cat. H20788; vector GV269) at a multiplicity of infection (MOI) of 30 for 8 h and imaged 48 h after infection. Live‐cell imaging was performed on a Leica TCS SP8 laser‐scanning confocal microscope (LAS X v3.5.7; Leica Microsystems) using sequential dual excitation of mtKeima at 488 and 552 nm, with emission collected at 620 nm. Image pairs were acquired sequentially for each field. For quantification, the mitophagy readout was defined as the fraction of mtKeima signal in the lysosomal/red channel, calculated as the red‐to‐total intensity area ratio [red/(red + green)] using ImageJ. Three randomly selected fields were analyzed for each biological replicate, and the mean value per biological replicate was used for statistical analysis. Quantified values from five independent biological replicates per group were used for statistical analysis.

### 4.22 Mito‐Tracker/Lyso‐Tracker Colocalization Assay

SuSCs were subjected to live‐cell staining with Lyso‐Tracker Red (Beyotime Biotechnology, C1046) and Mito‐Tracker Green (Beyotime Biotechnology, C1048) according to the manufacturer's instructions. Confocal imaging was performed on a Leica TCS SP8 laser‐scanning confocal microscope (LAS X v3.5.7; Leica Microsystems). Images were acquired in sequential scanning mode using 488 nm excitation for Mito‐Tracker Green and 552 nm excitation for Lyso‐Tracker Red. Colocalization between mitochondrial and lysosomal signals was quantified in ImageJ using the thresholded Manders’ M1 coefficient, defined as the fraction of mitochondrial (Mito‐Tracker Green) signal overlapping with lysosomal (Lyso‐Tracker Red) signal. Three randomly selected fields were analyzed for each biological replicate, and the mean value per biological replicate was used for statistical analysis. Quantified values from five independent biological replicates per group were used for statistical analysis.

### 4.23 Masson's Trichrome Staining

Coronal suture specimens were collected and fixed in 4% paraformaldehyde overnight at 4°C. Depending on the age of the mice, samples were decalcified in EDTA at 4°C until adequately softened for processing. After decalcification, tissues were dehydrated, paraffin‐embedded, and sectioned at 5 µm. Masson's trichrome staining was performed according to manufacturer's instructions (Beijing Solarbio Science & Technology Co., Ltd; cat. no. G1346). Stained sections were used to evaluate coronal suture morphology, including osteoid/bone bridging across the suture midline and preservation of the midline gap. Representative histological images were selected from comparable anatomical levels of the coronal suture region across experimental groups.

### 4.24 ROS Assay

Intracellular ROS levels were assessed using a DCFH‐DA‐based ROS assay kit (Beyotime Biotechnology; cat. no. S0033) according to the manufacturer's instructions. Equal numbers of *Prrx1* ^+^ SuSCs were seeded in each well before staining. Cells were incubated with DCFH‐DA working solution and washed to remove extracellular probe. DCF fluorescence was measured using a microplate reader at Ex/Em 488/525 nm. Blank correction was applied during plate‐reader acquisition. Rosup‐treated cells were used as positive controls to validate assay responsiveness. For quantification, technical replicate wells were averaged within each independent biological replicate. To account for inter‐experimental variation, DCF fluorescence values were expressed relative to the mean value of the matched control group within the same independent experiment. WT cells served as the matched control for the disease‐state comparison, and WT + si‐NC cells served as the matched control for the *Fzd7*‐knockdown comparison.

### 4.25 JC‐1 Mitochondrial Membrane Potential Assay

Mitochondrial membrane potential was assessed using an enhanced JC‐1 mitochondrial membrane potential assay kit (Beyotime Biotechnology; cat. no. C2003) according to the manufacturer's instructions. Equal numbers of *Prrx1* ^+^ SuSCs were seeded in each well before staining. After incubation with JC‐1 working solution, cells were washed and fluorescence was measured using a microplate reader. JC‐1 monomer fluorescence was measured at Ex/Em 490/530 nm, and JC‐1 aggregate fluorescence was measured at Ex/Em 525/590 nm. Blank correction was applied during plate‐reader acquisition. Mitochondrial membrane potential was quantified as the JC‐1 aggregate/monomer fluorescence ratio. CCCP‐treated cells were used as positive controls for mitochondrial depolarization. Technical replicate wells were averaged within each independent biological replicate before statistical analysis.

### 4.26 Western Blotting

Cells were lysed using a centrifuge column–based total protein extraction kit with strong lysis buffer (Beyotime Biotechnology; cat. no. P0013WS) to obtain total protein, and protein concentrations were determined using a BCA protein assay kit (Beyotime Biotechnology; cat. no. P0009). Equal amounts of protein were separated on 12% Bis‐Tris polyacrylamide gels and transferred to polyvinylidene fluoride membranes (Immobilon‐P PVDF membrane, Millipore; cat. no. IPVH00010). Membranes were blocked with 5% nonfat milk and incubated with primary antibodies overnight at 4°C, followed by incubation with the corresponding secondary antibodies for 1 h at room temperature. Protein bands were detected using SuperSignal West Pico PLUS Chemiluminescent Substrate (Thermo Scientific; cat. no. 34579). Where needed, membranes were stripped using Restore Western Blot Stripping Buffer (Thermo Scientific; cat. no. 21059) and reprobed with additional antibodies. Band intensities were quantified using Image Lab (v6.1). For Wnt pathway‐associated analyses, active β‐catenin was detected using an antibody recognizing non‐phosphorylated β‐catenin at Ser 45 (Cell Signaling Technology, #19807), and phosphorylated SAPK/JNK was detected using an antibody recognizing phosphorylation at Thr183/Tyr185 (Cell Signaling Technology, #9251). For p‐JNK quantification, the combined densitometric intensity of the p46 and p54 phospho‐JNK bands was measured. Antibody information is provided in Table S1.

### 4.27 Immunofluorescence Staining

For tissue immunofluorescence, sections were brought to room temperature, rinsed in PBS, permeabilized with PBS containing 0.3% Triton X‐100 for 15 min, and blocked with 5% goat serum for 30 min. Sections were then incubated with primary antibodies overnight at 4°C, followed by incubation with fluorophore‐conjugated secondary antibodies for 1 h at room temperature. Nuclei were counterstained with DAPI, and slides were mounted with antifade mounting medium. Images were acquired using a Leica SP8 confocal microscope and processed in LAS X software (v3.7.5). Antibody information is provided in Table S1.

### 4.28 Statistical Analysis

Sample sizes, biological replicates, and statistical details for individual experiments are provided in the text, Figure legends, or the corresponding Methods sections. For scRNA‐seq differential expression, Gene Ontology enrichment, and GSEA‐based analyses, statistical testing and multiple‐testing correction were performed as described in the relevant analysis sections. For quantitative experimental data, including mtKeima imaging, mitochondria–lysosome colocalization, TEM measurements, immunoblot densitometry, and mineralization assays, statistical analyses were performed using GraphPad Prism 10 (GraphPad Software). Data are presented as mean ± SEM unless otherwise indicated. For comparisons between two groups, unpaired two‐tailed Student's t‐test was used unless otherwise stated. For comparisons among three or more groups, one‐way or two‐way ANOVA was used as appropriate, followed by the multiple‐comparison test specified in the corresponding Figure legend or Methods subsection. Differences were considered statistically significant at *p < 0.05, **p < 0.01, ***p < 0.001, and ****p < 0.0001. ns, not significant. For ROS and JC‐1 assays, statistical analysis was performed using pre‐specified pairwise comparisons. The main biological comparisons were WT versus CS and WT + si‐NC versus WT + si‐*Fzd7*. Positive‐control groups were included to validate assay responsiveness and were analyzed only against the corresponding untreated or control groups. Technical replicate wells were averaged before biological replicates were used for statistical testing. When more than two groups were displayed in the same graph, one‐way ANOVA followed by Šídák's multiple‐comparison test was used for selected pairwise comparisons.

## Author Contributions

XLJ and XYC conceived the study, generated hypotheses, and designed experiments. XYC, ZC, CZL, TH, HG, JS, YYY, and YW performed experiments and analyzed the data. XYC and ZC collected samples and conducted the bioinformatics analysis. XYC and XLJ wrote, reviewed, and edited the paper. XLJ and GDS supervised the project.

## Funding

This work is supported by the National Natural Science Foundation of China (Grant No. 82402947, CZL), the Beijing Natural Science Foundation (Grant No. 7242121, XLJ), and the Scientific Foundation of Plastic Surgery Hospital, Chinese Academy of Medical Sciences (Grant No. YSZ2024CG001, XLJ).

## Conflicts of Interest

The authors declare no conflicts of interest.

## Code Availability

The SpatialCell pipeline for spatial cell identification has been made publicly available on GitHub at: https://github.com/Xinyan‐C/Spatialcell. Jupyter notebooks needed to reproduce our analyses (examples/SpatialCell_Demo.ipynb) are included in that repository. A minimal example dataset for E14.5, E18.5, and P3 is archived on Zenodo (https://zenodo.org/records/19012914).

## Supporting information




**Supporting File 1**: advs77245‐sup‐0001‐SuppMat.pdf.

**Supporting File 2**: advs77245‐sup‐0002‐DataS1.xls.

**Supporting File 3**: advs77245‐sup‐0003‐DataS2.csv.

## Data Availability

The sequencing datasets in this study are publicly available in the Gene Expression Omnibus under accession numbers GSE303344 and GSE303460. All other data are included in the article and Supplementary Information or are available from the corresponding authors upon reasonable request.
