# Supplementary material for: Fzd7 Restrains Pink1‐Dependent Mitophagy in Suture Stem Cells to Maintain Cranial Suture Patency
Source: Adv Sci (Weinh). 2026 Aug 19;13(62):e77245. doi: 10.1002/advs.77245 (PMC13488484; doi:10.1002/advs.77245)
Supplement: Supplementary file 1 [file ADVS-13-e77245-s003.pdf]

Supplementary Materials for

Fzd7 restrains *Pink1*-dependent mitophagy in suture stem cells to maintain cranial suture patency

Xinyan Chen *et al.*

*Corresponding author: Xiaolei Jin, Email: jinxiaolei@psh.pumc.edu.cn

This PDF file includes:

Figs. S1 to S14
Tables S1 to S4

Other Supplementary Materials for this manuscript include the following:
Data S1 to S2

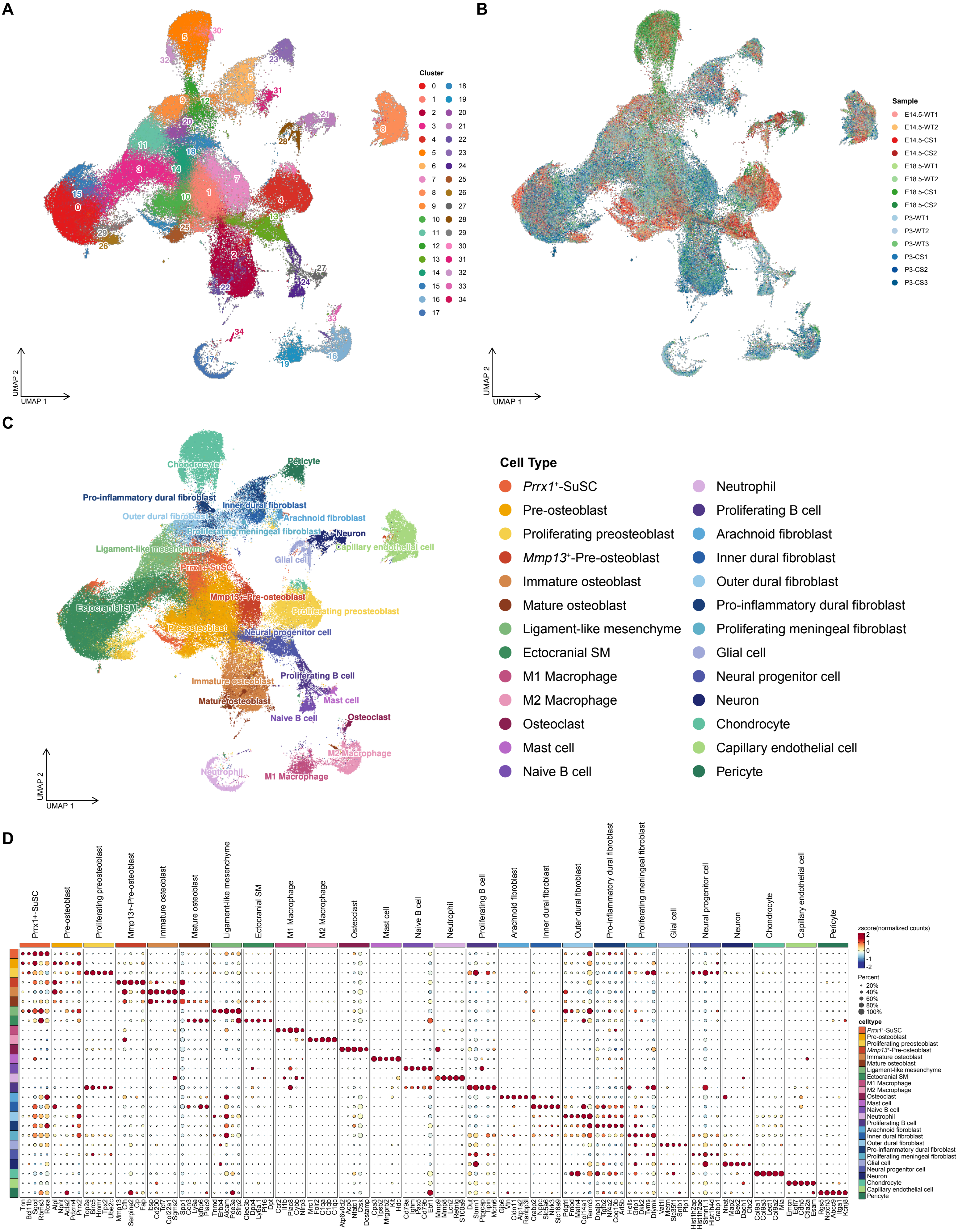

A
B
C
D
Cluster
0 1 2 3 4 5 6 7 8 9 10 11 12 13 14 15 16 17 18 19 20 21 22 23 24 25 26 27 28 29 30 31 32 33 34
UMAP 2
UMAP 1
Sample
E14.5-WT1
E14.5-WT2
E14.5-CS1
E14.5-CS2
E18.5-WT1
E18.5-WT2
E18.5-CS1
E18.5-CS2
P3-WT1
P3-WT2
P3-WT3
P3-CS1
P3-CS2
P3-CS3
Cell Type
Prrx1+-SuSC
Pre-osteoblast
Proliferating preosteoblast
Mmp13+-Pre-osteoblast
Immature osteoblast
Mature osteoblast
Ligament-like mesenchyme
Ectocranial SM
M1 Macrophage
M2 Macrophage
Osteoclast
Mast cell
Naive B cell
Neutrophil
Proliferating B cell
Arachnoid fibroblast
Inner dural fibroblast
Outer dural fibroblast
Pro-inflammatory dural fibroblast
Proliferating meningeal fibroblast
Glial cell
Neural progenitor cell
Neuron
Chondrocyte
Capillary endothelial cell
Pericyte
zscore(normalized counts)
Percent
20%
40%
60%
80%
100%
celltype

Fig. S1. Overview of integrated coronal suture scRNA-seq data and cell-type annotation

A-B. UMAP of integrated coronal suture scRNA-seq data across stages and genotypes. Each dot represents one cell (142,579 cells).

(A) Cells colored by Seurat clusters.

(B) Cells colored by sample. CS, *Fgfr2*$^{C342Y/+}$ craniosynostosis model; WT, wild-type.

C. UMAP of integrated coronal suture scRNA-seq data across stages and genotypes. Each dot represents one cell. Cells were colored by celltypes.

D. Dot plot showing the expression of markers for each cell type.

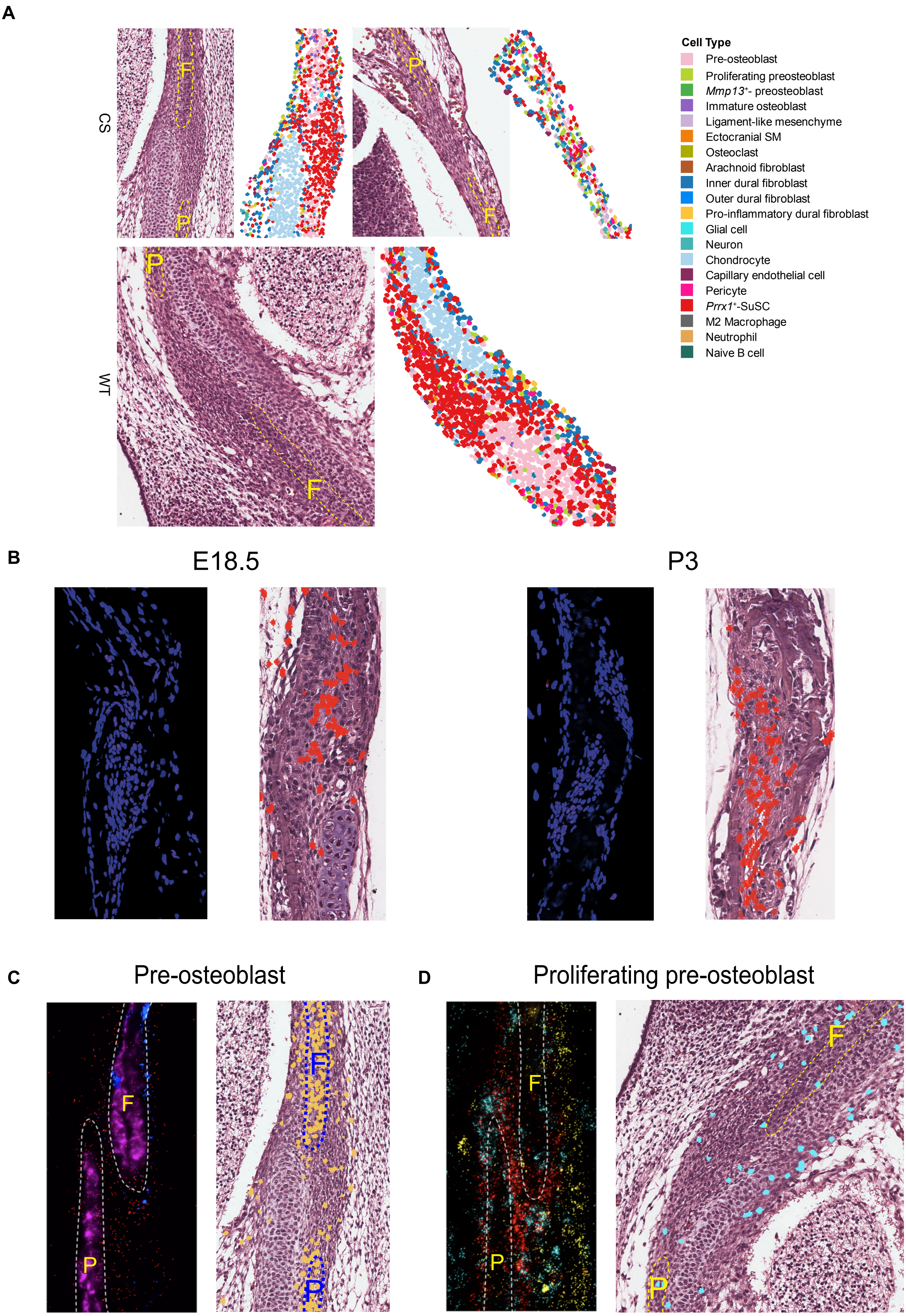
A
CS
WT
F
P
Cell Type
Pre-osteoblast
Proliferating preosteoblast
Mmp13⁺- preosteoblast
Immature osteoblast
Ligament-like mesenchyme
Ectocranial SM
Osteoclast
Arachnoid fibroblast
Inner dural fibroblast
Outer dural fibroblast
Pro-inflammatory dural fibroblast
Glial cell
Neuron
Chondrocyte
Capillary endothelial cell
Pericyte
Prrx1⁺-SuSC
M2 Macrophage
Neutrophil
Naive B cell
B
E18.5
P3
C
Pre-osteoblast
D
Proliferating pre-osteoblast

Fig. S2. Spatial mapping and validation of Visium HD cell-type annotation

A. Spatial mapping of annotated cell types in E14.5 coronal suture sections from CS and WT mice on hematoxylin and eosin (H&E) images. F, frontal bone; P, parietal bone.

B-D. Validation of Visium HD cell-type annotation by comparison with independent marker patterns. F, frontal bone; P, parietal bone.

(B) The immunofluorescence images of *Prrx1* compared with SpatialCell annotations on H&E staining. *Prrx1* is shown in red and DAPI in blue.

(C) Published *Ibsp* expression pattern for pre-osteoblasts (Adapted from Holmes *et al.*(*11*) (2021) under the terms of the CC BY license. Copyright © 2021 Holmes *et al.*, published by Springer Nature) compared with SpatialCell annotations on H&E staining in this study.

(D) Published *Top2a* expression pattern for proliferating pre-osteoblasts (Adapted from Holmes *et al.*(*11*) (2021) under the terms of the CC BY license. Copyright © 2021 Holmes *et al.*, published by Springer Nature) compared with SpatialCell annotations on H&E staining in this study.

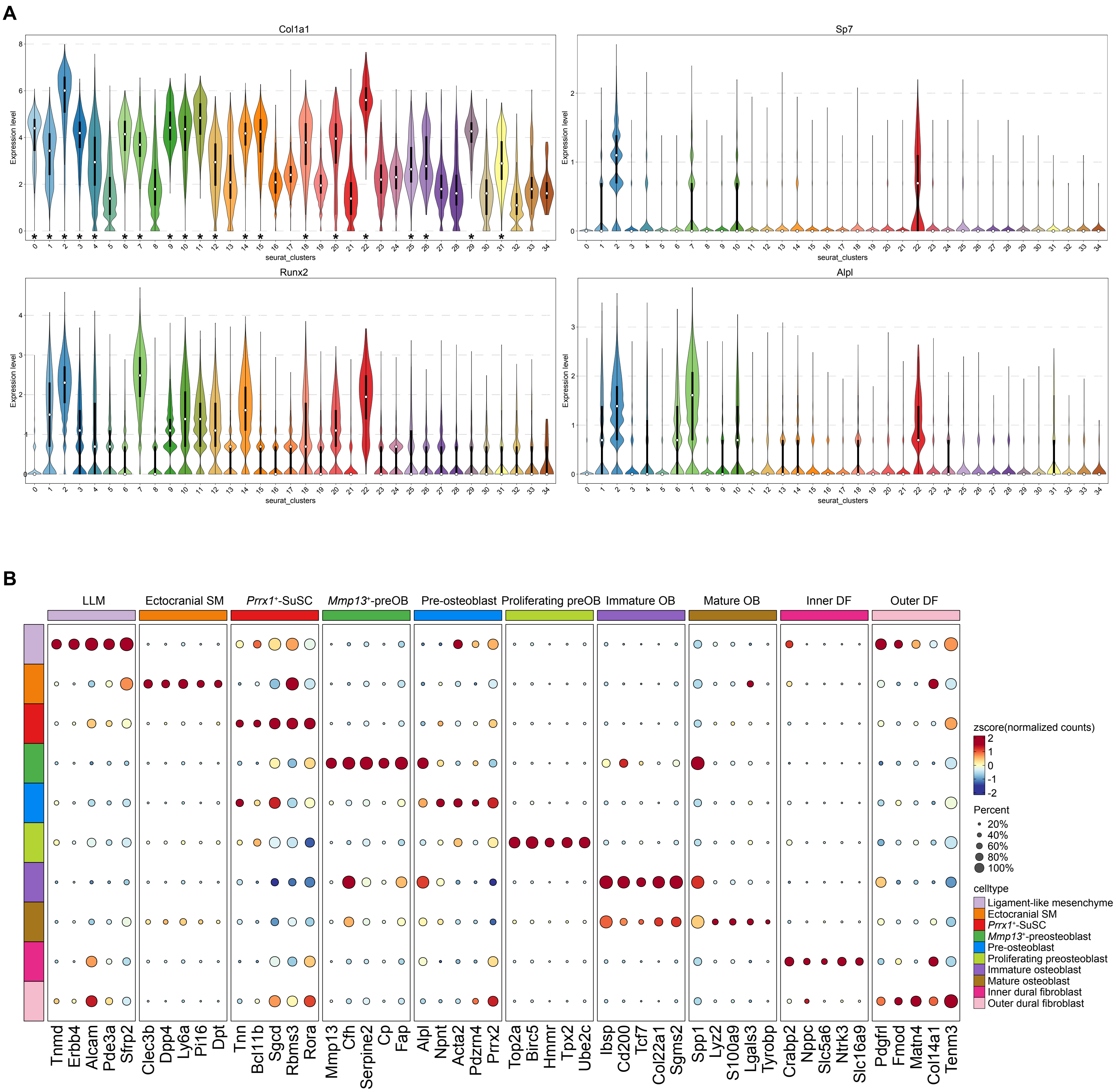
A
Col1a1
Sp7
Runx2
Alpl
Expression level
seurat_clusters
B
LLM
Ectocranial SM
Prrx1+-SuSC
Mmp13+-preOB
Pre-osteoblast
Proliferating preOB
Immature OB
Mature OB
Inner DF
Outer DF
Tnmd Erbb4 Alcam Pde3a Sfrp2 Clec3b Dpp4 Ly6a Pi16 Dpt Tnn Bcl11b Sgcd Rbms3 Rora Mmp13 Cfh Serpine2 Cp Fap Alpl Npnt Acta2 Pdzrn4 Prrx2 Top2a Birc5 Hmmr Tpx2 Ube2c Ibsp Cd200 Tcf7 Col22a1 Sgms2 Spp1 Lyz2 S100a9 Lgals3 Tyrobp Crabp2 Nppc Slc5a6 Ntrk3 Slc16a9 Pdgfrl Fmod Matn4 Col14a1 Tenm3
zscore(normalized counts)
Percent
20%
40%
60%
80%
100%
celltype
Ligament-like mesenchyme
Ectocranial SM
Prrx1+-SuSC
Mmp13+-preosteoblast
Pre-osteoblast
Proliferating preosteoblast
Immature osteoblast
Mature osteoblast
Inner dural fibroblast
Outer dural fibroblast

Fig. S3. Identification and annotation of suture mesenchymal cell populations in integrated scRNA-seq data

A. scRNA-seq identification of suture mesenchymal cells. Violin plots of marker genes (*Col1a1*, *Sp7*, *Runx2* and *Alpl*) with selected populations marked by asterisks.

B. Dot plot showing the expression of suture mesenchymal cell markers. OB, osteoblast; LLM, Ligament-like mesenchyme; SM, Suture mesenchyme cell; DF, dural fibroblast.

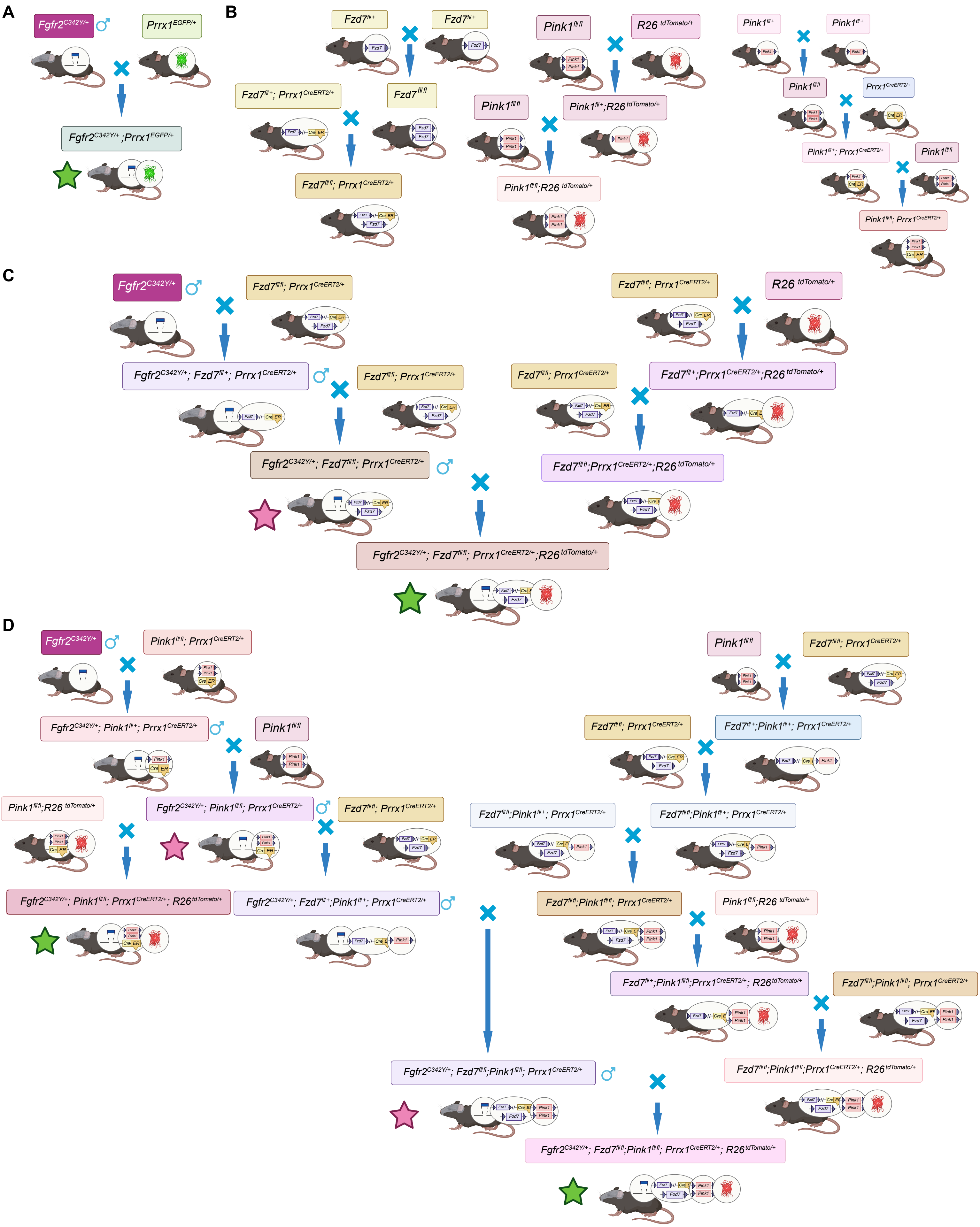
A
$Fgfr2^{C342Y/+}$ ♂
$Prrx1^{EGFP/+}$
$Fgfr2^{C342Y/+}$;$Prrx1^{EGFP/+}$
B
$Fzd7^{fl/+}$
$Fzd7^{fl/+}$
$Fzd7^{fl/+}$; $Prrx1^{CreERT2/+}$
$Fzd7^{fl/fl}$
$Fzd7^{fl/fl}$; $Prrx1^{CreERT2/+}$
$Pink1^{fl/fl}$
$R26^{tdTomato/+}$
$Pink1^{fl/fl}$
$Pink1^{fl/+}$;$R26^{tdTomato/+}$
$Pink1^{fl/fl}$;$R26^{tdTomato/+}$
$Pink1^{fl/+}$
$Pink1^{fl/+}$
$Pink1^{fl/fl}$
$Prrx1^{CreERT2/+}$
$Pink1^{fl/+}$; $Prrx1^{CreERT2/+}$
$Pink1^{fl/fl}$
$Pink1^{fl/fl}$; $Prrx1^{CreERT2/+}$
C
$Fgfr2^{C342Y/+}$ ♂
$Fzd7^{fl/fl}$; $Prrx1^{CreERT2/+}$
$Fgfr2^{C342Y/+}$; $Fzd7^{fl/+}$; $Prrx1^{CreERT2/+}$ ♂
$Fzd7^{fl/fl}$; $Prrx1^{CreERT2/+}$
$Fgfr2^{C342Y/+}$; $Fzd7^{fl/fl}$; $Prrx1^{CreERT2/+}$ ♂
$Fzd7^{fl/fl}$; $Prrx1^{CreERT2/+}$
$R26^{tdTomato/+}$
$Fzd7^{fl/fl}$; $Prrx1^{CreERT2/+}$
$Fzd7^{fl/+}$;$Prrx1^{CreERT2/+}$;$R26^{tdTomato/+}$
$Fzd7^{fl/fl}$;$Prrx1^{CreERT2/+}$;$R26^{tdTomato/+}$
$Fgfr2^{C342Y/+}$; $Fzd7^{fl/fl}$; $Prrx1^{CreERT2/+}$;$R26^{tdTomato/+}$
D
$Fgfr2^{C342Y/+}$ ♂
$Pink1^{fl/fl}$; $Prrx1^{CreERT2/+}$
$Fgfr2^{C342Y/+}$; $Pink1^{fl/+}$; $Prrx1^{CreERT2/+}$ ♂
$Pink1^{fl/fl}$
$Pink1^{fl/fl}$;$R26^{tdTomato/+}$
$Fgfr2^{C342Y/+}$; $Pink1^{fl/fl}$; $Prrx1^{CreERT2/+}$ ♂
$Fzd7^{fl/fl}$; $Prrx1^{CreERT2/+}$
$Fgfr2^{C342Y/+}$; $Pink1^{fl/fl}$; $Prrx1^{CreERT2/+}$; $R26^{tdTomato/+}$
$Fgfr2^{C342Y/+}$; $Fzd7^{fl/+}$;$Pink1^{fl/+}$; $Prrx1^{CreERT2/+}$ ♂
$Pink1^{fl/fl}$
$Fzd7^{fl/fl}$; $Prrx1^{CreERT2/+}$
$Fzd7^{fl/fl}$; $Prrx1^{CreERT2/+}$
$Fzd7^{fl/+}$;$Pink1^{fl/+}$; $Prrx1^{CreERT2/+}$
$Fzd7^{fl/fl}$;$Pink1^{fl/+}$; $Prrx1^{CreERT2/+}$
$Fzd7^{fl/fl}$;$Pink1^{fl/+}$; $Prrx1^{CreERT2/+}$
$Fzd7^{fl/fl}$;$Pink1^{fl/fl}$; $Prrx1^{CreERT2/+}$
$Pink1^{fl/fl}$;$R26^{tdTomato/+}$
$Fzd7^{fl/+}$;$Pink1^{fl/fl}$;$Prrx1^{CreERT2/+}$; $R26^{tdTomato/+}$
$Fzd7^{fl/fl}$;$Pink1^{fl/fl}$; $Prrx1^{CreERT2/+}$
$Fgfr2^{C342Y/+}$; $Fzd7^{fl/fl}$;$Pink1^{fl/fl}$; $Prrx1^{CreERT2/+}$ ♂
$Fzd7^{fl/fl}$;$Pink1^{fl/fl}$;$Prrx1^{CreERT2/+}$; $R26^{tdTomato/+}$
$Fgfr2^{C342Y/+}$; $Fzd7^{fl/fl}$;$Pink1^{fl/fl}$; $Prrx1^{CreERT2/+}$; $R26^{tdTomato/+}$

Fig. S4. Breeding strategy for generation of reporter, conditional knockout, and epistasis mouse cohorts used in this study

A. Breeding scheme for generation of *Fgfr2*$^{C342Y/+}$; *Prrx1*^{EGFP} reporter mice.

B. Generation of intermediate cohorts used to improve target-genotype yield.

C. Breeding scheme for *Fzd7* conditional knockout and related reporter cohorts.

D. Breeding scheme for *Pink1* conditional knockout, *Fzd7*/*Pink1* double conditional knockout, and related reporter cohorts.

Green stars indicate the core reporter mice. Red stars indicate the main experimental/phenotypic mice. Male symbols indicate crosses in which the male breeder was specified. Some intermediate self-crossing steps used to improve target-genotype yield are omitted. Created in BioRender. Chen, X. (2026).

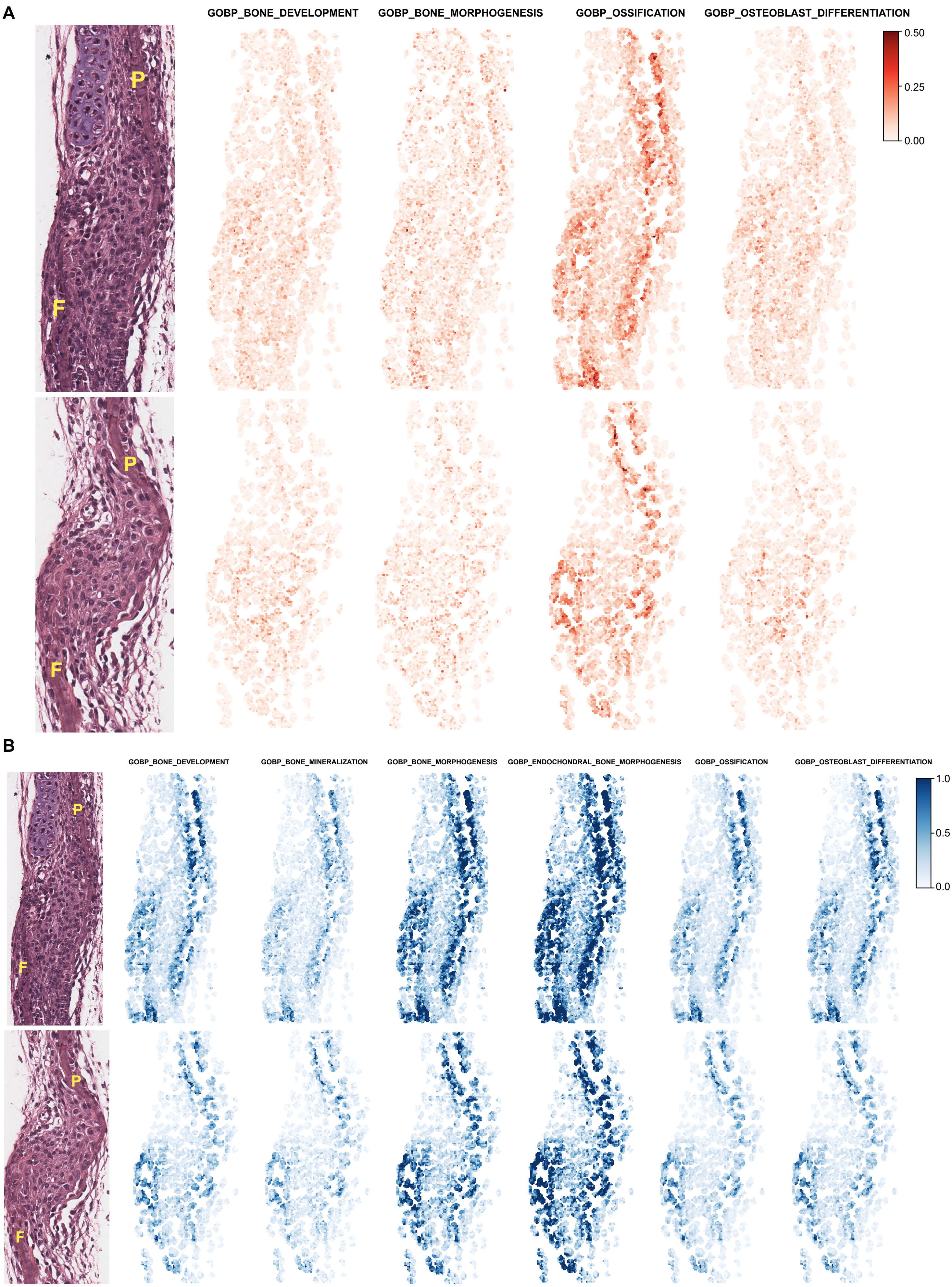

A
GOBP_BONE_DEVELOPMENT
GOBP_BONE_MORPHOGENESIS
GOBP_OSSIFICATION
GOBP_OSTEOBLAST_DIFFERENTIATION
0.50
0.25
0.00
P
F
P
F
B
GOBP_BONE_DEVELOPMENT
GOBP_BONE_MINERALIZATION
GOBP_BONE_MORPHOGENESIS
GOBP_ENDOCHONDRAL_BONE_MORPHOGENESIS
GOBP_OSSIFICATION
GOBP_OSTEOBLAST_DIFFERENTIATION
1.0
0.5
0.0
P
F
P
F

Fig. S5. Disrupted SuSC differentiation underlies premature osteogenesis in craniosynostosis

A. Spatial visualization of osteogenic pathway activity in E18.5 CS coronal sutures based on metagenes constructed from the core enrichment genes identified by GSEA of the scTenifoldKnk output for *Fzd7* virtual knockout. F, frontal bone; P, parietal bone.

B. Spatial visualization of osteogenic pathway activity in E18.5 CS coronal sutures based on metagenes constructed from the core enrichment genes identified by GSEA of the scTenifoldKnk output for *Pink1* virtual knockout. F, frontal bone; P, parietal bone.

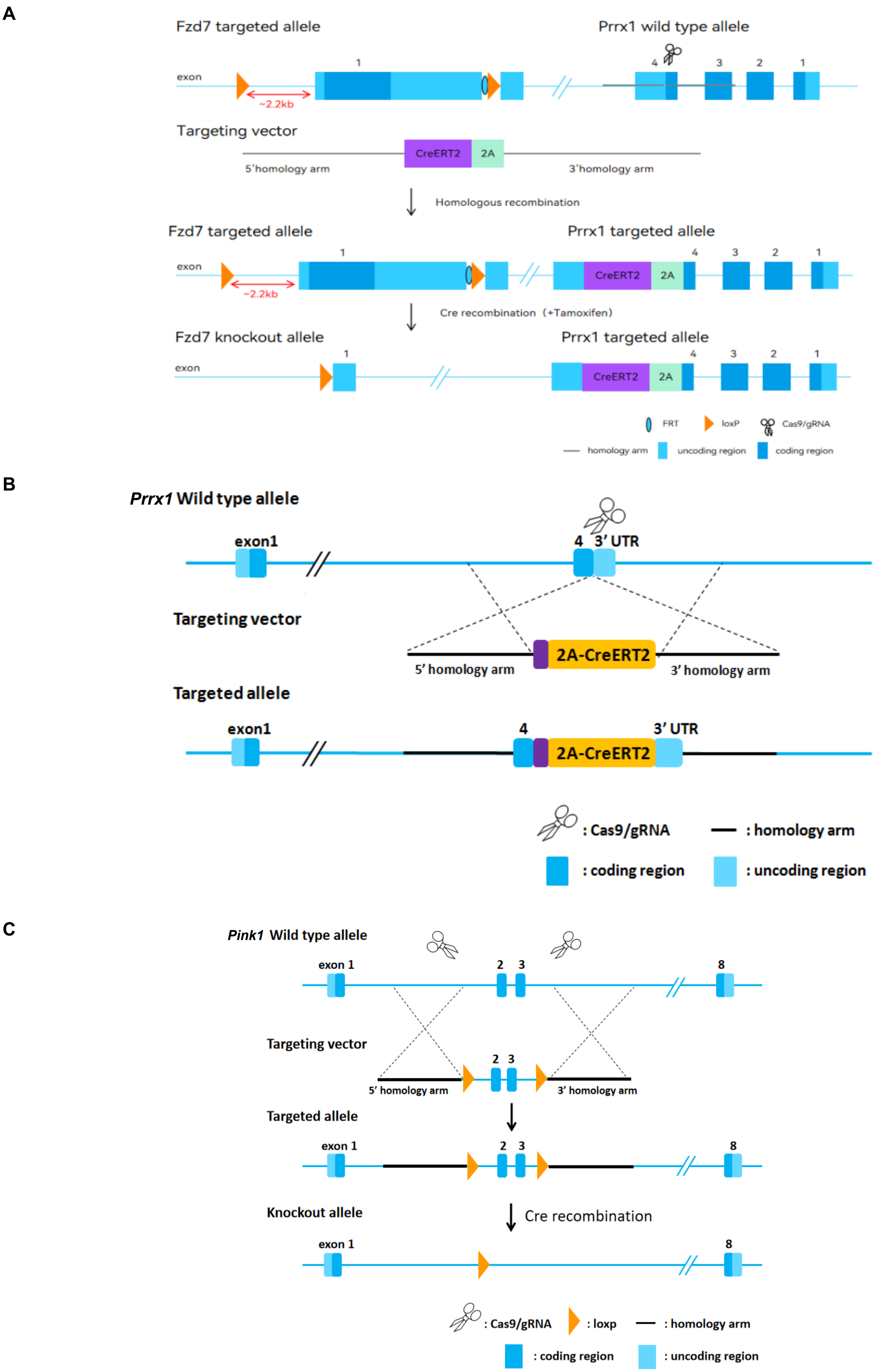
A
Fzd7 targeted allele
Prrx1 wild type allele
exon
~2.2kb
Targeting vector
CreERT2
2A
5'homology arm
3'homology arm
Homologous recombination
Fzd7 targeted allele
Prrx1 targeted allele
Cre recombination (+Tamoxifen)
Fzd7 knockout allele
Prrx1 targeted allele
FRT
loxP
Cas9/gRNA
homology arm
uncoding region
coding region
B
Prrx1 Wild type allele
exon1
4 3' UTR
Targeting vector
2A-CreERT2
5' homology arm
3' homology arm
Targeted allele
exon1
4
3' UTR
2A-CreERT2
: Cas9/gRNA
: homology arm
: coding region
: uncoding region
C
Pink1 Wild type allele
exon 1
2 3
8
Targeting vector
5' homology arm
3' homology arm
Targeted allele
Knockout allele
Cre recombination
: Cas9/gRNA
: loxp
: homology arm
: coding region
: uncoding region

Fig. S6. Targeting strategies for the genetically modified mouse alleles used in this study

A. Schematic representation of the strategy used to generate the linked *Fzd7*$^{fl/+}$; *Prrx1*$^{CreERT2/+}$allele. A *Prrx1*-2A-CreERT2 knock-in was introduced at the *Prrx1* locus on a chromosome carrying the *Fzd7* floxed allele, generating the linked *Fzd7*$^{fl/+}$; *Prrx1*$^{CreERT2/+}$ configuration. Upon tamoxifen induction, Cre-mediated recombination excises the floxed *Fzd7* region in *Prrx1*-lineage cells.

B. Schematic representation of the *Prrx1*$^{CreERT2/+}$ allele.

C. Schematic representation of the *Pink1*$^{fl/+}$ allele.

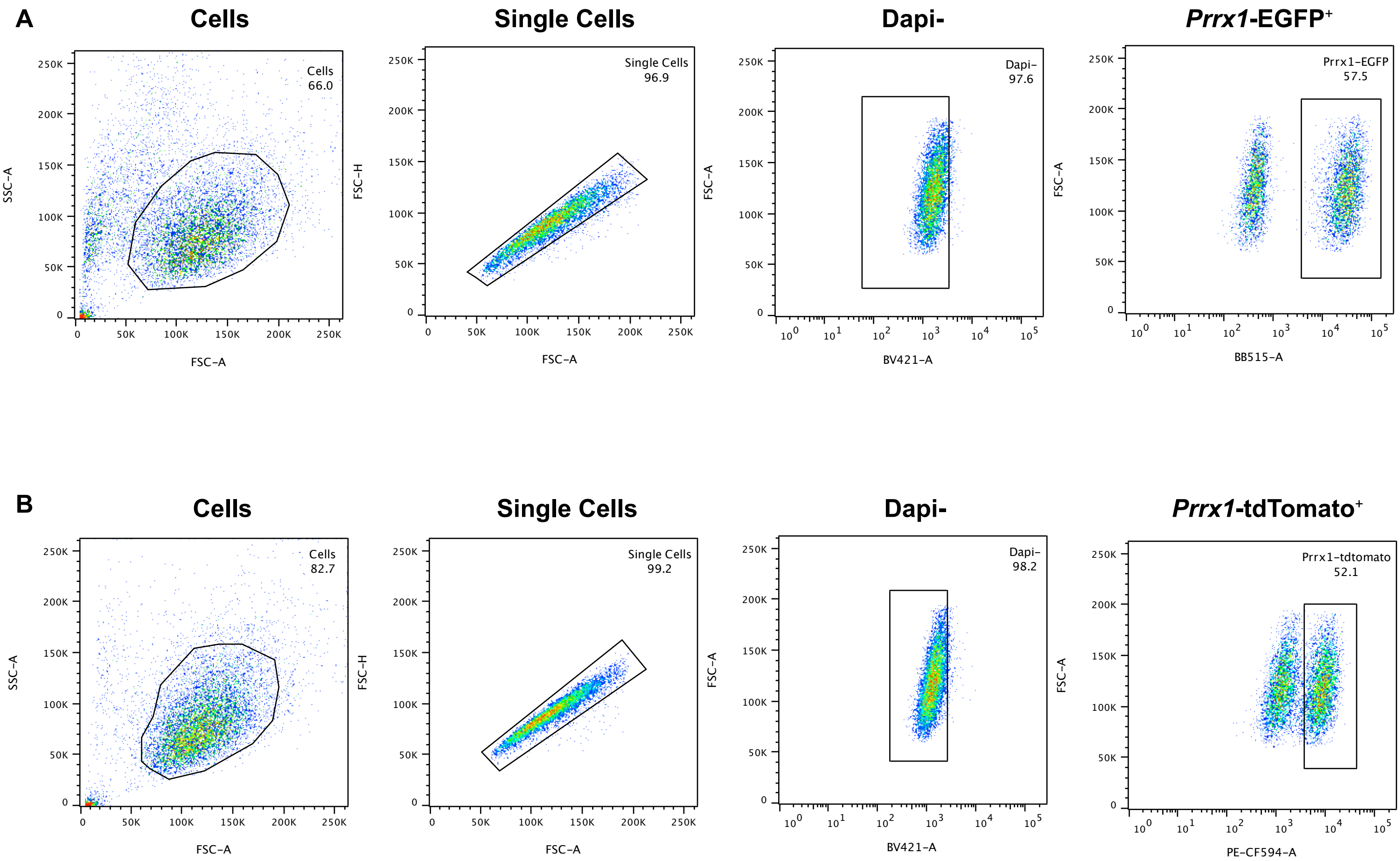

A
Cells
Cells
66.0
SSC-A
FSC-A
Single Cells
Single Cells
96.9
FSC-H
FSC-A
Dapi-
Dapi-
97.6
FSC-A
BV421-A
Prrx1-EGFP+
Prrx1-EGFP
57.5
FSC-A
BB515-A
B
Cells
Cells
82.7
SSC-A
FSC-A
Single Cells
Single Cells
99.2
FSC-H
FSC-A
Dapi-
Dapi-
98.2
FSC-A
BV421-A
Prrx1-tdTomato+
Prrx1-tdtomato
52.1
FSC-A
PE-CF594-A

Fig. S7. Gating strategy for fluorescence-activated cell sorting of reporter-labeled Prrx1-lineage suture mesenchymal cells

A. Representative gating strategy for fluorescence-activated cell sorting of *Prrx1*-$EGFP^+$ suture mesenchymal cells from coronal sutures. Total dissociated cells were first gated by forward scatter (FSC) and side scatter (SSC) to exclude debris and define the main cell population. Single cells were then selected by FSC-A versus FSC-H gating to exclude doublets. Viable cells were identified as DAPI-negative events, and the singlet viable population was subsequently gated on BB515 fluorescence to isolate *Prrx1*-$EGFP^+$ cells for downstream analyses.

B. Representative gating strategy for fluorescence-activated cell sorting of *Prrx1*-$tdTomato^+$ suture mesenchymal cells from coronal sutures. Total dissociated cells were first gated by forward scatter (FSC) and side scatter (SSC) to exclude debris and define the main cell population. Single cells were then selected by FSC-A versus FSC-H gating to exclude doublets. Viable cells were identified as DAPI-negative events, and the singlet viable population was subsequently gated on PE-CF594 fluorescence to isolate *Prrx1*-$tdTomato^+$ cells for downstream analyses.

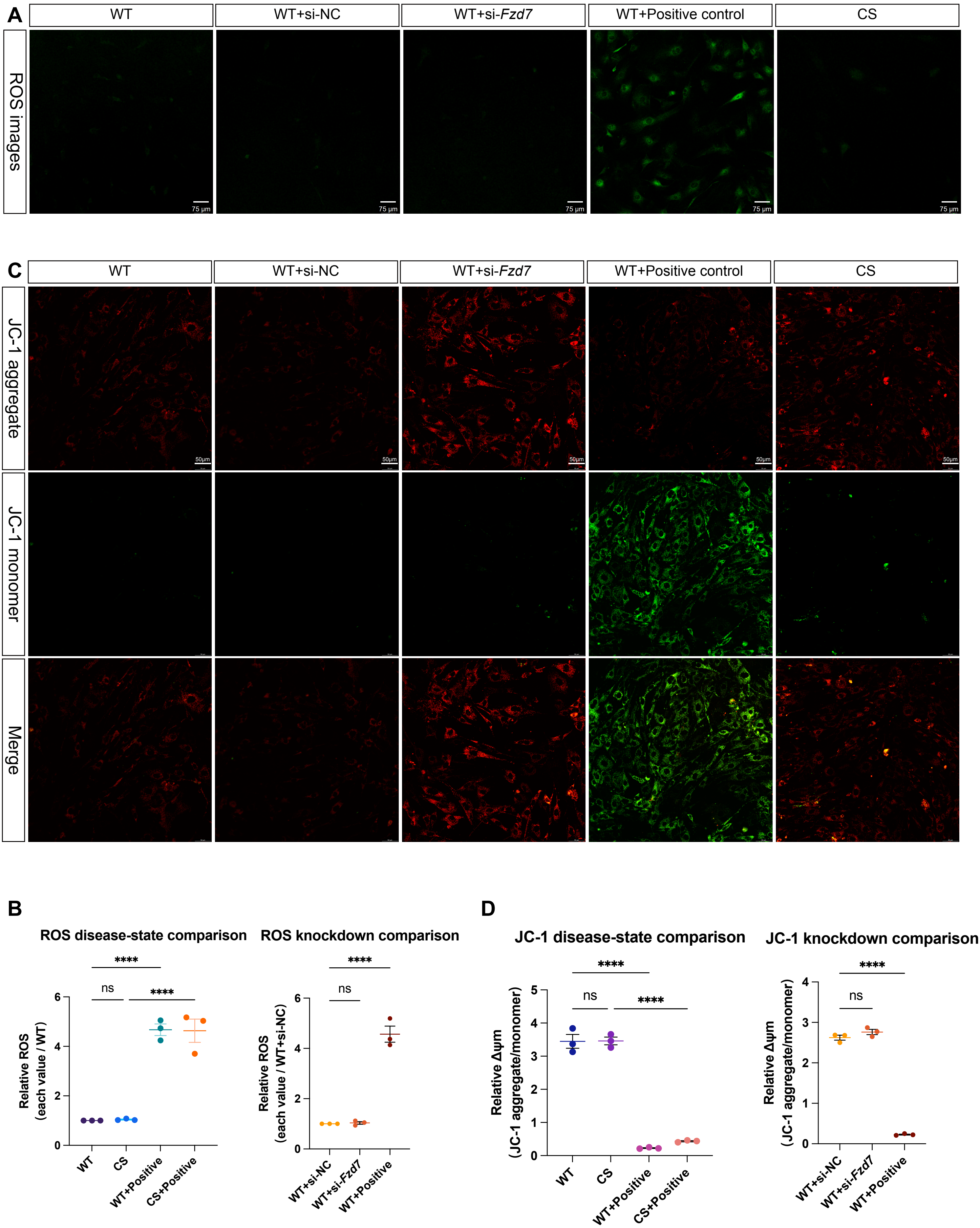
A
WT
WT+si-NC
WT+si-Fzd7
WT+Positive control
CS
ROS images
75 μm
C
JC-1 aggregate
JC-1 monomer
Merge
50μm
B
ROS disease-state comparison
ROS knockdown comparison
Relative ROS (each value / WT)
Relative ROS (each value / WT+si-NC)
ns
****
WT+Positive
CS+Positive
WT+si-Fzd7
D
JC-1 disease-state comparison
JC-1 knockdown comparison
Relative Δψm (JC-1 aggregate/monomer)

Fig. S8. Enhanced mitophagy-associated readouts are not accompanied by ROS accumulation or mitochondrial depolarization in *Prrx1*⁺ SuSCs.

A. Representative fluorescence images of DCFH-DA-based ROS staining in WT, CS, WT+si-*Fzd7*, and positive-control-treated WT *Prrx1*$^+$ SuSCs. Images are shown for visualization of ROS probe fluorescence. Scale bar, 75 μm.

B. Quantification of DCF fluorescence intensity. For the disease-state comparison, WT, CS, WT+positive control, and CS+positive control groups were analyzed. For the knockdown comparison, WT+si-NC, WT+si-Fzd7, and WT+positive control groups were analyzed.

C. Representative fluorescence images of JC-1 staining in WT, CS, WT+si-*Fzd7*, and positive-control-treated WT *Prrx1*$^+$ SuSCs. JC-1 aggregates indicate relatively preserved mitochondrial membrane potential, whereas JC-1 monomers indicate mitochondrial depolarization. Scale bar, 50 μm.

D. Quantification of mitochondrial membrane potential using the JC-1 aggregate/monomer fluorescence ratio. For the disease-state comparison, WT, CS, WT+positive control, and CS+positive control groups were analyzed. For the knockdown comparison, WT+si-NC, WT+si-*Fzd7*, and WT+positive control groups were analyzed.

Data are presented as mean ± SEM from n = 3 independent biological replicates per group. Technical replicate wells were averaged before statistical analysis. For ROS analysis, blank-corrected DCF fluorescence values were expressed relative to the matched control within each independent experiment. For JC-1 analysis, mitochondrial membrane potential was quantified as the JC-1 aggregate/monomer fluorescence ratio. Statistical significance was assessed using pre-specified pairwise comparisons with Šídák's multiple-comparison test. Positive-control groups were analyzed only against their corresponding untreated or control groups. ns, not significant; $^*P < 0.05$, $^{**}P < 0.01$, $^{***}P < 0.001$.

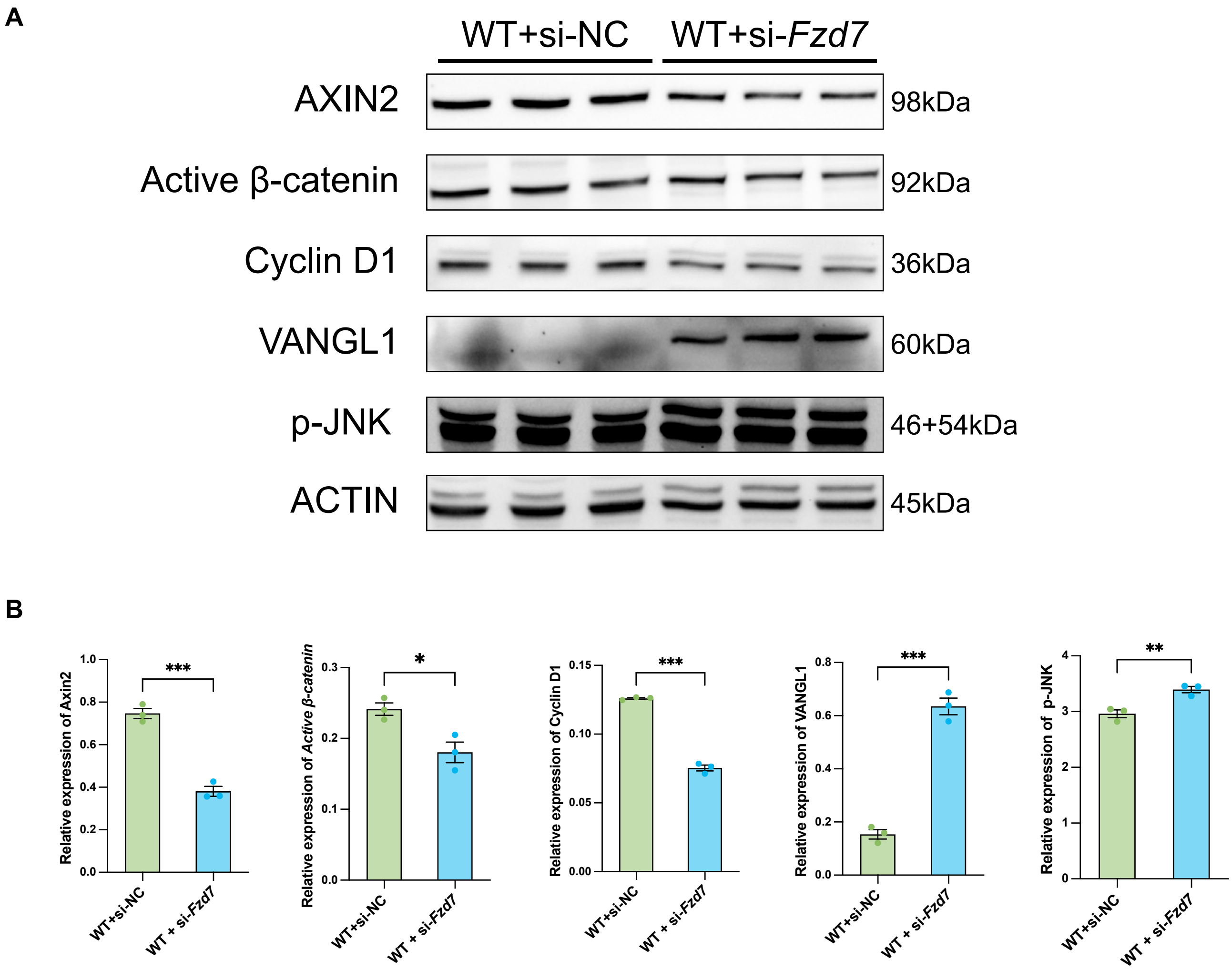
A
WT+si-NC
WT+si-Fzd7
AXIN2
98kDa
Active β-catenin
92kDa
Cyclin D1
36kDa
VANGL1
60kDa
p-JNK
46+54kDa
ACTIN
45kDa
B
Relative expression of Axin2
Relative expression of Active β-catenin
Relative expression of Cyclin D1
Relative expression of VANGL1
Relative expression of p-JNK
***
*
***
***
**
WT+si-NC
WT + si-Fzd7

Fig. S9. *Fzd7* knockdown alters Wnt pathway-associated protein readouts in *Prrx1*⁺ SuSCs.

A. Western blotting analysis of canonical Wnt/β-catenin-associated proteins and PCP/JNK-associated proteins in WT *Prrx1*$^+$ SuSCs treated with non-targeting control siRNA or si-*Fzd7*. Active β-catenin denotes non-phosphorylated β-catenin at Ser45. For p-JNK quantification, the combined densitometric intensity of the p46 and p54 phospho-JNK bands was measured and normalized to ACTIN.

B. Quantification of relative protein levels of active β-catenin, AXIN2, Cyclin D1, p-JNK, and VANGL1. Data are presented as mean ± SEM from n = 3 independent biological replicates per group. Statistical significance was determined by unpaired two-tailed Student's t test unless otherwise indicated. *P < 0.05, **P < 0.01, ***P < 0.001; ns, not significant.

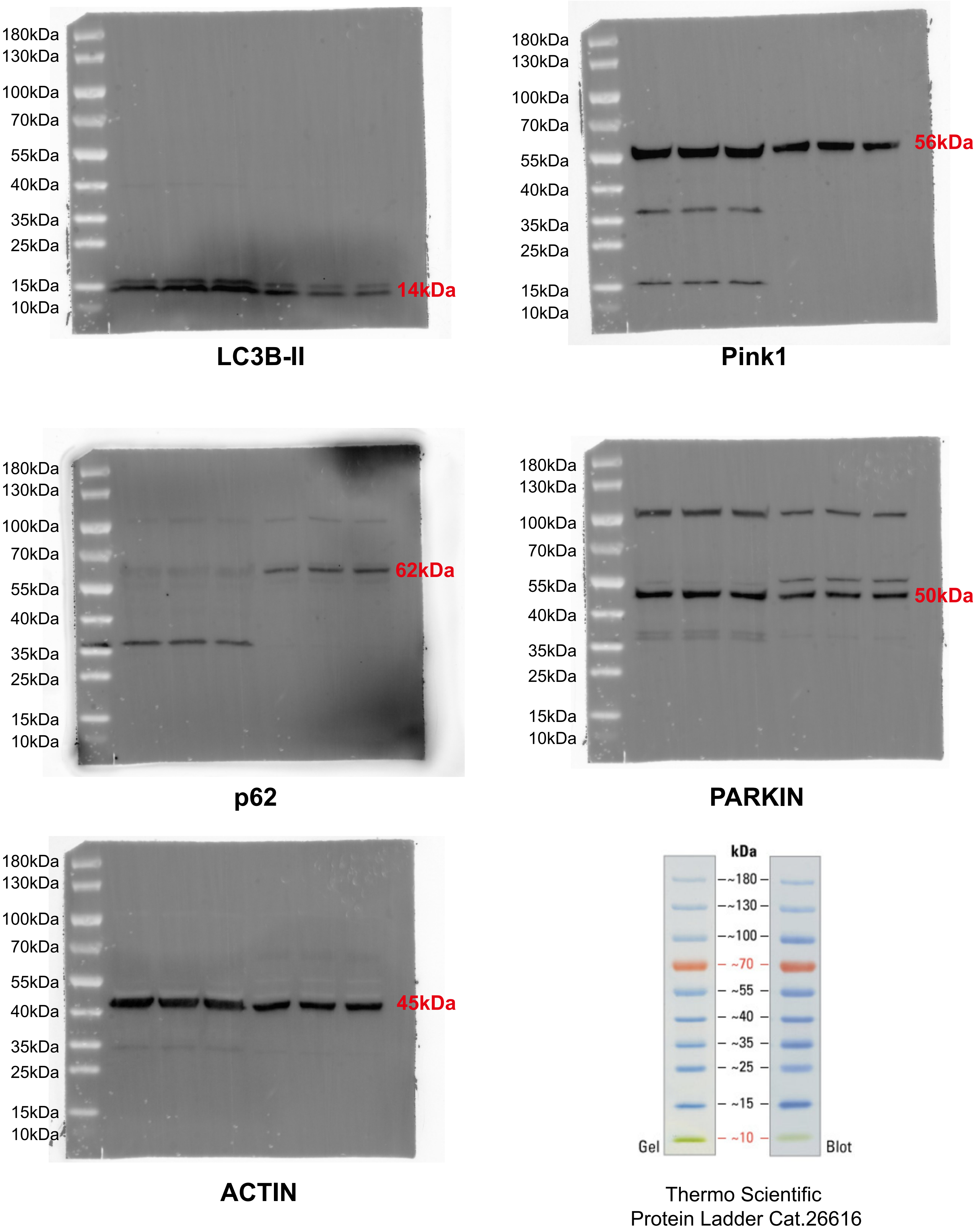

Figure S10. Unedited blot images of Fig. 3C

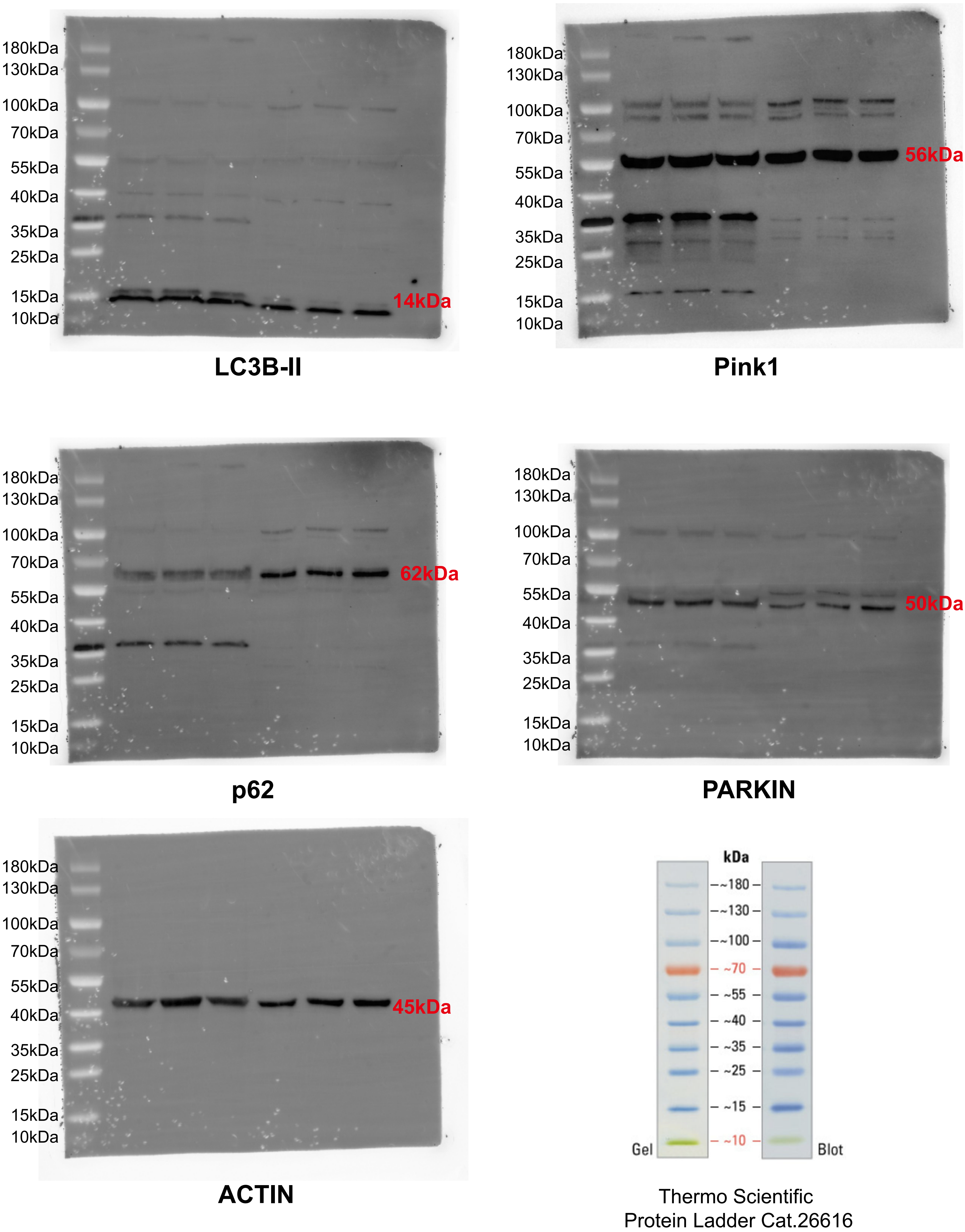

Figure S11. Unedited blot images of Fig. 3G

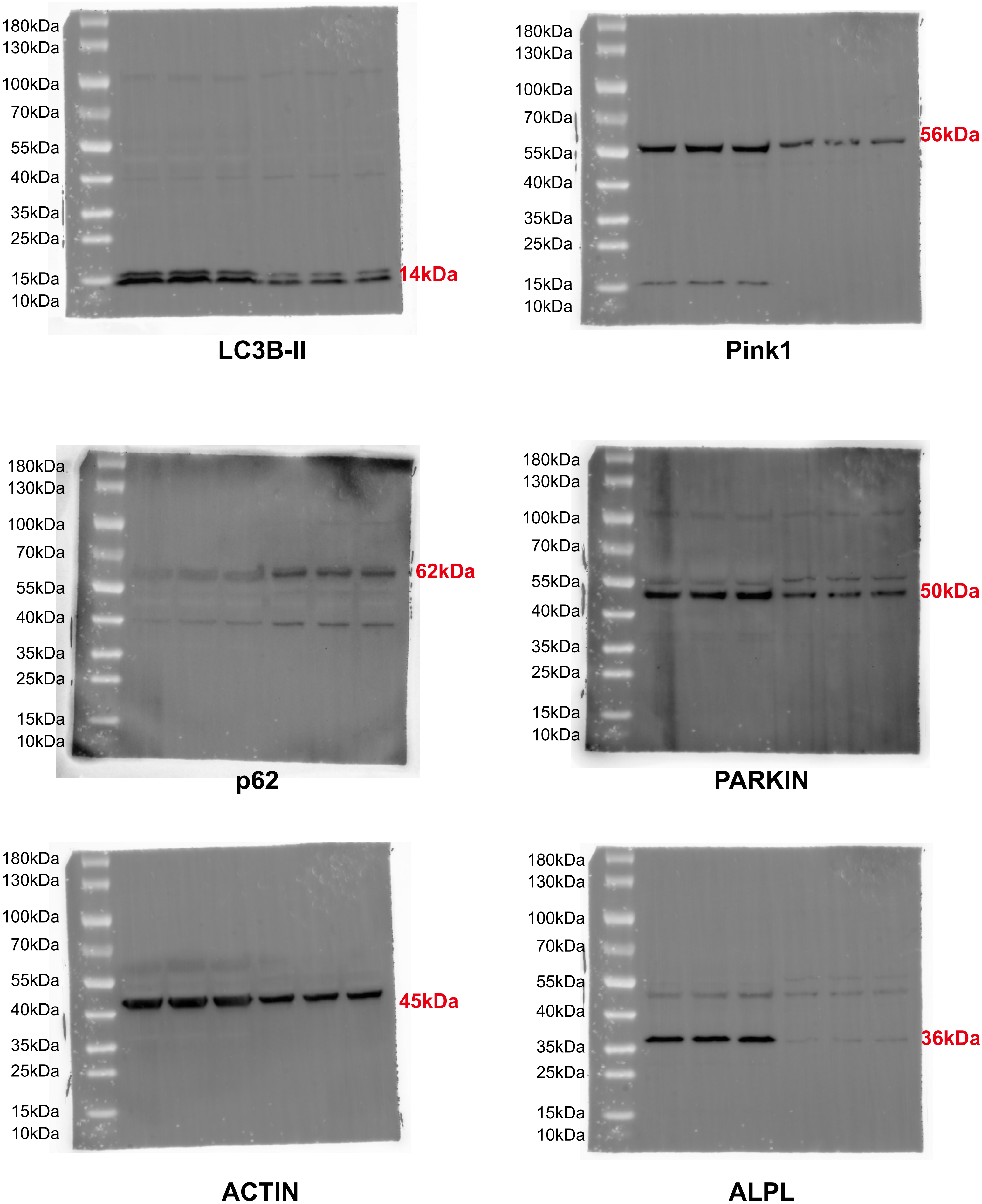

Figure S12. Unedited blot images of Fig. 5C

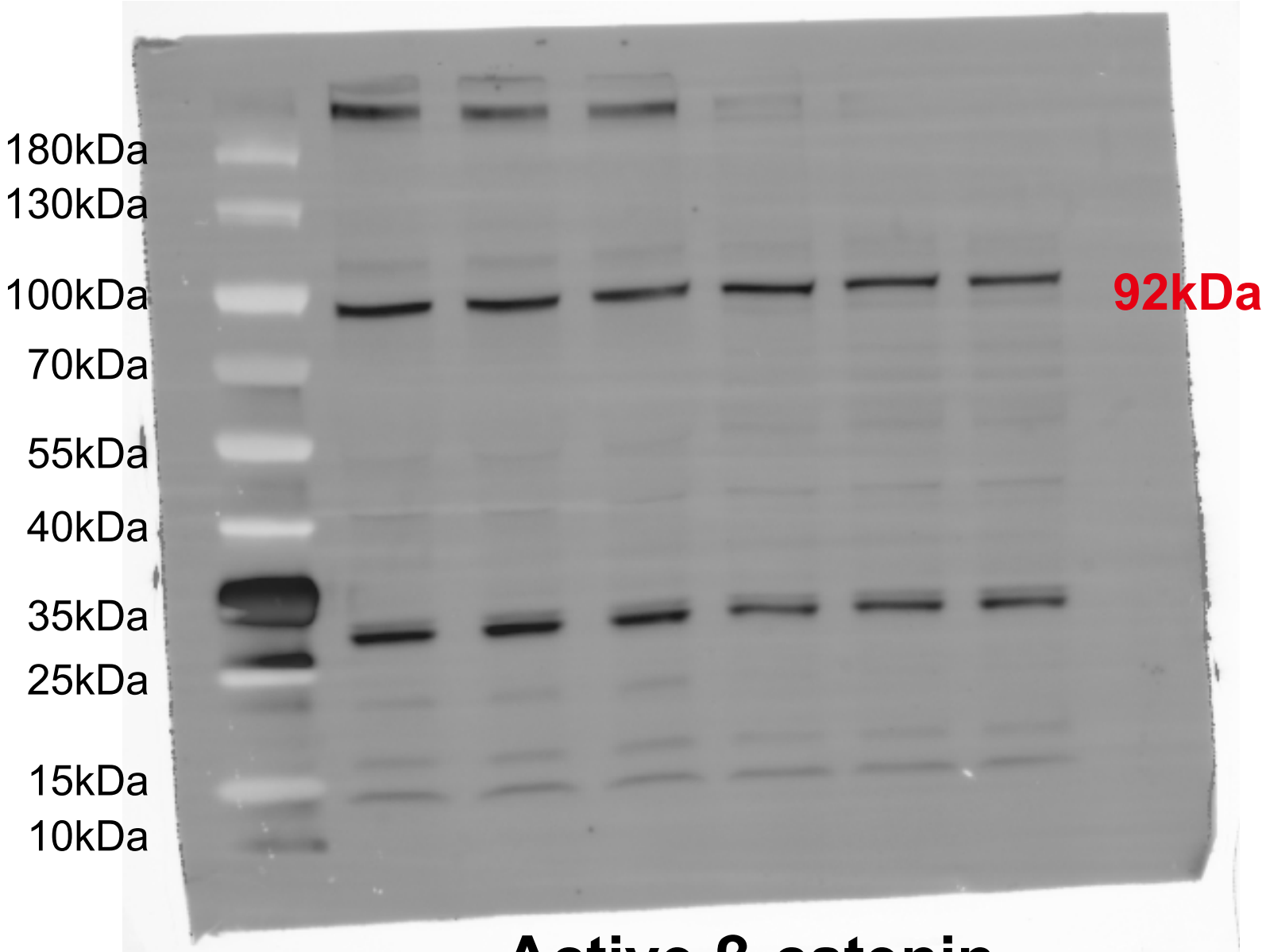

Active β-catenin

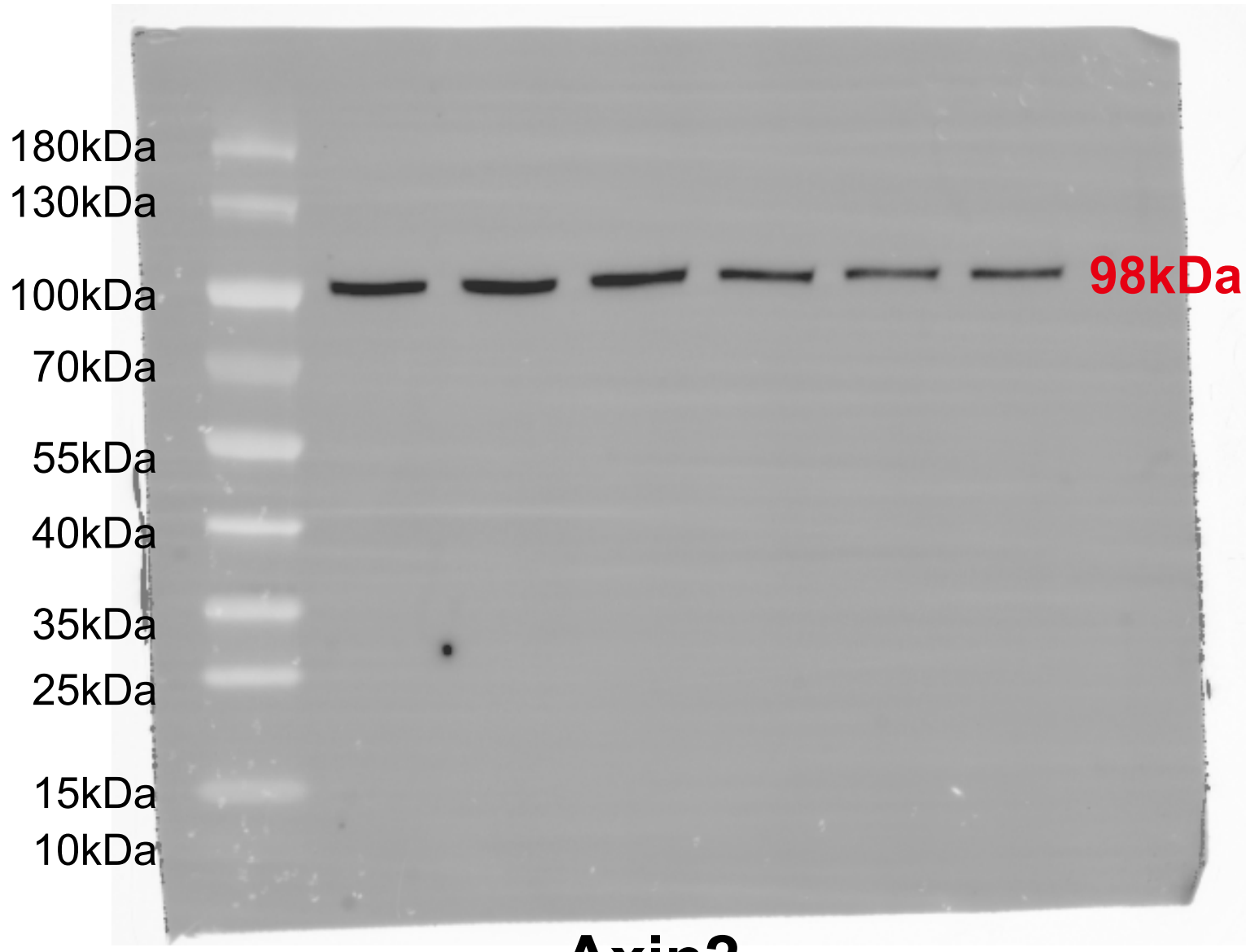

Axin2

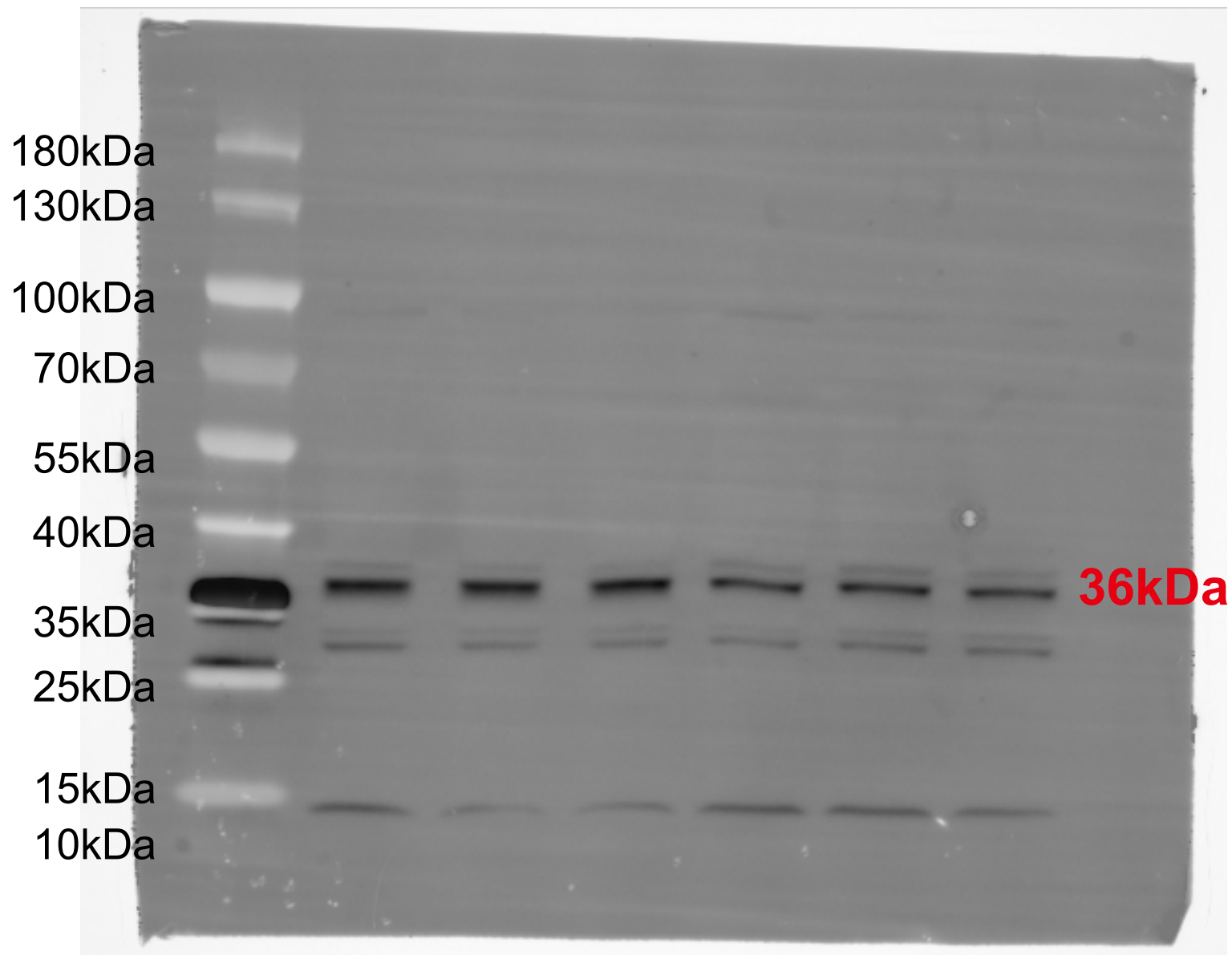

Cyclin D1

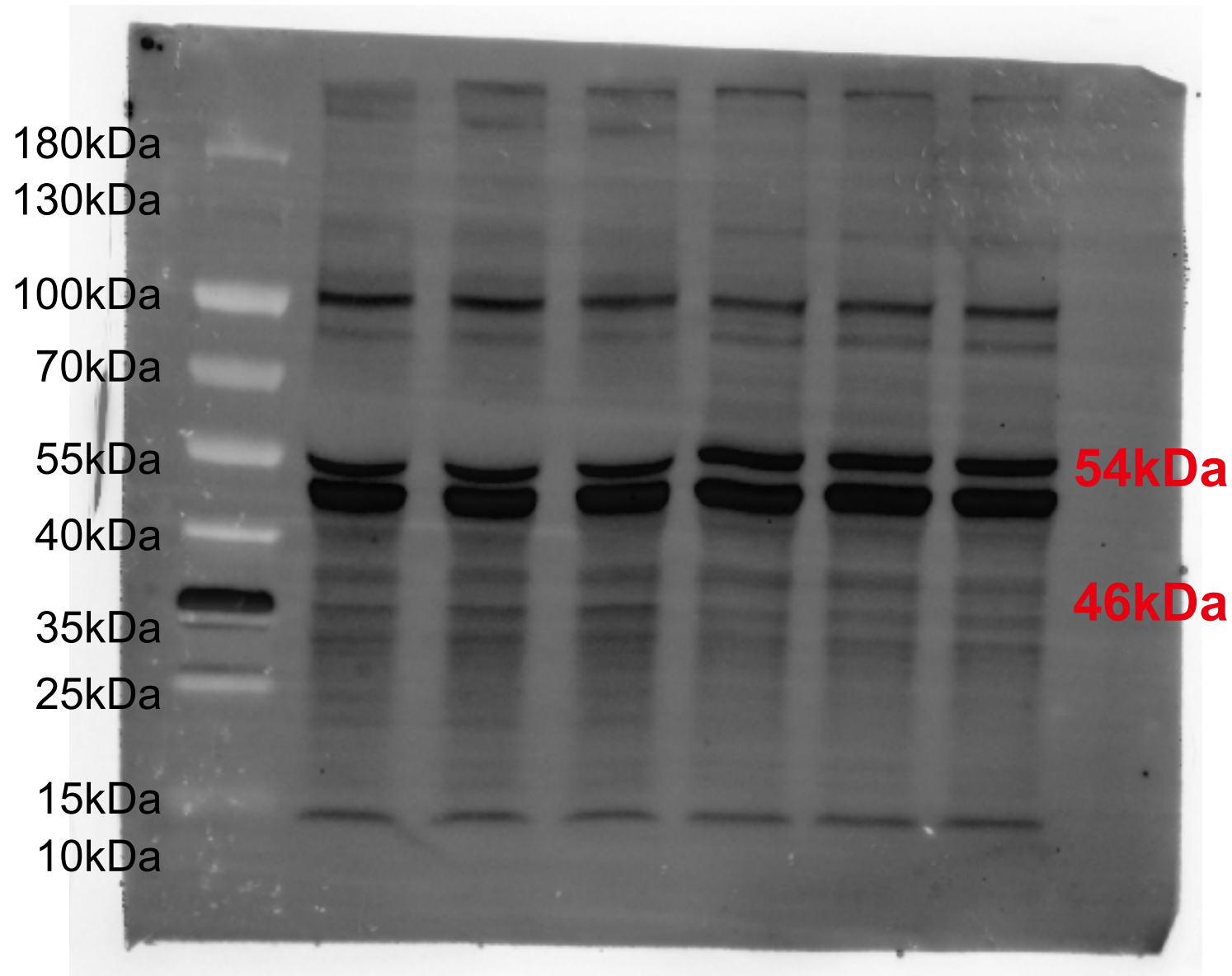

p-JNK

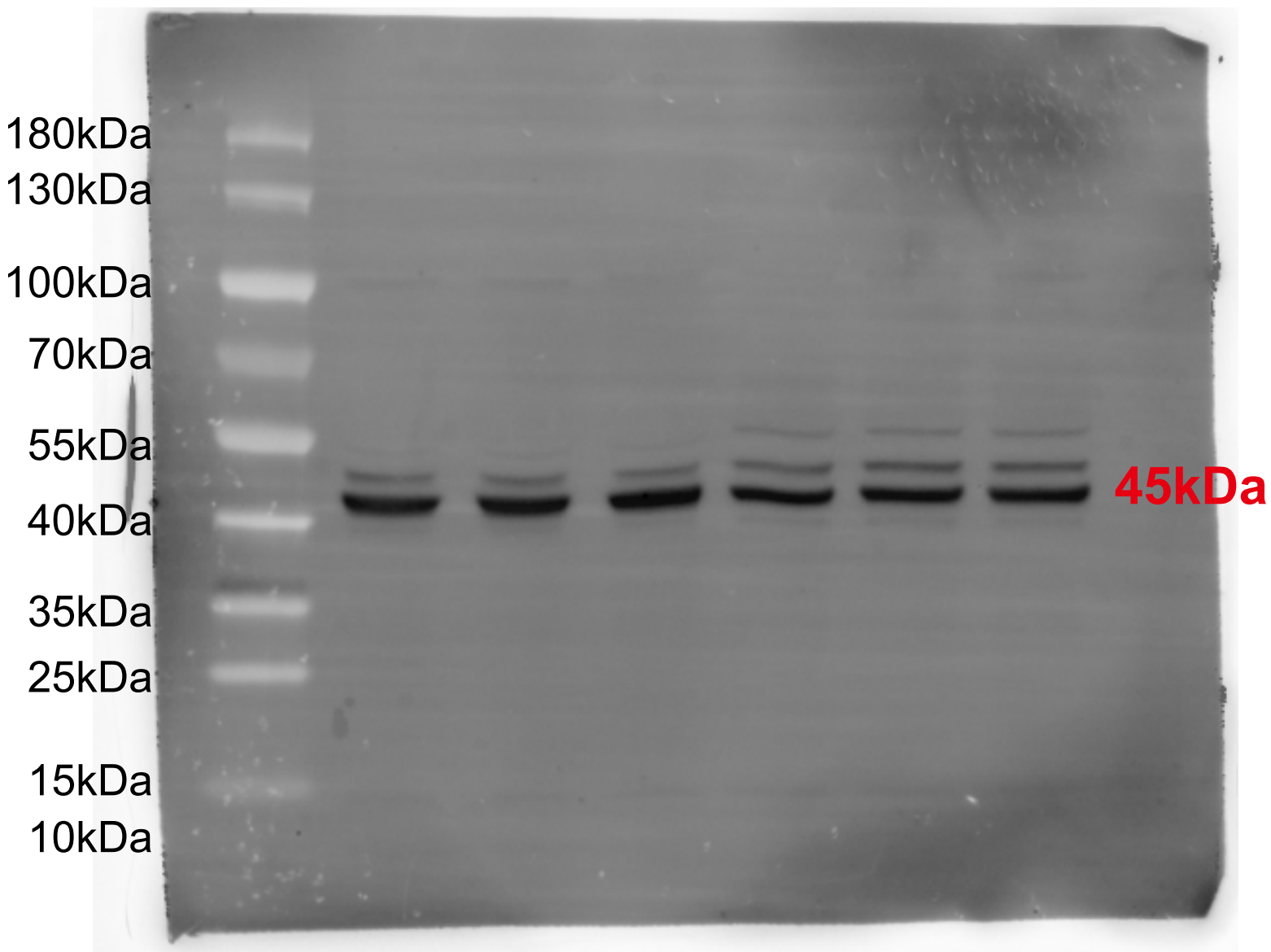

ACTIN

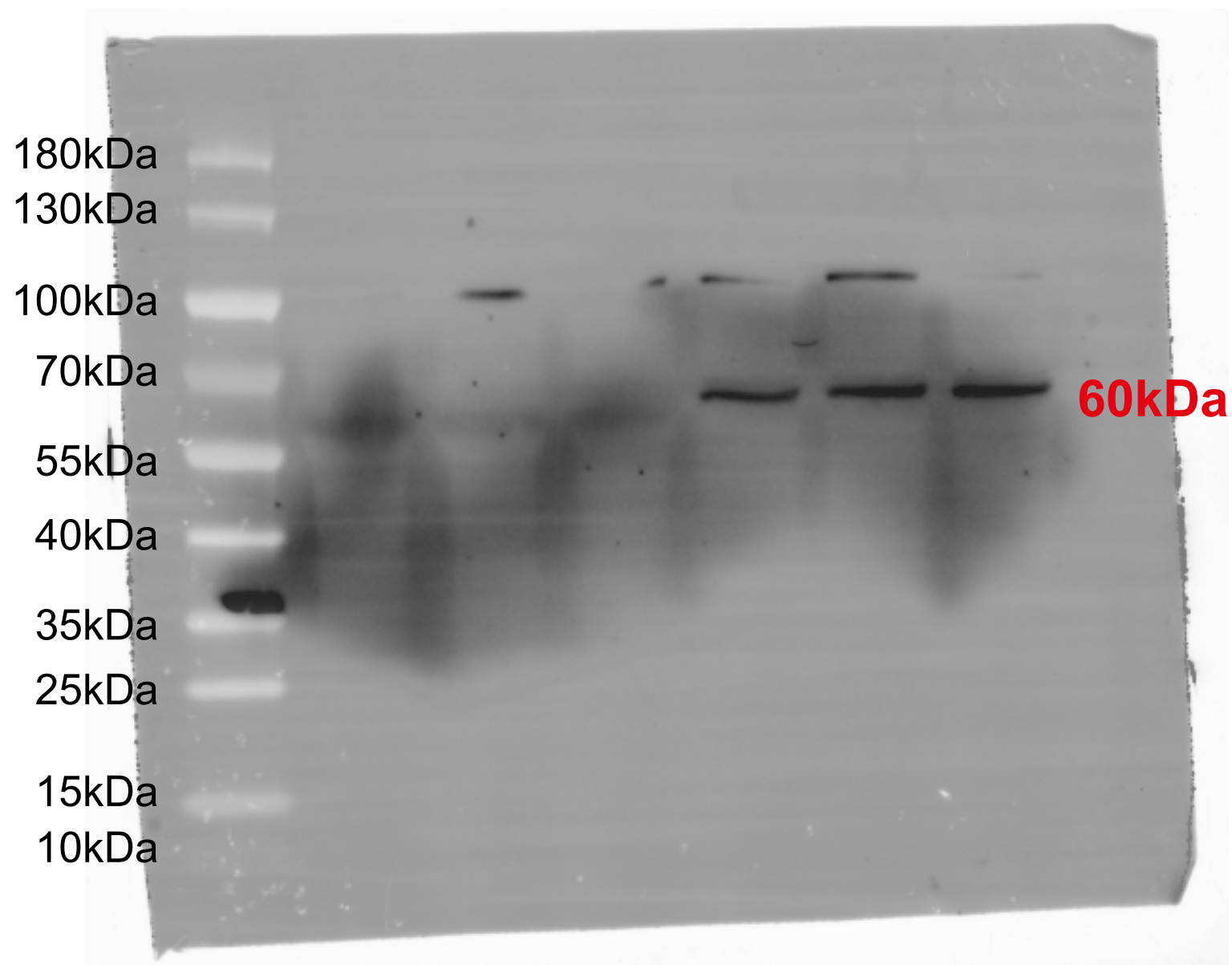

VANGL1

Figure S13. Unedited blot images of Fig. S9

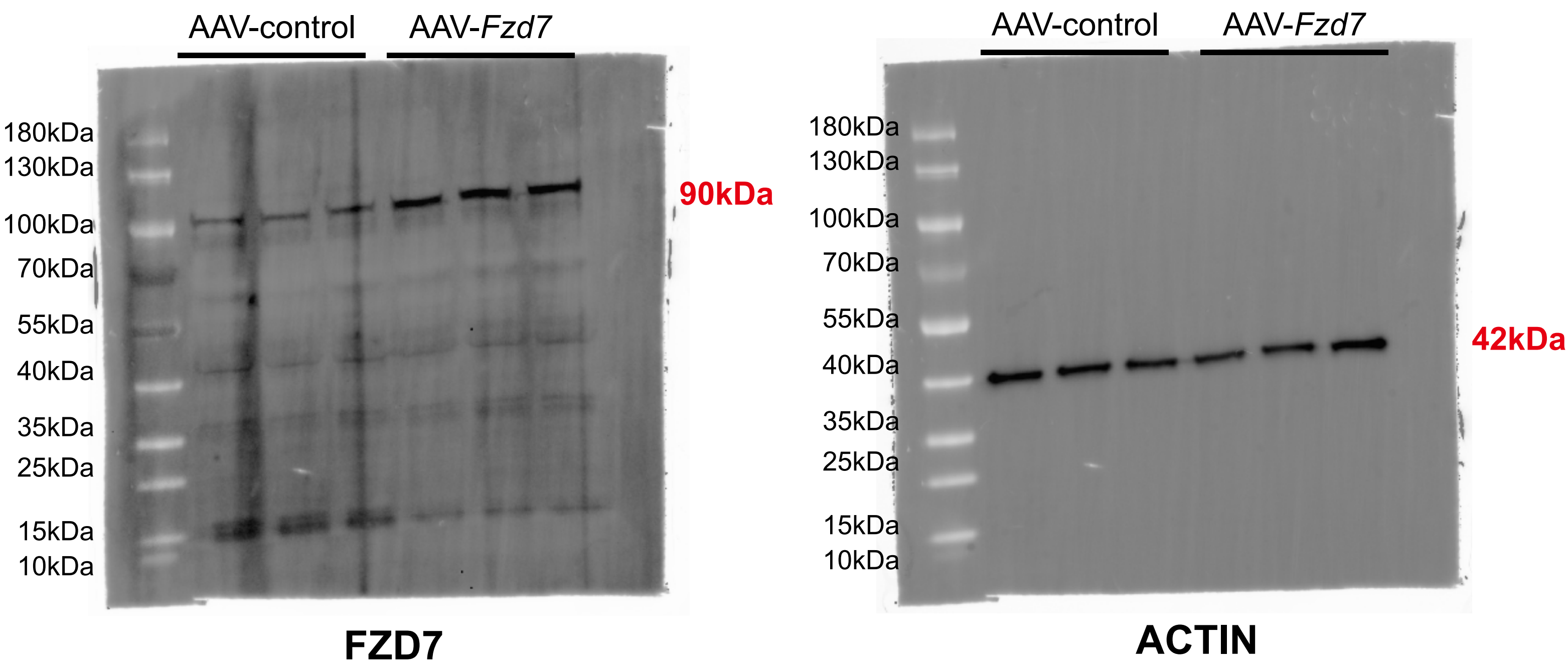

Figure S14. Unedited western blot images of FZD7 validation after AAV-*Fzd7* delivery

Unedited western blot images showing FZD7 protein abundance in the injected coronal suture region 48 h after P0 perisutural delivery of AAV-control or AAV-*Fzd7*. n = 3 per group.

Table S1. Key resources table

REAGENT OR RESOURCE	SOURCE	IDENTIFIER
**Antibodies**		
ALP Antibody (B-10)	Santa Cruz Biotechnology	sc-365765
PINK1 Rabbit Monoclonal Antibody	Cell Signaling Technology	#6946
Parkin Rabbit Monoclonal Antibody	Cell Signaling Technology	#32833
LC3B Antibody	Cell Signaling Technology	#2775
SQSTM1/p62 Antibody	Cell Signaling Technology	#5114
beta-Actin Rabbit Monoclonal Antibody	Cell Signaling Technology	#4970
Active β-Catenin Rabbit Antibody	Cell Signaling Technology	#19807
Axin2 Rabbit Monoclonal Antibody	Cell Signaling Technology	#5863
Cyclin D1 Rabbit Monoclonal Antibody	Cell Signaling Technology	#2978
Phospho-SAPK/JNK Antibody	Cell Signaling Technology	#9251
VANGL1 Rabbit Monoclonal Antibody	Cell Signaling Technology	#14783
Rabbit Anti-Prrx1	Affinity Biosciences	#DF4274
Anti-Frizzled 7 antibody	Abcam	ab64636
Goat Anti-Rabbit IgG, HRP Conjugate	Abcam	ab6721
Goat Anti-Mouse IgG, HRP Conjugate	Abcam	ab6789
Goat Anti-Rabbit IgG, Alexa Fluor 488	Abcam	ab150081
Goat Anti-Rabbit IgG, Alexa Fluor 647	Abcam	ab150083
**Chemicals, peptides, and recombinant proteins**		
Collagenase A	Roche	10103586001
HEPES	Solarbio	H8090
Penicillin-Streptomycin (10,000 U/mL)	Gibco	15140-122
Fetal Bovine Serum	VivaCell	C04001-500
PBS	Servicebio	G4202
DPBS	Gibco	14190144
DMEM	Corning	10-014-CV
DMEM	ThermoFisher	31600
Accutase	Gibco	A1110501
0.5M EDTA	Beyotime	ST069
DAPI	Thermo Scientific	62248
EDTA decalcified solution	Biosharp	BL616B
Triton X-100	Beyotime	ST1723
Antibody dilution buffer	Beyotime	P0269
Mounting Medium with DAPI	Abcam	ab104139
Goat Serum	Beyotime	C0265
Paraformaldehyde fix solution	Beyotime	P0099
PVDF membrane	Millipore	IPVH00010
ECL solution	Thermo Scientific	34579
DMSO	MedChemExpress	HY-Y0320
**Critical commercial assays**		
Osteogenic Differentiation Kit	OriCell	MUXMX-90021
BCA protein assay kit	Beyotime	P0010

Lipofectamine 3000 Transfection Reagent	Thermo Scientific	L3000015
**Recombinant DNA**		
pAAV-CAG-Fzd7-3xFLAG-tWPA	OBiO Technology	H37784
pAAV-CAG-MCS-tWPA	OBiO Technology	GL3037

Table S2. Genotype abbreviations used in the manuscript

Abbreviation or label	Full genotype	Experimental use or purpose
WT	*Fgfr2*$^{+/+}$	Wild-type control
CS	*Fgfr2*$^{C342Y/+}$	Craniosynostosis disease model
Prrx1-EGFP reporter	*Prrx1*$^{EGFP/+}$	Reporter allele for identification or isolation of *Prrx1*$^{+}$ SuSCs
WT; *Prrx1*-EGFP	*Fgfr2*$^{+/+}$; *Prrx1*$^{EGFP/+}$	Reporter-based isolation of WT *Prrx1*$^{+}$ SuSCs
CS; *Prrx1*-EGFP	*Fgfr2*$^{C342Y/+}$; *Prrx1*$^{EGFP/+}$	Reporter-based isolation of CS-derived *Prrx1*$^{+}$ SuSCs
CS; *Prrx1-Fzd7* cKO	*Fgfr2*$^{C342Y/+}$; *Fzd7*$^{fl/fl}$; *Prrx1*$^{CreERT2/+}$	Prrx1-lineage *Fzd7* conditional deletion in the CS context
CS; *Prrx1-Pink1* cKO	*Fgfr2*$^{C342Y/+}$; *Pink1*$^{fl/fl}$; *Prrx1*$^{CreERT2/+}$	Prrx1-lineage *Pink1* conditional deletion in the CS context
CS; *Prrx1-Fzd7*; *Pink1* dKO	*Fgfr2*$^{C342Y/+}$; *Fzd7*$^{fl/fl}$; *Pink1*$^{fl/fl}$; *Prrx1*$^{CreERT2/+}$	*Fzd7*/*Pink1* double conditional deletion for in vivo genetic epistasis
Reporter-bearing *Fzd7*, *Pink1*, or double conditional cohorts	Corresponding conditional genotype plus *R26*$^{tdTomato/+}$	FACS isolation of *Prrx1*$^{+}$ SuSCs for ex vivo experiments
Intermediate breeder lines	*Fzd7*$^{fl/fl}$; *Prrx1*$^{CreERT2/+}$<br>*Pink1*$^{fl/fl}$; *Prrx1*$^{CreERT2/+}$<br>*Pink1*$^{fl/fl}$; *R26*$^{tdTomato/+}$, and related lines	Colony expansion and generation of conditional knockout and reporter-bearing cohorts

Table S3. Genotyping primers

Mouse line/allele	Primer	Sequence
Fgfr2$^{C342Y/+}$	outer (Forward)	TGCAGTTGGAATCTCCTGATGG
	outer (Reverse)	GCCCACTGAGTCAACAATTCAGC
	inner (Reverse)	CGATAGAATTACCCGCCAGGT
Fzd7$^{fl/+}$	A (Forward)	CAAGCCCGACGCTTCTTCTA
	B (Reverse)	TTGAGTCTGATCGGTGCTCG
Prrx1$^{CreERT2/+}$	P1 (Forward)	TCTCCTTTGTCCCTACAGCG
	P2 (Reverse)	CTCCGCCATGGTCCCAATA
	P3 (Forward)	ACAGCGCCATGGCTACTTAT
	P4 (Reverse)	ATTCAACTTGCACCATGCCG
Pink1$^{fl/+}$	I (Forward)	GGCATTCCCTACACGGAGTC
	II (Reverse)	CAGGCCCATCTCAGGGTTTT
	III (Forward)	ATGGCCTGATATGCCGACAC
	IV (Reverse)	ATCTTGCCAGGCCATCCTTC
Rosa26$^{tdTomato/+}$	P1 (Reverse)	TCAGATTCTTTTATAGGGGACACA
	P2 (Forward)	TAAAGGCCACTCAATGCTCACTAA
	P3 (Reverse)	CTGTACGGCATGGACGAGC
	P4 (Forward)	GTTGCGTCAGCAAACACAGT
Prrx1$^{EGFP/+}$	P1 (Forward)	TTAGCGAAGTAACCGAGGCT
	P2 (Reverse)	TTCTTAACGCCTTGTCTCCCC
	P3 (Forward)	CTGTTTGCCTAACTCCCCCG
	P4 (Reverse)	GGCGGACTTGAAGAAGTCGT

Table S4. siRNA Sequence

Source	Identifier	Gene symbol	Sequence
GenePharma	Fzd7-Mus-1100	Fzd7	S: GAGCUACGCUUCUUCUUAUTT<br>AS: AUAAGAAGAAGCGUAGCUCTT
GenePharma	Pinkl-Mus-946	Pink1	S: CUGGCUGACUAUCCUGAUATT<br>AS: UAUCAGGAUAGUCAGCCAGTT
Santa Cruz Biotechnology	sc-37007	N/A	Control siRNA
